# Influenza forecasting for French regions combining EHR, web and climatic data sources with a machine learning ensemble approach

**DOI:** 10.1371/journal.pone.0250890

**Published:** 2021-05-19

**Authors:** Canelle Poirier, Yulin Hswen, Guillaume Bouzillé, Marc Cuggia, Audrey Lavenu, John S. Brownstein, Thomas Brewer, Mauricio Santillana

**Affiliations:** 1 INSERM, U1099, Rennes, France; 2 Université de Rennes 1, LTSI, Rennes, France; 3 Department of Social and Behavioral Sciences, Harvard T.H. Chan School of Public Health, Boston, MA, United States of America; 4 Innovation Program, Boston Children’s Hospital, Boston, MA, United States of America; 5 CHU Rennes, Centre de Données Cliniques, Rennes, France; 6 Université de Rennes 1, Faculté de médecine, Rennes, France; 7 INSERM CIC 1414, Université de Rennes 1, Rennes, France; 8 IRMAR, Institut de Recherche Mathématique de Rennes, Rennes, France; 9 Department of Pediatrics, Harvard Medical School, Boston, MA, United States of America; 10 Computational Health Informatics Program, Boston Children’s Hospital, Boston, MA, United States of America; The Chinese University of Hong Kong, HONG KONG

## Abstract

Effective and timely disease surveillance systems have the potential to help public health officials design interventions to mitigate the effects of disease outbreaks. Currently, healthcare-based disease monitoring systems in France offer influenza activity information that lags real-time by one to three weeks. This temporal data gap introduces uncertainty that prevents public health officials from having a timely perspective on the population-level disease activity. Here, we present a machine-learning modeling approach that produces real-time estimates and short-term forecasts of influenza activity for the twelve continental regions of France by leveraging multiple disparate data sources that include, Google search activity, real-time and local weather information, flu-related Twitter micro-blogs, electronic health records data, and historical disease activity synchronicities across regions. Our results show that all data sources contribute to improving influenza surveillance and that machine-learning ensembles that combine all data sources lead to accurate and timely predictions.

## Introduction

Influenza is a major public health problem causing up to five million severe cases and 500,000 deaths per year worldwide [1–3]. In France alone, the epidemic of 2018–2019 caused 9,500 deaths. During epidemic peaks, large increases of visits to general practitioners and to emergency departments are observed and often lead to disruptions to healthcare delivery and thus increase the risk of undesirable outcomes in patients with influenza infections. To reduce the impact of influenza outbreaks in the population and to better design timely public health interventions, surveillance systems that produce accurate real-time and short-term forecasts of disease activity may prove to be instrumental.

In France, an important influenza monitoring system was implemented by the Sentinelles network in 1984 [4, 5]. This system centralizes information obtained from a group of volunteer (1,314 in 2018) general practitioners and (116 in 2018) pediatricians that each week report the proportion of patients with Influenza-Like-Illness (ILI, any acute respiratory infection with fever above 38°C, cough and onset within the last ten days) seeking medical attention. Data collection, processing, aggregation and distribution processes of this information, at the national and regional levels, introduce up to three weeks delays in the availability of flu activity information. This temporal data gap prevents public health officials from having the most up-to-date epidemiological information, and thus leads to the design of interventions that do not take into consideration recent changes in disease activity [2, 6]. For example, if estimates were available in real-time, information campaigns and vaccination prevention could be deployed earlier and could lead to greater impact. Additionally, healthcare facilities could be better prepared to respond to unexpected increases in the flux of high-risk patient during time periods of increased disease activity.

With the motivation to alleviate this time delay, mathematical modeling and machine learning approaches have been proposed to produce disease estimates in real time and ahead of healthcare-based surveillance systems in multiple nations around the world. Most of these studies have been designed and tested in developed nations, such as the United States and France, where information on disease outbreaks has been collected historically for decades [2]. Numerous research studies have been conducted on the use of traditional statistical methods, like temporal series or compartmental methods, as well as the inclusion of disparate data sources such as meteorological or demographic data to track flu activity, as discussed in Nsoesie et al. 2014 and Yang and Shaman 2014 [7, 8]. And in recent years, multiple more studies have emerged exploring the use of Internet-based data sources that capture aspects of human behavior and environmental factors to track the spread of diseases. With over 3.2 billion web users, data flows from the internet are huge and of all types. Some studies have used data from Google [2, 3, 9–13], Twitter [14–18] or Wikipedia [19–22] to monitor flu specifically.

One of the first and most prominent studies on the use of internet data for monitoring influenza epidemics is Google Flu Trends (GFT) [23, 24]. This web-based platform, created in 2009 and designed and deployed by Google, used the volume of selected Google search terms to estimate ILI activity in real time. GFT led to multiple prediction errors during the 2009 H1N1 Flu Pandemic (due to changes in people’s search behaviour as a result of the exceptional nature of the pandemic) and later produced large overestimations during the 2012–2013 US flu season (due to the announcement of a pandemic that finally did not appear). These events show the lack of robustness of their algorithm and led to eventual discontinuation of this disease monitoring platform [25]. Since then, multiple research teams have proposed improved methodologies that are capable of extracting information more efficiently from flu-related Google searches and produce improved flu estimates [2, 3, 9–13]. Among these methods, the work of Shihao Yang et al. [2] explored a penalized regression methodology that combines historical flu activity with Google search activity dynamically, called ARGO, to better predict flu.

Additional data sources have been explored to monitor flu activity such as clinicians’ searches, electronic health records (EHR), crowd-sourced flu monitoring apps [26–28]. Among these, electronic health records have been shown to track flu accurately and timely in the US and France [6, 29–31]. Specifically, in United States, Santillana et al. [6] showed that a model leveraging EHR data and a machine learning algorithms was capable to monitor flu activity in multiple spatial resolutions that included the regional level. In France, Poirier et al. [29] similarly showed multiple statistical models that incorporate EHR and Internet-search data, can yield accurate ILI incidence rates in real time at the national level.

In early 2019, Fred S. Lu et al. [9] extended the ARGO methodology to accurately track flu activity in multiple states of the United States. In their approach, they included Google search data, EHRs and historical flu trends. They developed also a spatial network approach, called Net, to capture the synchronicity observed historically in flu activity between each states. Finally, by dynamically combining estimates from ARGO and Net, they showed that an ensemble approach, named ARGONet, led to improved results.

### Our contribution

In this study, we propose a forecasting platform that combines multiple data sources and statistical models to track flu activity in France at a spatial resolution that, to our knowledge, has not been explored before. Our forecasting platform produces accurate region-specific real-time and short-term flu activity forecasts for the twelve continental French regions. In our approach, we incorporated data sources such as Google data or Twitter microblogs, Electronic Health Record data, and weather that were not considered in the US study [9]. In addition, the EHR Data used here came directly from a clinical data warehouse rather than cloud-based billing and EHR company which required integrating structured and unstructured clinical data. Additionally, historical synchronicities across regions are captured with a Network model. A machine learning ensemble approach is proposed to improve predictions by dynamically combining estimates from these two distinct approaches. Near real-time estimates as well as one- and two-week ahead forecasts are presented.

## Materials and methods

All the data used for this research were fully anonymized. For the EHR data, the IRB ethics committee from the Rennes Academic Hospital approved this research (Approval number 16.69) and the data were fully anonymized before we accessed them. All other data sources are publicly available and appropriately anonymized. The data data collected from Google and Twitter complied with the terms and conditions for each website.

### Data sources

#### Sentinelles network data

We obtained weekly ILI incidence rates (per 100,000 inhabitants) for the French regions (twelve) from the French Sentinelles network (websenti.u707.jussieu.fr/sentiweb). We retrieved these data in August 2018 from 05 January 2004 to 13 March 2017. We considered these data as the gold standard and as our task for our prediction models.

#### Google data

We obtained the frequency per week of the 100 most correlated internet queries (if correlation ≥ 0.60) by French users from Google Correlate (https://www.google.com/trends/correlate). Because our prediction period spans 05 January 2015 to 20 February 2017, we utilized the ILI signal for each French region, from January 2004 to December 2014 to obtain the most highly correlated search terms using the tool Google Correlate. In this way, we obtained different search terms for each individual region. The signals obtained correspond to queries performed by French users at the national level. We retrieved Google Correlate data in August 2018 for the period going from 05 January 2004 to 13 March 2017.

#### Electronic health record data

We retrieved EHR data from the clinical data warehouse (CDW) of Rennes University Hospital (France). This CDW, called eHOP, integrates structured (laboratory test results, prescriptions, ICD-10 diagnoses) and unstructured (discharge letter, pathology reports, operative reports) patients’ data. It includes data from 1.2 million inpatients and outpatients and 45 million documents that correspond to 510 million structured elements. eHOP consists of a powerful search engine system that can identify patients with specific criteria by querying unstructured data with keywords, or structured data with querying codes based on terminologies.

The first approach to obtain eHOP data connected with ILI was to perform different manual queries to retrieve patients who had at least one document in their EHR that matched the following search criteria: (1) Queries directly connected with flu or ILI with the keywords “flu” or “ILI”; (2) Queries connected with flu symptoms with the keywords “fever”, “pyrexia”, “body aches” or “muscular pain”; (3) Queries connected with flu drugs with the keyword “Tamiflu”; (4) Queries with the ICD-10 terminology; (5) Queries connected with flu tests, positive or negative results.

In total, we performed 34 manual queries. For each query, the eHOP search engine returned all documents containing the chosen keywords (often, several documents for one patient and one stay). For query aggregation, we kept the oldest document for one patient and one stay and then calculated, for each week, the number of stays with at least one document mentioning the keyword contained in the query.

From the CDW eHOP, we built a database containing the time series constructed from the structured data. In all, we have 1,335,347 time series. As Google Correlate, the Pearson correlation between each signal of each region and the time series from the database was calculated. In this way, for each region, the second approach was to retrieve the 100 most correlated signals to ILI signal. Because our test period is from 05 January 2015 to 20 February 2017, we calculated the correlation between January 2004 and December 2014.

As a result, for each region, we obtained 134 variables from the CDW eHOP where there are at least 34 variables common to all regions (manual queries). We retrieved retrospective data in August 2018 for the period going from 03 January 2005 to 13 March 2017.

#### Weather data

We obtained region-specific weather data from the French climatological website Info Climat (https://www.infoclimat.fr). It has been shown in several studies that humidity is correlated with the spread of influenza [32]. In the absence of humidity data on the Climat website, we retrieved precipitation and temperature data. This choice was made knowing that both variables [33, 34], can be used as a proxy for humidity since they are directly related by the Clausius–Clapeyron relation [35]. We obtained temperatures and precipitations per day for the largest city of each region, and calculated the weekly mean for both temperature and precipitation. We retrieved climatic data in August 2018 for the time period going from 07 January 2008 to 13 March 2017.

#### Twitter data

Geotag tweets were extracted as the national scale for France from Boston Children’s Hospital Geotweet dataset with the following keywords pertaining to influenza (“grippe”, “grippé”, “syndrome grippal”, “fièvre”, “toux”, “congestion”, “malade”, “faiblesse”, “courbatures”, “tamiflu”, “la crève”). From there, we aggregated tweets to get weekly counts. In this way, we obtained 11 variables from Twitter. We retrieved Twitter data in December 2018 for the period going from 30 December 2013 to 13 March 2017.

### Statistical models

#### The ARGO model

The ARGO model is a regularized regression dynamically calibrated weekly using the LASSO method [36] to combine multiple external data sources with historical flu information. We performed the LASSO regression with the R package caret and the associated function fit with the method glmnet [37, 38]. We optimized the shrinkage parameter lambda via a ten-fold cross-validation. To test the stationarity and whiteness of residuals, we used Dickey Fuller’s and Box-Pierce’s tests available from the R packages tseries and stats [39]. The formulation of our model is:

- Real time estimates:
  $$\begin{array}{c} y_{it}=\sum_{j=1}^{52}\eta_{j}y_{it-j}+\sum_{k=1}^{10}\alpha_{k}x_{kit}+\sum_{l=1}^{10}\beta_{l}z_{lit}+\sum_{p=1}^{11}\gamma_{p}v_{pt}+\sum_{m=1}^{2}\delta_{m}w_{mit}+\epsilon_{it} \end{array}$$
- One-week ahead forecast:
  $$\begin{array}{c} y_{it+1}=\sum_{j=1}^{52}\eta_{j}y_{it-j}+\sum_{k=1}^{10}\alpha_{k}x_{kit}+\sum_{l=1}^{10}\beta_{l}z_{lit}+\sum_{p=1}^{11}\gamma_{p}v_{pt}+\sum_{m=1}^{2}\delta_{m}w_{mit}+\epsilon_{it+1} \end{array}$$
- Two-week ahead forecast:
  $$\begin{array}{c} y_{it+2}=\sum_{j=1}^{52}\eta_{j}y_{it-j}+\sum_{k=1}^{10}\alpha_{k}x_{kit}+\sum_{l=1}^{10}\beta_{l}z_{lit}+\sum_{p=1}^{11}\gamma_{p}v_{pt}+\sum_{m=1}^{2}\delta_{m}w_{mit}+\epsilon_{it+2} \end{array}$$

where *y*_*it*_ corresponding to the flu incidence rate at time *t* for the region *i*, $\sum_{j=1}^{52}\eta_{j}y_{it-j}$ corresponding to the historical flu incidence rates for the region *i*, $\sum_{k=1}^{10}\alpha_{k}x_{kit}$ corresponding to the 10 most correlated variables from Google data for the region *i*, $\sum_{l=1}^{10}\beta_{l}z_{lit}$ corresponding to the 10 most correlated variables from hospital data for the region *i*, $\sum_{p=1}^{11}\gamma_{p}v_{pt}$ corresponding to Twitter data, $\sum_{m=1}^{2}\delta_{m}w_{mit}$ corresponding to climatic data for the region *i*, *ϵ*_*t*_ corresponding to residuals. We applied this model for each region. The model was dynamically recalibrated every week by incorporating all data available. In this way, the size of our training dataset increases every week. We obtained estimates from January 2011 to March 2017.

#### The Net model

The Net model is a LASSO model dynamically calibrated weekly and using the relationship between the regions to know how synchronicity could improve forecasts. Indeed, S1 Fig in S1 File (Heatmap of pairwise correlations between all regions) shows that the flu incidence rates of the different areas are correlated. For each region, we used historical data of all regions and estimates obtained with ARGO model for all regions expected the region to be predicted. The formulation of our model is:

- Real time estimates:
  $$\begin{array}{c} y_{it}=\sum_{l=1}^{2}\sum_{j=1}^{12}\alpha_{j}y_{jt-l}+\sum_{{j=1}_{j\neq i}}^{12}\beta_{j}{\hat{y}}_{jt}+\epsilon_{it} \end{array}$$
- One-week ahead forecast:
  $$\begin{array}{c} y_{it+1}=\sum_{l=1}^{2}\sum_{j=1}^{12}\alpha_{j}y_{jt-l}+\sum_{{j=1}_{j\neq i}}^{12}\beta_{j}{\hat{y}}_{jt+1}+\epsilon_{it+1} \end{array}$$
- Two-week ahead forecast:
  $$\begin{array}{c} y_{it+2}=\sum_{l=1}^{2}\sum_{j=1}^{12}\alpha_{j}y_{jt-l}+\sum_{{j=1}_{j\neq i}}^{12}\beta_{j}{\hat{y}}_{jt+2}+\epsilon_{it+2} \end{array}$$

where *y*_*it*_ corresponding to the flu incidence rate at time *t* for the region *i*, $\sum_{l=1}^{2}\sum_{j=1}^{12}\alpha_{j}y_{jt-l}$ corresponding to two weeks of historical flu incidence rates for all regions, $\sum_{{j=1}_{j\neq i}}^{12}\beta_{j}{\hat{y}}_{jt}$ corresponding to ARGO predictions for all regions excepted the region *i* to be predicted and *ϵ*_*t*_ corresponding to residuals. We applied this model for each region. We used a two years’ training dataset. We obtained estimates from January 2013 to March 2017.

#### The ARGONet model

The ARGONet model is an ensemble approach combining the predictive power of ARGO and Net models. To combine the results of both models, we tested three methods:

- First, for a given week, we choose ARGO’s estimate if it leads to the lowest mean prediction error in the previous K weeks (compared to the Net model’s estimate). If this is not true, we choose Net’s estimate. The values of K were inspired by Lu et al. [9] study and verified using cross-validation during the training time period.
- A second method consists of calculating the mean value of the estimates produced by the ARGO and Net models for a given week.
- In the final method, for a given week, ARGONet’s estimate is built as a linear combination of estimates produced by ARGO and Net. The coefficients are dynamically calculated each week to best predict new ground truth data available each week.

#### The autoregressive model

To assess the importance of external data sources, we built an autoregressive model of order 52 (AR(52)). We used the LASSO regression with the previous 52 weeks of ILI incidence rates to predict the current week and the two weeks after.

- Real time estimates:
  $$\begin{array}{c} y_{it}=\sum_{j=1}^{52}\alpha_{j}y_{it-j}+\epsilon_{it} \end{array}$$
- One-week ahead forecast:
  $$\begin{array}{c} y_{it+1}=\sum_{j=1}^{52}\alpha_{j}y_{it-j}+\epsilon_{it+1} \end{array}$$
- Two-week ahead forecast:
  $$\begin{array}{c} y_{it+2}=\sum_{j=1}^{52}\alpha_{j}y_{it-j}+\epsilon_{it+2} \end{array}$$

where *y*_*it*_ corresponding to the flu incidence rate at time *t* for the region *i*, $\sum_{j=1}^{52}\alpha_{j}y_{it-j}$ corresponding to the previous 52 weeks, *ϵ*_*t*_ corresponding to residuals. We applied this model for each region. We used a six years’ training dataset. The model was dynamically recalibrated every week.

#### The baseline model

Finally, we included a *baseline model* that simply predicts that the number of new flu cases in a week will be exactly the number of cases observed in the past week.

### Evaluation

Our test period consists on 115 weeks starting from January 2015 to March 2017.

#### Metrics

To assess the performance of the models, we compared estimates to the official incidence rates from the Sentinelles network by calculating two metrics: the root mean squared error (RMSE) and the Pearson correlation coefficient (PCC).

- $RMSE=\sqrt{\frac{1}{n}\sum_{i=1}^{n}{\left(\hat{y_{i}}-y_{i}\right)}^{2}}$
$PCC=\frac{\sum_{i=1}^{n}\left(y_{i}-\bar{y}\right)(\hat{y_{i}}-\bar{\hat{y}})}{\sqrt{\sum_{i=1}^{n}{\left(y_{i}-\bar{y}\right)}^{2}\sum_{i=1}^{n}{\left(\hat{y_{i}}-\bar{\hat{y}}\right)}^{2}}}$

where $\hat{y_{i}}$ is the predicted value for the week *i*, $\bar{\hat{y_{i}}}$ is the mean of predicted values, *y*_*i*_ the real value for the week *i*, $\bar{y_{i}}$ is the mean of real values.

We also estimated the relative efficiency of ARGONet model compared to the autoregressive model AR(52) with 95% confidence interval (CI) by using a Bootstrap method. A relative efficiency, calculated by $\frac{RMSE_{AR52}}{RMSE_{ARGONet}}$ higher that one, suggests increased predictive power of ARGONet compared to the autoregressive model AR(52). The CI and relative efficiency have been computed based on 100 Bootstrap samples of length 52. The 52 weeks were randomly selected from estimates from January 2015 to February 2017.

#### Comparisons

First, we assessed the importance of adding external data sources by comparing:

- RMSE and PCC of the AR(52) model and the ARGO model including historical data plus the ten most correlated variables from hospital data and Google data. The individual contribution of hospital data and Google data has already been shown in a previous study [29]. But, we added in appendices, two comparisons: A comparison with the ten most correlated variables from hospital data and a comparison with the ten most correlated variables from Google data.
- RMSE and PCC of the AR(52) model and the ARGO model including historical data plus climatic data.
- RMSE and PCC of the AR(52) model and the ARGO model including historical data plus Twitter data.

Second, we compared the baseline model, AR(52) model, ARGO model (including all the data sources), Net model and ARGONet model.

## Results

### Evaluation of data sources as predictors

In order to assess the predictive value of each and all external data source, we compared ARGO models that incrementally included external data sources with an autoregressive model, AR(52), model that only uses historical information as input. As shown in the next sections, we found that all external data sources improve flu estimates, specially EHR Data and Google Data.

**EHR data and Google data**. Our first modeling experiment involved comparing ARGO models that use Google search and EHR data simultaneously with the AR(52) in all French regions. A detailed analysis on the individual contribution of Google data and EHR data into predictions, separately, is provided for completeness in the supplementary materials. Our findings suggest that each of these data sources individually improves predictions in all time-horizons. This is consistent with the findings of a previous study conducted at the national-level and the French region of Brittany [29], where both Google and EHR information were found meaningful, but EHR data was shown to possess a stronger predictive power.

The join contribution of both EHR and Google data on predictions is presented below. In real time (Table 1), in terms of correlation, estimates produced using EHR data and Google data improve the accuracy for all the regions and for 9 regions in terms of error metrics. The combination of both sources lead to correlation improvements of up to 5% for the region Bretagne and decreases in error of up to 20% for the region Provence-Alpes-Côte d’Azur.

**Table 1:** Real time estimate—RMSE and PCC for ARGO models including only historical data (AR(52)) and the ten most correlated variables from hospital and Google data, for the period starting from January 2015 to March 2017.

	Auv.	Bour.	Bre.	Cen.	Gd Est	Ht Fra.	Ile Fra.	Norm.	Aqui.	Occi.	Loi.	Pro.
**RMSE**												
AR(52)	**59.02**	36.24	65.41	77.00	**50.05**	**67.52**	61.34	75.29	71.55	58.48	102.38	82.49
ARGO	65.24	**34.99**	**56.83**	**64.52**	52.39	82.30	**56.67**	**64.17**	**65.87**	**50.19**	**97.84**	**67.71**
**PCC**												
AR(52)	0.958	0.898	0.916	0.919	0.952	0.915	0.935	0.879	0.919	0.946	0.815	0.929
ARGO	**0.971**	**0.928**	**0.950**	**0.960**	**0.966**	**0.935**	**0.949**	**0.912**	**0.939**	**0.980**	**0.846**	**0.963**

For one-week ahead estimate (Table 2), estimates obtained with EHR and Google data are more accurate or comparable for eleven of the twelve regions in terms of correlation and nive of the twelve region in terms of error metrics. The combination of both sources lead to correlation improvements of up to 15% for the region Bourgogne and decreases in error of up to 25% for the region Provence-Alpes-Côte d’Azur.

**Table 2:** One-week ahead estimate—RMSE and PCC for ARGO models including only historical data (AR(52)) and the ten most correlated variables from hospital and Google data, for the period starting from January 2015 to March 2017.

	Auv.	Bour.	Bre.	Cen.	Gd Est	Ht Fra.	Ile Fra.	Norm.	Aqui.	Occi.	Loi.	Pro.
**RMSE**												
AR(52)	**105.36**	59.41	99.54	122.54	**83.21**	103.89	101.55	110.63	117.00	102.94	**139.49**	134.43
ARGO	119.89	**57.54**	**84.41**	**97.50**	91.32	**98.36**	**96.13**	**91.69**	**107.28**	**93.53**	153.10	**101.72**
**PCC**												
AR(52)	0.868	0.692	0.782	0.779	0.861	0.782	0.813	0.707	0.758	0.860	**0.645**	0.804
ARGO	**0.896**	**0.827**	**0.881**	**0.903**	**0.909**	**0.875**	**0.847**	**0.813**	**0.834**	**0.923**	0.600	**0.914**

For two-week ahead predictions (Table 3), estimates obtained with EHR and Google data are more accurate for all the regions in terms of correlation and for eleven of the twelve regions in terms of error metrics. The combination of both sources lead to correlation improvements of up to 30% for the region Centre and decreases in error of up to 25% for the region Provence-Alpes-Côte d’Azur.

**Table 3:** Two-week ahead estimate—RMSE and PCC for ARGO models including only historical data (AR(52)) and the ten most correlated variables from hospital and Google data, for the period starting from January 2015 to March 2017.

	Auv.	Bour.	Bre.	Cen.	Gd Est	Ht Fra.	Ile Fra.	Norm.	Aqui.	Occi.	Loi.	Pro.
**RMSE**												
AR(52)	**144.22**	69.89	116.89	155.47	111.85	124.06	129.82	132.51	145.67	128.13	157.68	164.00
ARGO	147.70	**69.30**	**114.15**	**116.30**	**104.74**	**123.84**	**112.41**	**109.75**	**135.96**	**112.28**	**137.85**	**121.01**
**PCC**												
AR(52)	0.731	0.522	0.665	0.609	0.733	0.658	0.688	0.539	0.610	0.731	0.528	0.699
ARGO	**0.820**	**0.708**	**0.779**	**0.832**	**0.834**	**0.823**	**0.775**	**0.714**	**0.720**	**0.849**	**0.664**	**0.858**

**Climatic data**. When combining climatic data with historical activity via ARGO was shown to consistently improve prediction results across all regions (Table 4). However, this improvement is lower than the one observed with EHR and Google data. Indeed, climatic data lead to correlation improvements of 2% for the region Pays de la Loire and decreases in error of 5% for the region Hauts-de-France.

**Table 4:** Real time estimate—RMSE and PCC for ARGO models including only historical data (AR(52)) and only climatic data, for the period starting from January 2015 to March 2017.

	Auv.	Bour.	Bre.	Cen.	Gd Est	Ht Fra.	Ile Fra.	Norm.	Aqui.	Occi.	Loi.	Pro.
**RMSE**												
AR(52)	59.02	36.24	65.41	77.00	50.05	67.52	61.34	75.29	71.55	58.48	102.38	82.49
ARGO	**57.89**	**35.65**	**62.53**	**76.56**	**48.76**	**64.21**	**60.13**	**73.30**	**69.29**	**58.41**	**102.37**	**80.72**
**PCC**												
AR(52)	0.958	0.898	0.916	0.919	0.952	0.915	0.935	0.879	0.919	0.946	0.815	0.929
ARGO	**0.962**	**0.901**	**0.922**	**0.919**	**0.954**	**0.921**	**0.939**	**0.884**	**0.925**	**0.951**	**0.828**	**0.934**

For one-week ahead estimate (Table 5), in term of correlation and error, results obtained with Climatic data are better or comparable for all regions. Climatic data lead to correlation improvements of up to 5% for the region Bourgogne-Franche-Comté and decreases in error of up to 7% for the region Hauts-de-France.

**Table 5:** One-week ahead estimate—RMSE and PCC for ARGO models including only historical data (AR(52)) and only climatic data, for the period starting from January 2015 to March 2017.

	Auv.	Bour.	Bre.	Cen.	Gd Est	Ht Fra.	Ile Fra.	Norm.	Aqui.	Occi.	Loi.	Pro.
**RMSE**												
AR(52)	105.36	59.41	99.54	122.54	83.21	103.89	101.55	110.63	117.00	102.94	139.49	134.43
ARGO	**105.11**	**56.37**	**94.10**	**120.20**	**81.48**	**96.76**	**99.72**	**106.13**	**112.20**	**99.30**	**137.04**	**129.76**
**PCC**												
AR(52)	**0.868**	0.692	0.782	0.779	0.861	0.782	0.813	0.707	0.758	**0.860**	0.645	0.804
ARGO	0.867	**0.726**	**0.809**	**0.788**	**0.867**	**0.808**	**0.825**	**0.740**	**0.787**	0.852	**0.669**	**0.820**

For two-week ahead estimate (Table 6), results obtained with Climatic data are better for all the regions. Climatic data lead to correlation improvements of up to 25% and decreases in error of up to 11% for the region Bourgogne-Franche-Comté.

**Table 6:** Two-week ahead estimates—RMSE and PCC for ARGO models including only historical data (AR(52)) and only climatic data, for the period starting from January 2015 to March 2017.

	Auv.	Bour.	Bre.	Cen.	Gd Est	Ht Fra.	Ile Fra.	Norm.	Aqui.	Occi.	Loi.	Pro.
**RMSE**												
AR(52)	144.22	69.89	116.89	155.47	111.85	124.06	129.82	132.51	145.67	128.13	157.68	164.00
ARGO	**141.10**	**62.27**	**112.50**	**148.99**	**106.08**	**115.28**	**127.36**	**125.57**	**138.09**	**123.71**	**153.15**	**157.00**
**PCC**												
AR(52)	0.731	0.522	0.665	0.609	0.733	0.658	0.688	0.539	0.610	0.731	0.528	0.699
ARGO	**0.743**	**0.651**	**0.709**	**0.647**	**0.759**	**0.719**	**0.701**	**0.610**	**0.655**	**0.758**	**0.590**	**0.726**

**Twitter data**. Overall, we found that national-level flu-related Twitter data improves prediction results for all regions.

In real time (Table 7), we see that between Twitter data and AR(52) results are comparable. Twitter data lead to correlation improvements of 2% for the regions Occitanie and Pays de la Loire and decreases in error of 5% for the region Centre.

**Table 7:** Real time estimate—RMSE and PCC for ARGO models including only historical data (AR(52)) and only Twitter data, for the period starting from January 2015 to March 2017.

	Auv.	Bour.	Bre.	Cen.	Gd Est	Ht Fra.	Ile Fra.	Norm.	Aqui.	Occi.	Loi.	Pro.
**RMSE**												
AR(52)	59.02	**36.24**	65.41	77.00	50.05	67.52	61.34	**75.29**	**71.55**	58.48	**102.38**	82.49
ARGO	**58.86**	36.93	**64.69**	**72.79**	**49.98**	**66.45**	**59.86**	76.72	71.74	**53.66**	102.51	**82.28**
**PCC**												
AR(52)	0.958	**0.898**	**0.916**	0.919	0.952	**0.915**	0.935	**0.879**	0.919	0.946	0.815	0.929
ARGO	**0.960**	0.893	0.914	**0.931**	**0.954**	0.914	**0.945**	0.871	**0.919**	**0.961**	**0.830**	**0.933**

For one-week ahead estimate (Table 8), estimates obtained with Twitter data are more accurate for all the regions in terms of correlation and for ten regions in terms of error metrics. Twitter data lead to correlation improvements of 10% for the region Pays de la Loire and decreases in error of 7% for the region Bretagne.

**Table 8:** One-week ahead estimate—RMSE and PCC for ARGO models including only historical data (AR(52)) and only Twitter data, for the period starting from January 2015 to March 2017.

	Auv.	Bour.	Bre.	Cen.	Gd Est	Ht Fra.	Ile Fra.	Norm.	Aqui.	Occi.	Loi.	Pro.
**RMSE**												
AR(52)	105.36	59.41	99.54	122.54	**83.21**	103.89	**101.55**	110.63	117.00	102.94	139.49	134.43
ARGO	**102.71**	**57.98**	**92.92**	**114.90**	87.64	**101.23**	103.45	**107.90**	**112.16**	**96.11**	**131.67**	**128.60**
**PCC**												
AR(52)	0.868	0.692	0.782	0.779	0.861	0.782	0.813	0.707	0.758	0.860	0.645	0.804
ARGO	**0.881**	**0.712**	**0.821**	**0.833**	**0.873**	**0.787**	**0.846**	**0.723**	**0.811**	**0.883**	**0.703**	**0.834**

For two-week ahead estimate (Table 9), results obtained with Twitter data are more accurate for all the regions in terms of correlation and for nine regions in terms of error metrics. Twitter data lead to correlation improvements of 20% and decreases in error of 6% for the region Bourgogne-Franche-Comté.

**Table 9:** Two-week ahead estimate—RMSE and PCC for ARGO models including only historical data (AR(52)) and only Twitter data, for the period starting from January 2015 to March 2017.

	Auv.	Bour.	Bre.	Cen.	Gd Est	Ht Fra.	Ile Fra.	Norm.	Aqui.	Occi.	Loi.	Pro.
**RMSE**												
AR(52)	**144.22**	69.89	116.89	155.47	**111.85**	124.06	**129.82**	132.51	145.67	128.13	157.68	164.00
ARGO	144.56	**65.52**	**114.06**	**150.85**	115.04	**118.94**	143.15	**130.71**	**143.62**	**124.49**	**153.54**	**160.30**
**PCC**												
AR(52)	0.731	0.522	0.665	0.609	0.733	0.658	0.688	0.539	0.610	0.731	0.528	0.699
ARGO	**0.746**	**0.615**	**0.753**	**0.696**	**0.765**	**0.685**	**0.712**	**0.559**	**0.685**	**0.790**	**0.563**	**0.744**

### Evaluation of statistical models

**Fig 1:**
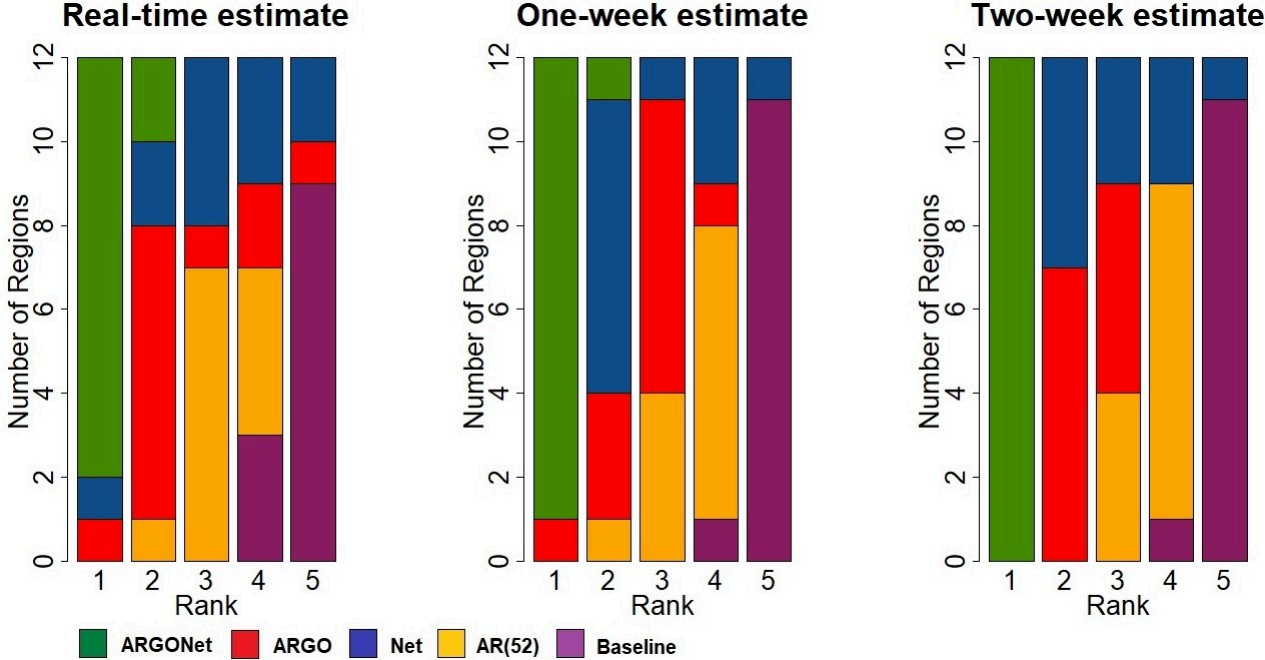
Ranks obtained by each model over the 12 French regions for PCC and RMSE.

Here, we compare the predictive performance of five different modeling approaches the baseline model, AR(52), ARGO, Net, and ARGONet for three time horizons: real-time, one-week and two-week ahead estimates. Fig 1 displays the ranking of each model for each time horizon of prediction across regions during the out-of-sample evaluation time period (January 2015 to March 2017). If a model is ranked in the first position, it means that it led to the best prediction results in terms of error (RMSE) and in most cases this was also the case in terms of correlation. As displayed in Fig 1, ARGONet is the most accurate model, ranking either first or second in all regions in both, real-time estimates and the one-week prediction horizon, and ranking first for the two-week prediction horizon.

**Real-time estimate**. Table 10 summarizes results obtained with the baseline model, AR(52), ARGO, Net and ARGONet models for the period starting from January 2015 to March 2017, for the twelve regions. Over this time period, the 90% confidence interval (CI) of the best correlation is [0.915;0.971] with a median value equal to 0.950. The 90% CI of the blackbest RMSE is [42.97;63.08] with a median value equal to 56.08 These values are mostly obtained with ARGONet model which implies a reduction of the error from 15% to 41% compared to the baseline. The lowest values in term of correlation and the highest values in term of error are obtained with AR(52) and the baseline models. Because public health officials care more about the flu season starting from week 40 to week 15 in the next year, we presented a similar S7 Table in S1 File but calculating correlation and errors only during this time period. We obtained comparable results, with ARGONet model giving the lowest errors and the highest correlations.

**Table 10:** PCC and RMSE for real-time estimate for all french regions for the period starting from January 2015 to March 2017.

	Auv.	Bour.	Bre.	Cen.	Gd Est	Ht Fra.	Ile Fra.	Norm.	Aqui.	Occi.	Loi.	Pro.
**RMSE**												
Baseline	75.89	39.77	65.49	88.96	61.56	70.67	66.72	77.10	74.25	73.99	106.72	95.31
AR(52)	**59.02**	36.24	65.41	77.00	50.05	67.52	61.34	75.29	71.55	58.48	102.38	82.49
Argo	65.14	33.88	**56.39**	63.74	52.30	79.54	**57.10**	**63.08**	65.04	49.51	96.04	64.89
Net	64.46	37.65	70.84	65.35	45.90	**58.76**	68.22	76.19	64.45	57.15	105.28	66.06
K = 1	60.06	33.34	62.10	61.16	49.65	62.58	63.21	66.32	56.31	50.74	100.28	62.86
K = 2	59.58	32.97	61.33	66.30	46.22	65.27	62.50	**65.08**	56.87	49.46	99.33	61.23
K = 3	59.74	33.41	60.25	66.77	**43.79**	63.96	63.36	72.47	**60.46**	49.92	**90.21**	64.72
K = 4	64.77	33.49	63.97	63.51	49.07	70.37	60.07	66.10	**61.07**	49.12	**92.07**	66.28
Mean	61.42	**31.77**	59.27	**59.62**	44.86	60.47	60.07	**65.20**	61.83	**47.06**	**92.05**	**55.77**
Lm	**55.16**	**31.02**	**52.72**	**60.96**	**42.97**	**55.34**	**54.64**	78.06	63.55	**44.55**	100.11	**58.91**
**PCC**												
Baseline	0.936	0.884	0.917	0.897	0.930	0.907	0.927	0.879	0.918	0.925	0.830	0.911
AR(52)	0.958	0.898	0.916	0.919	0.952	0.915	0.935	0.879	0.919	0.946	0.815	0.929
Argo	0.970	**0.930**	**0.951**	**0.958**	**0.966**	0.939	**0.951**	**0.915**	0.941	**0.979**	0.857	0.964
Net	0.964	0.892	0.906	0.942	0.963	0.933	0.928	0.881	0.936	0.956	0.830	0.957
K = 1	**0.971**	0.922	0.932	0.952	0.968	0.935	0.939	0.909	**0.952**	0.971	0.844	0.961
K = 2	**0.971**	0.922	0.934	0.946	0.969	0.931	0.941	**0.911**	**0.952**	0.971	0.845	**0.966**
K = 3	0.970	0.920	0.936	0.946	**0.970**	0.933	0.940	0.887	0.946	0.971	**0.874**	0.961
K = 4	0.967	0.919	0.929	0.948	0.964	0.912	0.945	0.905	0.944	0.975	0.869	0.956
Mean	**0.971**	0.923	0.937	**0.955**	**0.969**	**0.946**	0.944	0.909	0.943	0.974	**0.870**	**0.970**
Lm	0.966	**0.926**	**0.948**	0.952	0.969	**0.940**	**0.948**	0.878	0.938	**0.979**	0.841	0.965

**Fig 2:**
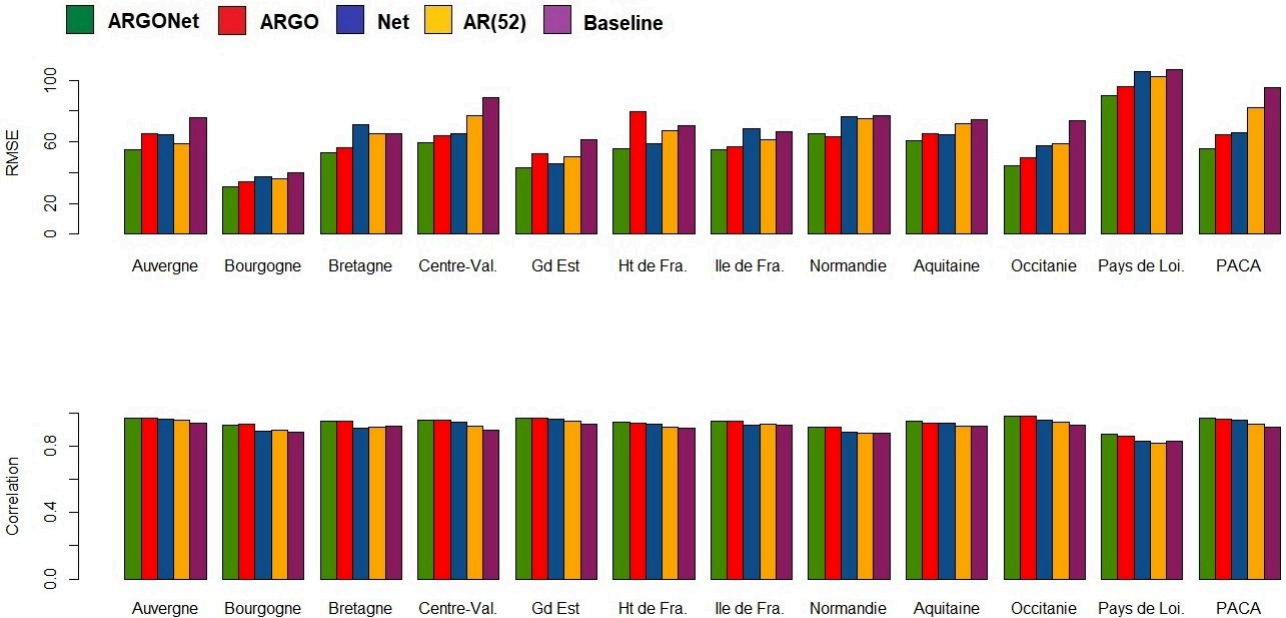
Visualization of correlation and errors obtained for real-time estimate with each model.

**Fig 3:**
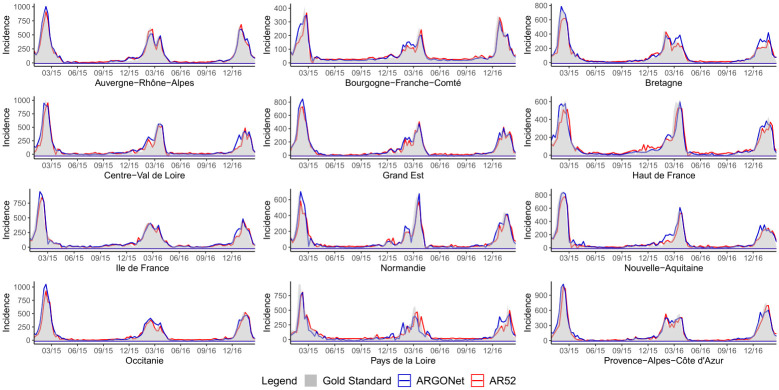
One-week ahead estimate obtained with ARGONet and AR(52) models from January 2015 to March 2017.

**Fig 4:**
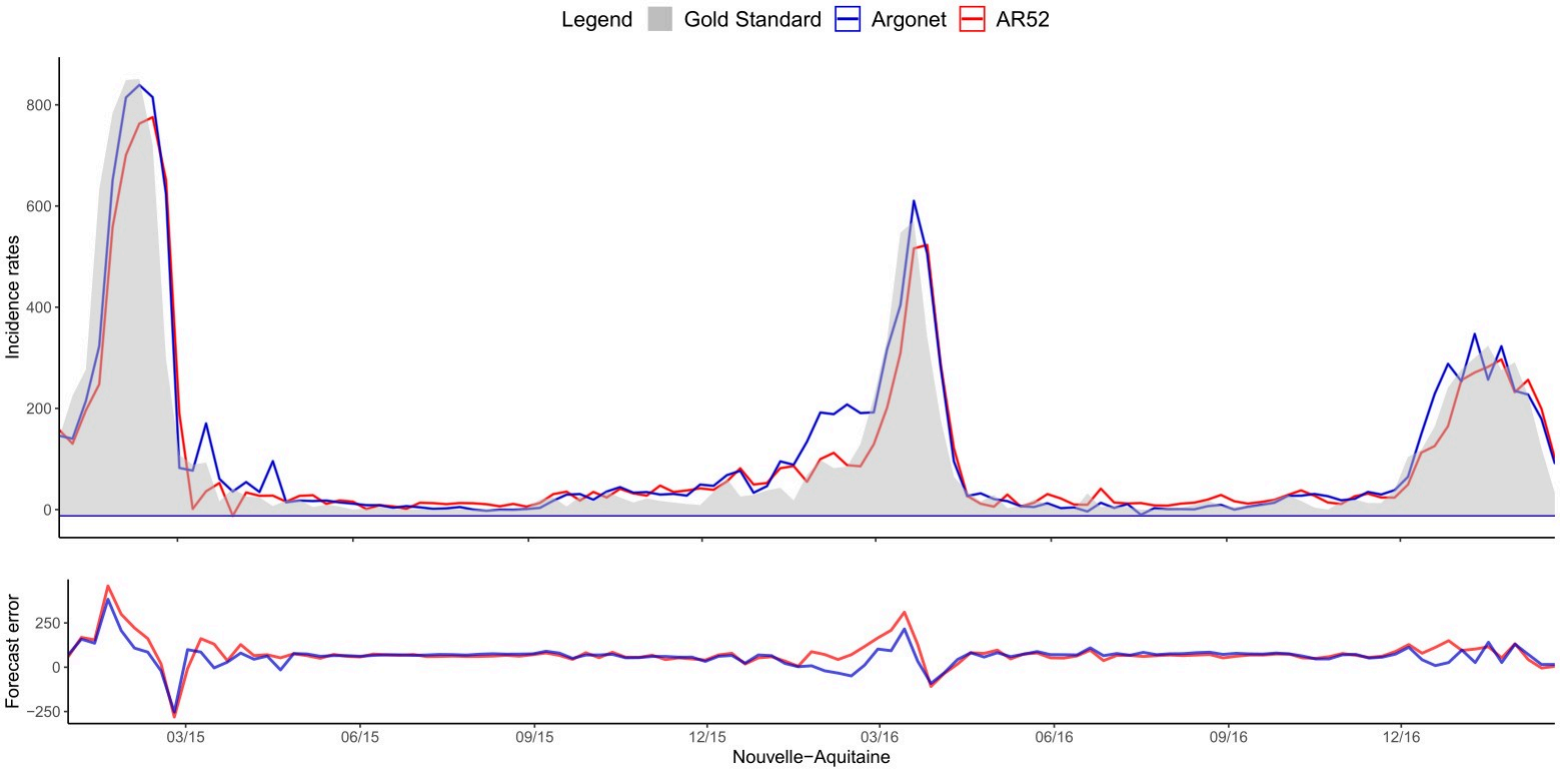
Two-week ahead estimate obtained with ARGONet and AR(52) models from January 2015 to March 2017.

**Fig 5:**
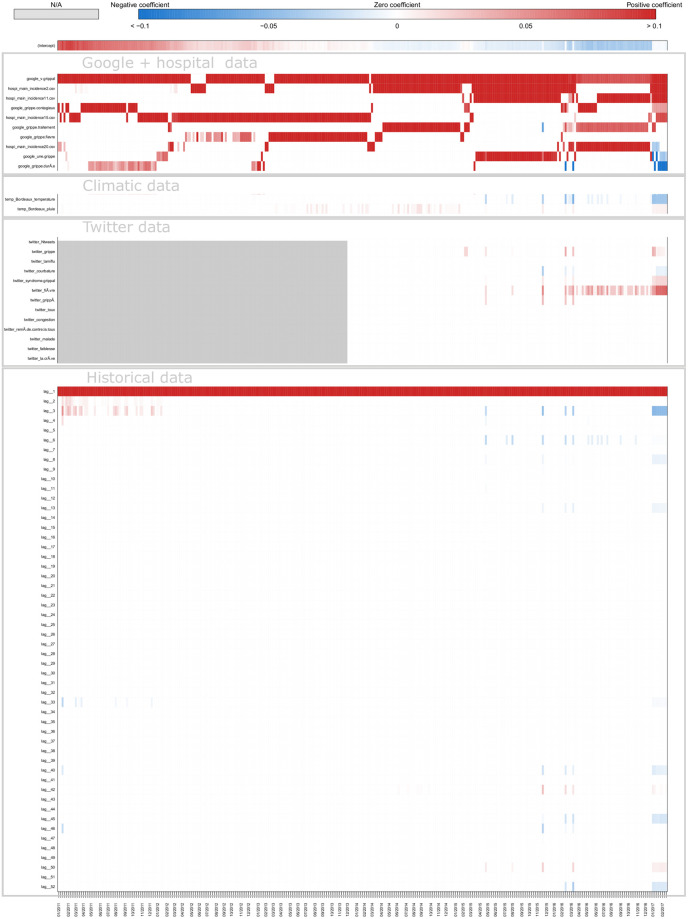
Error distribution.

**Fig 6:**
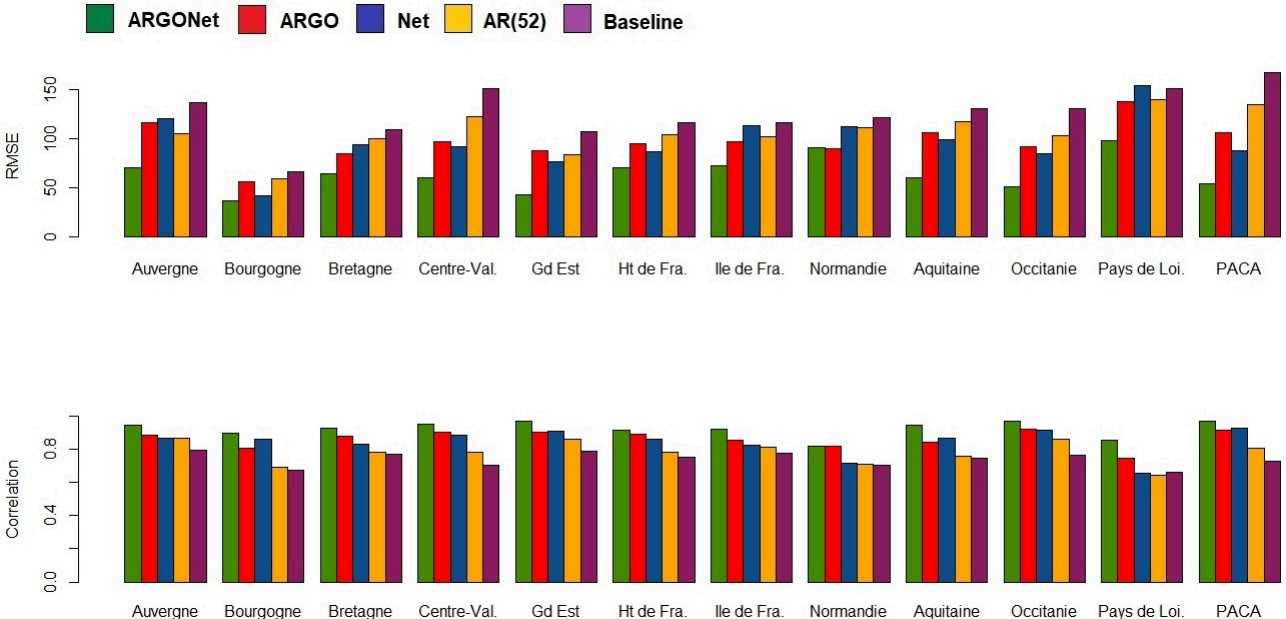
Correlation distribution.

Fig 2 is a visualization of results obtain in Table 10. It confirms, region by region, that the best PCC and RMSE are mostly obtained with ARGONet. Nevertheless, for real-time estimate, ARGO shows good performance. For seven regions ARGO is the model with the second lowest RMSE and for 5 regions, ARGO is the model with the first highest PCC. In comparison, for the one- and two-week time horizons, Figs 3–6 confirm that ARGONet outperforms all other models.

**Fig 7:**
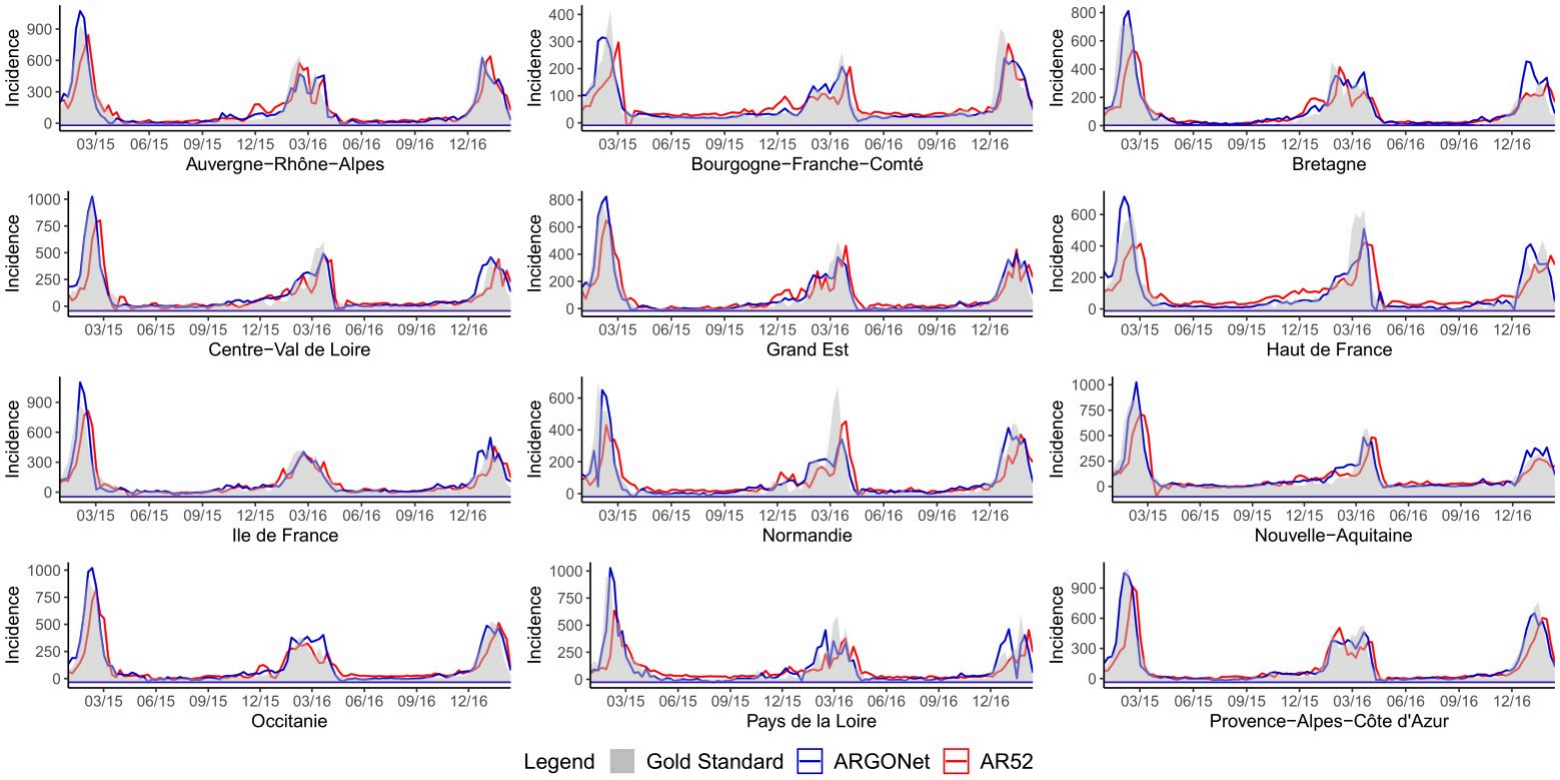
Real-time estimate obtained with ARGONet and AR(52) models from January 2015 to March 2017.

Fig 7 is a visualization of estimates obtain with ARGONet and AR(52) models.

To assess the statistical significance of the improved prediction power of ARGONet, we constructed a 95% confidence interval for the relative efficiency of ARGONet compared to the autoregressive model AR(52) (the error of ARGONet is in the denominator). Table 11 shows that in real-time, the improvement obtained thanks to the ARGONet model compared to the autoregressive model AR(52) is statistically significant for all regions. Depending on the region, ARGONet allows to reduce the error by 8% to 32%.

**Table 11:** Real-time estimate—relative efficiency being higher than one suggests increased predictive power of ARGONet compared to the autoregressive model AR(52).

Region	Relative efficiency	95% CI
Auv.	1.10	[1.06;1.14]
Bour.	1.20	[1.16;1.23]
Bre.	1.11	[1.06;1.15]
Cen.	1.29	[1.25;1.33]
Gd Est	1.17	[1.14;1.21]
Ht Fra.	1.27	[1.22;1.32]
Ile Fra.	1.07	[1.04;1.09]
Norm.	1.13	[1.10;1.17]
Aqui.	1.22	[1.19;1.24]
Occi.	1.40	[1.33;1.47]
Loi.	1.16	[1.13;1.19]
Pro.	1.45	[1.38;1.52]

**Fig 8:**
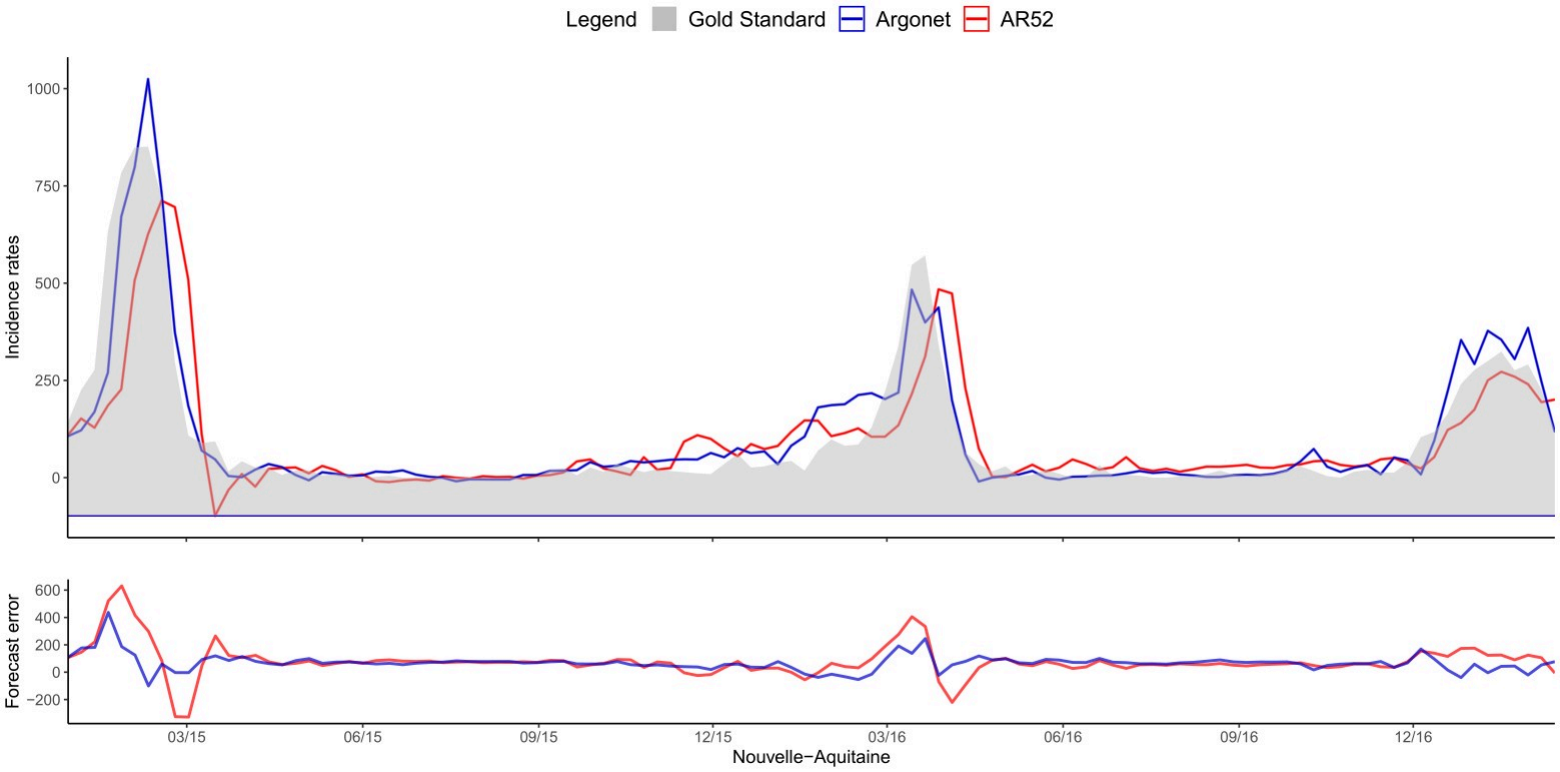
Nouvelle-Aquitaine real time estimate.

**Fig 9:**
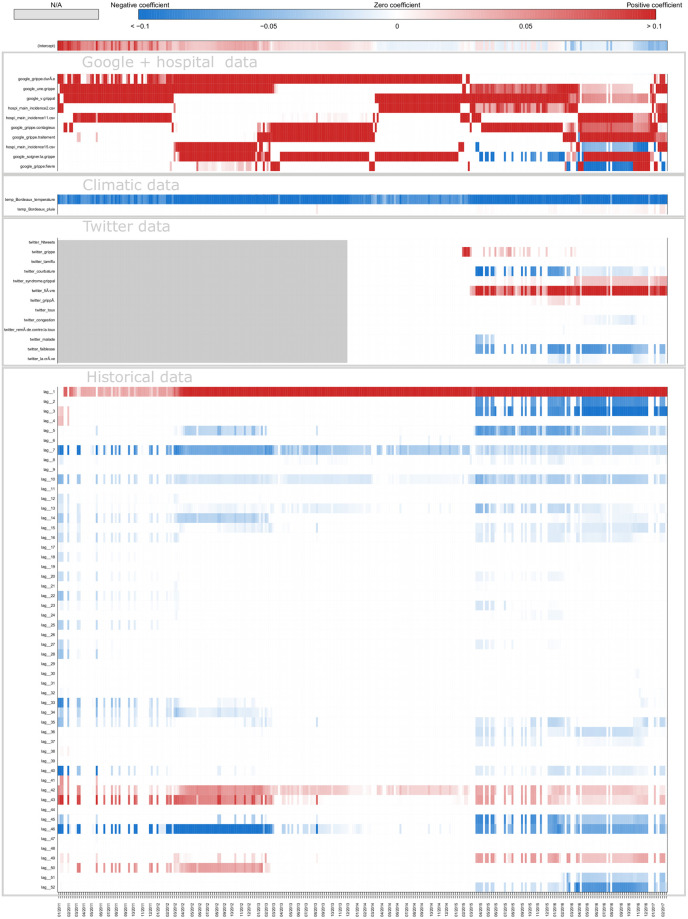
Coefficients Nouvelle-Aquitaine real-time estimate.

Fig 8 shows a typical example of plot obtained for estimates in real time with ARGONet and AR(52) models. This plot allows also us to visualize the forecast error. On this plot, we can see that for the autoregressive model, there is a time lag more important. Fig 9 is a heatmap showing the coefficients used in ARGO model. On the heatmap, we can see that ARGO model uses mostly five variables including two variables from Google Data, two variables from Hospital Data and one variable from Historical Data. Similar plots are presented in Supplementary for all the other regions.

**Fig 10:**
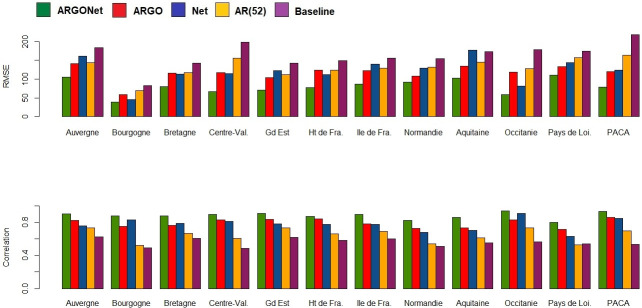
Visualization of correlation and errors obtained for one-week ahead estimate with each model.

**One-week ahead estimate**. Table 12 shows results for one-week ahead estimates for the time period January 2015-March 2017. Over this time period, the 90% CI of the best correlation is [0.852;0.970] with a median value equal to 0.936. The 90% CI of the best RMSE is [43.53;89.45] with a median value equal to 62.435. All these values are mostly obtained with ARGONet model which implies a reduction of the error from 22% to 67% compared to the baseline. In comparison to the best results, AR(52) and the baseline are the models giving the highest errors and lowest correlations. In contrast to real-time estimates, ARGO and Net models are comparable. Indeed, for seven regions, Net model is the model having the second lowest errors. These results can be observed on Fig 10 and on the distribution of correlation and error (Figs 5 and 6). Similar results are also observed for the flu season period (S8 Table in S1 File).

**Table 12:** PCC and RMSE for one-week ahead estimate for all french regions for the period starting from January 2015 to March 2017.

	Auv.	Bour.	Bre.	Cen.	Gd Est	Ht Fra.	Ile Fra.	Norm.	Aqui.	Occi.	Loi.	Pro.
**RMSE**												
Baseline	136.56	66.90	108.59	150.66	106.89	115.98	116.45	120.85	129.93	130.65	150.85	166.71
AR(52)	105.36	59.41	99.54	122.54	83.21	103.89	101.55	110.63	117.00	102.94	139.49	134.43
Argo	115.70	56.25	84.90	96.91	88.17	94.86	96.91	**89.45**	106.28	91.96	137.27	105.82
Net	120.74	42.07	93.78	91.97	76.19	86.38	113.57	112.50	98.63	84.34	154.18	87.44
K = 1	93.47	42.92	77.12	89.46	72.52	83.44	**84.34**	108.51	77.74	86.23	**98.04**	89.48
K = 2	109.39	46.51	78.70	92.20	80.97	92.23	104.61	110.08	84.42	86.52	121.87	84.05
K = 3	111.03	43.67	75.76	94.88	79.74	89.66	96.62	110.53	89.80	85.08	**117.30**	99.06
K = 4	112.71	40.34	69.04	103.34	81.70	89.91	94.96	110.37	79.24	85.63	121.59	105.17
Mean	**75.95**	**39.93**	**64.33**	**59.84**	**48.54**	**71.84**	**72.44**	**90.96**	**60.54**	**51.23**	**117.64**	**54.75**
Lm	**70.84**	**37.46**	**67.18**	**70.46**	**43.53**	**70.23**	85.18	105.60	**62.65**	**61.20**	132.59	**60.61**
**PCC**												
Baseline	0.793	0.672	0.771	0.704	0.788	0.749	0.777	0.702	0.748	0.765	0.660	0.729
AR(52)	0.868	0.692	0.782	0.779	0.861	0.782	0.813	0.707	0.758	0.860	0.645	0.804
Argo	0.881	0.803	0.877	0.903	0.904	0.890	0.856	**0.816**	0.839	0.917	0.747	0.913
Net	0.864	0.861	0.828	0.886	0.906	0.859	0.824	0.716	0.867	0.916	0.654	0.928
K = 1	0.919	0.868	0.899	0.899	0.919	0.875	0.907	0.735	0.926	0.928	**0.852**	0.921
K = 2	0.908	0.854	0.902	0.896	0.910	0.859	0.865	0.727	0.921	0.957	0.787	0.930
K = 3	0.909	0.862	0.901	0.891	0.914	0.863	0.876	0.727	0.903	0.963	**0.801**	0.909
K = 4	0.905	**0.888**	**0.927**	0.885	0.916	0.864	0.884	0.730	0.919	0.955	0.785	0.895
Mean	**0.947**	0.882	**0.922**	**0.953**	**0.966**	**0.913**	**0.922**	**0.816**	**0.945**	**0.970**	0.783	**0.971**
Lm	**0.944**	**0.894**	0.915	**0.936**	**0.965**	**0.904**	**0.909**	0.752	**0.943**	**0.966**	0.728	**0.965**

Fig 3 is a visualization of results obtain with ARGONet and AR(52) models for one-week ahead estimates.

Table 13 shows that the improvement obtained with ARGONet model compared to the autoregressive model AR(52) is statistically significant for all regions for one-week ahead estimates. Depending on the region, ARGONet allows to reduce the error by 18% to 59%.

**Table 13:** One-week ahead estimate—relative efficiency being higher than one suggests increased predictive power of ARGONet compared to the autoregressive model AR(52).

Region	Relative efficiency	95% CI
Auv.	1.47	[1.42;1.52]
Bour.	1.70	[1.62;1.78]
Bre.	1.54	[1.48;1.60]
Cen.	2.07	[1.95;2.19]
Gd Est	1.94	[1.85;2.04]
Ht Fra.	1.51	[1.47;1.55]
Ile Fra.	1.42	[1.37;1.46]
Norm.	1.38	[1.31;1.45]
Aqui.	2.05	[1.94;2.15]
Occi.	1.97	[1.86;2.08]
Loi.	1.45	[1.35;1.54]
Pro.	2.42	[2.30;2.55]

**Fig 11:**
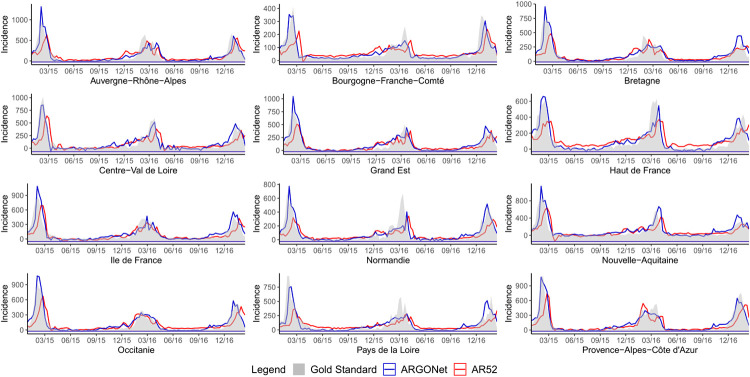
Nouvelle-Aquitaine one-week ahead estimate.

**Fig 12:**
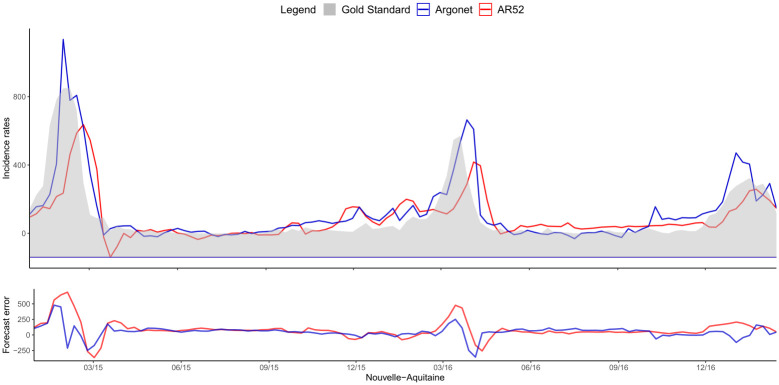
Coefficients Nouvelle-Aquitaine one-week ahead estimate.

Fig 11 shows one-week ahead estimate obtained for the french region Nouvelle-Aquitaine. On this plot, we can see that AR(52) still have a lag of one or two weeks in contrast to ARGONet model. The heatmap on Fig 12, shows that ARGO model uses mostly seven variables including three variables from Google Data, two variables from Hospital Data, one variable from Climatic data and one variable from Historical data.

**Two-week ahead estimate**. Table 14 shows results for two-week ahead estimates for the time period January 2015-March 2017. Over this time period, the 90% CI of the best correlation is [0.825;0.935] with a median value equal to 0.885. The 90% CI of the best relative error is [59.28;105.77] with a median value equal to 79.53. As for real-time and one-week ahead estimates, ARGONet is the model giving the best results in terms of correlation and error whereas AR(52) and the baseline model give the least accurate results. ARGONet allows a reduction of the error from 37% to 67% compared to the baseline. For most of the french regions, the method giving the highest correlation and lowest error for ARGONet is the method using the mean between estimates obtained from ARGO and Net models.

**Table 14:** PCC and RMSE for two-week ahead estimate for all french regions for the period starting from January 2015 to March 2017.

	Auv.	Bour.	Bre.	Cen.	Gd Est	Ht Fra.	Ile Fra.	Norm.	Aqui.	Occi.	Loi.	Pro.
**RMSE**												
Baseline	183.37	83.10	142.04	198.19	143.26	148.72	155.39	154.69	173.17	178.29	175.17	218.07
AR(52)	144.22	69.89	116.89	155.47	111.85	124.06	129.82	132.51	145.67	128.13	157.68	164.00
Argo	140.92	59.58	116.14	116.91	104.27	123.72	122.35	107.48	134.90	119.40	133.39	120.45
Net	161.43	46.06	113.19	114.30	122.32	112.73	140.13	129.32	177.22	81.00	144.54	124.58
K = 1	130.99	44.73	**107.81**	**66.95**	104.91	96.25	115.71	113.29	144.06	**62.30**	124.25	105.48
K = 2	143.37	43.62	115.40	**76.26**	110.01	94.20	120.63	116.80	144.78	67.12	124.46	108.19
K = 3	139.22	43.53	113.27	86.15	116.06	106.26	**110.86**	120.85	151.50	64.75	**113.65**	106.03
K = 4	139.90	43.86	117.70	91.28	119.35	105.56	114.64	113.98	154.33	68.86	113.83	125.38
Mean	**107.89**	**41.48**	**80.62**	87.88	**71.24**	**88.21**	**86.90**	**91.60**	**102.77**	68.05	**111.15**	**78.44**
Lm	**105.77**	**38.58**	120.10	87.78	**91.78**	**77.39**	128.37	119.14	173.22	**59.28**	120.66	**82.49**
**PCC**												
Baseline	0.627	0.494	0.608	0.484	0.616	0.584	0.603	0.508	0.550	0.563	0.538	0.536
AR(52)	0.731	0.522	0.665	0.609	0.733	0.658	0.688	0.539	0.610	0.731	0.528	0.699
Argo	0.823	0.753	0.766	0.829	0.835	0.840	0.779	0.727	0.735	0.829	0.712	0.860
Net	0.759	0.832	0.786	0.814	0.781	0.775	0.775	0.677	0.700	0.905	0.631	0.847
K = 1	0.869	0.844	**0.831**	0.838	**0.865**	0.838	0.850	0.740	**0.826**	0.897	0.688	0.868
K = 2	0.856	**0.861**	0.829	0.846	0.862	0.856	0.857	0.722	0.814	0.904	0.749	0.891
K = 3	0.860	0.845	0.828	0.847	0.853	0.848	0.857	0.723	0.820	0.879	**0.765**	0.855
K = 4	0.857	0.814	0.826	0.832	0.850	0.853	**0.858**	0.741	0.767	0.901	0.750	0.860
Mean	**0.901**	0.857	**0.876**	**0.895**	**0.909**	**0.873**	**0.893**	**0.825**	**0.858**	**0.938**	**0.802**	**0.935**
Lm	**0.886**	**0.876**	0.795	**0.852**	0.833	**0.865**	0.850	**0.745**	0.754	**0.906**	0.744	**0.917**

**Fig 13:**
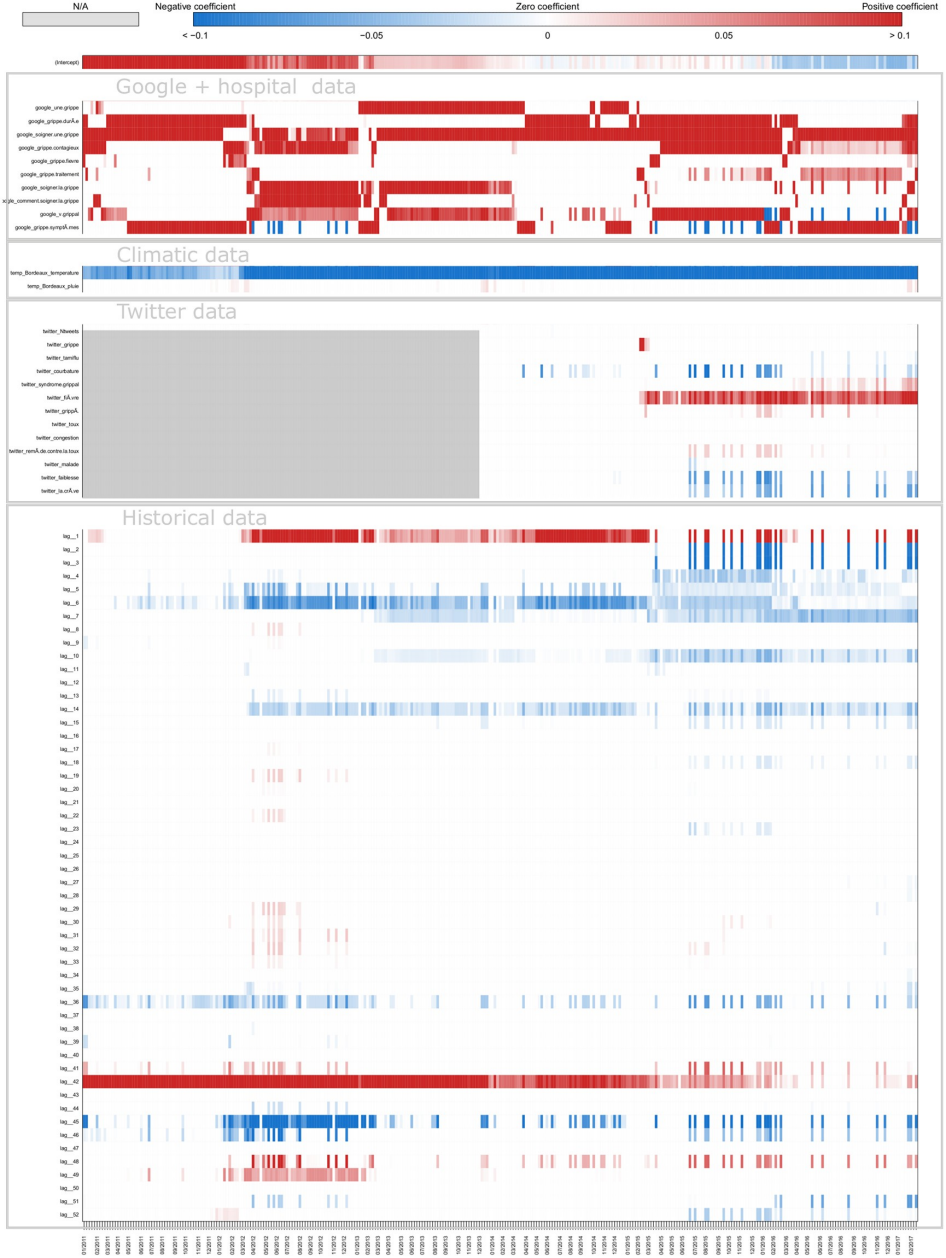
Visualization of correlation and errors obtained for two-week ahead estimate with each model.

Fig 13, allows to visualize that ARGONet is the best model for all regions in term of correlation and error. These results are confirmed with the distribution of correlation and error of each model obtained by calculating the PCC and RMSE for each flu season and each region. (Figs 5 and 6).

Fig 4 allows to visualize estimates obtained with ARGONet and AR(52) models.

Table 15 shows that the improvement obtained with the ARGONet model compared to the AR(52) model is statistically significant for all regions for two-week ahead estimate. Depending on the region, ARGONet allows to reduce the error by 27% to 57%.

**Table 15:** Two-week ahead estimate—relative efficiency being bigger than one suggests increased predictive power of ARGONet compared to the AR(52) model.

Region	Relative efficiency	95% CI
Auv.	1.39	[1.35;1.43]
Bour.	1.91	[1.84;1.98]
Bre.	1.44	[1.38;1.49]
Cen.	2.36	[2.25;2.46]
Gd Est	1.54	[1.51;1.58]
Ht Fra.	1.62	[1.58;1.66]
Ile Fra.	1.49	[1.44;1.53]
Norm.	1.49	[1.45;1.53]
Aqui.	1.44	[1.39;1.49]
Occi.	2.24	[2.15;2.33]
Loi.	1.41	[1.35;1.47]
Pro.	1.99	[1.90;2.08]

**Fig 14:**
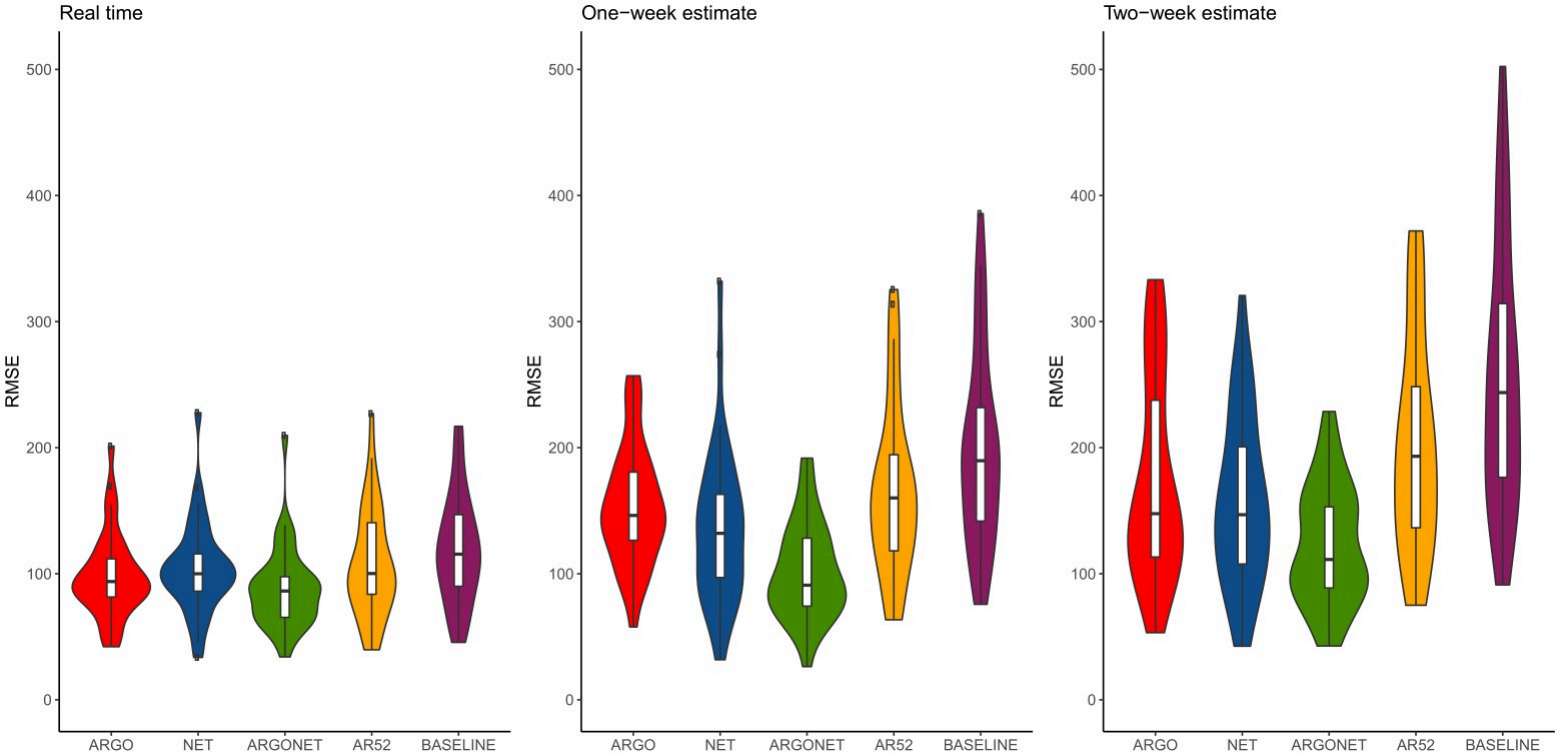
Nouvelle-Aquitaine two-week ahead estimate.

**Fig 15:**
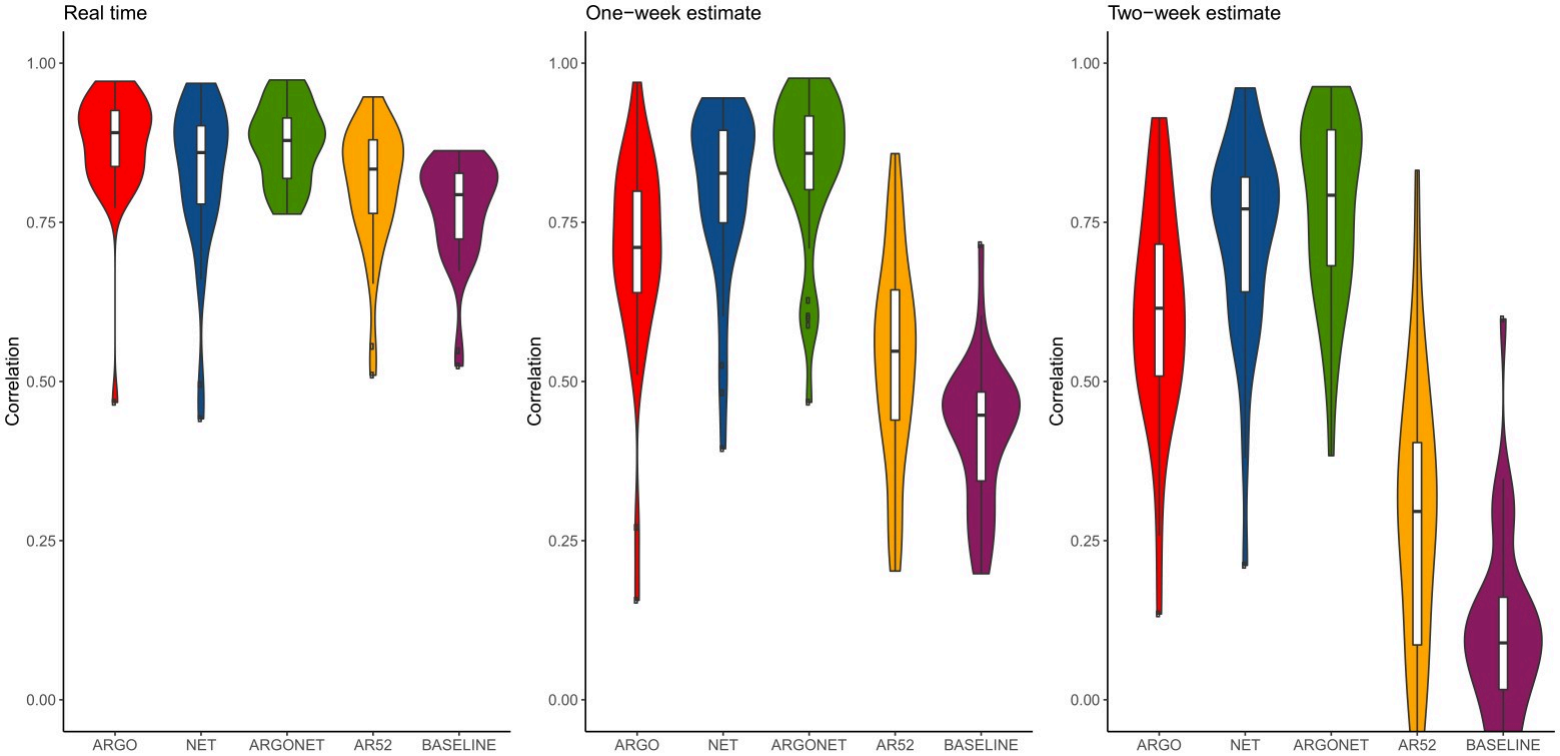
Coefficients Nouvelle-Aquitaine two-week ahead estimate.

Fig 14 shows two-week ahead estimates for the region Nouvelle-Aquitaine. As for one-week ahead estimate, we can see that estimates obtained with AR(52) is still delayed. It is not the case for ARGONet model. On the heatmap, Fig 15, we can see that ARGO model uses mostly nine variables, including six variables from Google Data, one variable from Climatic Data, two variables from Historical data.

## Discussion

We have introduced a machine learning ensemble methodology that combines multiple data sources and multiple statistical approaches to accurately track flu activity in the twelve continental regions of France. To the best of our knowledge, this is a spatial resolution for which no forecasting approaches have been explored before in France. Our methodology provides real-time estimates as well as one- and two-week ahead forecasts.

The success of our approach comes from the ability to dynamically identify the appropriate method and data sources to produce the best disease activity estimates for a given location and time horizon in a prospective way (out-of-sample). Specifically, we show that the ARGO model alone (one that does not incorporate flu activity from neighboring regions) yields accurate results for real-time estimates but fails to produce optimal predictions for longer-term time-horizons. We find that the Net model (one that leverages information from neighboring regions alone) leads to reasonable flu predictions but tends to overestimate epidemic peaks. The proposed ensemble approach, named ARGONet (that combines information from both ARGO and the Net model), an extension of a model proposed in the USA [9], produces forecasts with the lowest errors and highest correlation as captured by Fig 1. Particularly, the most reliable longer-term forecasts are obtained with ARGONet’s method using the mean of estimates from ARGO and Net models. This machine-learning ensemble approach displays both accuracy and robustness to estimate ILI activity up to two-weeks ahead of time at the french regional level. Our methodological approach was inspired by Lu et al. [9] using ARGO, Net and ARGONet methods to track flu activity at state level in United States. However, Rangarajan et al. [40] have shown that potential improvements can be achieved in data-driven forecasting methods by exploiting sparsity structures in the predictors. Future studies could explore the efficacy of these techniques for flu prediction in France.

Prediction error reductions are observed when using ARGONet over its autoregressive counterpart (AR(52)) (up to 32% across regions) in real-time predictions. As the time-horizon of prediction increases, the improvements of predictions are more evident, leading to up to 60% error reductions when comparing ARGONet with AR(52), and up to 70% error reduction when comparing ARGONet with the baseline (Tables 12 and 14). S2 through S13 Figs in S1 File show these results graphically. As expected, autoregressive approaches show “within-range” prediction values that consistently lag behind the observed disease activity and lead to under-predictions close to peak activity.

We find that all external data sources contribute to improving local flu estimates, when compared to the autoregressive model (AR(52)), specially for longer-term forecasts. Indeed, for the two-week ahead estimates, the combination of EHR data and Google data lead to correlation improvements of up to 30% and decreases in error of up to 25%. For Climatic data, this improvement reaches 20% for correlation and 11% for the error. For Twitter data, it reaches 20% for correlation and 7% for the error. By analyzing heatmaps (S5, S9 and S13 Figs in S1 File) obtained for ARGO models, we can see that the contribution of different predictors (data sources) changes over time and time-horizon of prediction, but all data sources appear to posses predictive power. Indeed, the most important data sources are EHR data and Google data in real-time and for longer-term forecasts. Historical data is consistently used in real-time, but less used for longer-term forecasting. Conversely, Climatic data and Twitter data are used more prominently for longer-term forecasts than for real-time estimate.

The fact that we could only access EHR data from Rennes University Hospital, and thus from the Brittany region, prevented us from being able to quantify the added valued of region-specific EHR information on flu predictions in their respective region. This should be evaluated in future research efforts. On the other hand, we find interesting the fact that data from a hospital in Rennes can improve flu forecasting in other regions. Indeed, S4-S6 Tables in S1 File show that forecasts that include EHR information from Rennes, up to two weeks, are more accurate for all the regions when compared to the autoregressive model (AR(52)). EHR data appears to be more relevant for some regions than others. For example, it appears to be an important predictor in the Brittany region (which contains Rennes) as expected, as well as in Normandy, which shares a border with Brittany. For Occitanie, EHR data from Rennes improves predictions, which is in alignment with the fact that historical information shows that flu activity tends to occur synchronously (with a correlation of 0.93) as seen in S1 Fig in S1 File. We hypothesize that having access to region-specific EHR data, from all the french regions, will lead to prediction improvements across the board.

Twitter data was collected at the National level given the sparsity of relevant flu-related Tweets at the regional level. This was the case as we only had access to the publicly available data shared by Twitter’s API that only allows users to view up to 5% of all Geo-coded Tweets (themselves a small fraction of about 5% of the total corpus of all Tweets). We also suspect that gaining access to higher volumes of Tweets at the regional level could improve our forecasts.

For climatic data, we only had a access to weekly local temperature and precipitation. Future studies may explore incorporating other climatic indicators known to be more directly related to the transmission of the virus, such as humidity [32].

For historical data, the variables with highest predictive power include lags or 52 weeks (one year). However, some other long-term lags show up as important in predictions (as 42 and 43 lags). Given the short time period of our study, we suspect that the flu seasons that we studied may have had specific trends (an early season combined with a late season) that could cause our methods to identify a meaningful influences of lags that are shorter than the intuitive 52 week lag.

Data retrieved from Google Correlate is normalized by Google in a (frequently) distinct sample and over different time periods depending on the data request. This pre-normalization can affect our results, but as shown in [2] the process of dynamic training minimizes the impact of this instability.

Our methods were designed to produce point estimates and decision-makers who are potential end-users of the output of our models would benefit from a quantification of the confidence we have in our predictions. For such purposes, by conducting a historical analysis on the errors between out-of-sample predictions and subsequent observations, we find that for a forward looking prediction, the bracket ($\hat{y_{t}}-RMSE,\hat{y_{t}}+RMSE$) can be thought of as a 95% confidence interval for each region at every point in time (See S80-S82 Figs in S1 File). This is consistent with previous work by Yang et al [2] where the collection of observed errors between (out-of-sample) predictions and subsequent observations were fitted using a Gaussian distribution (over a moving window of about 2 years, 104 observations) and the RMSE was found to be comparable to the standard deviation of such distribution (See S83-S85 Figs in S1 File). This is an empirical result and suggests that in 100 out-of- sample observations, 95 will fall within the suggested bracket around the point prediction. As shown in S80-S82 Figs in S1 File most observations fall within the proposed confidence intervals prospectively, confirming the validity of our approach.

To conclude, we have shown that Internet-based data sources can yield accurate influenza estimates in the twelve continental regions in France. Operational implementations of these methods may prove to be useful for public health officials in the face of public health threats. Our regional-level flu estimates may contribute to better management of patients’ flow in general practitioners’ offices and in hospitals, particularly emergency departments.

## Supporting information

S1 File (PDF)
