# Supplementary material for: Influenza forecasting for French regions combining EHR, web and climatic data sources with a machine learning ensemble approach
Source: PLoS One. 2021 May 19;16(5):e0250890. doi: 10.1371/journal.pone.0250890 (PMC8133501; doi:10.1371/journal.pone.0250890)
Supplement: S1 File — (PDF) [file pone.0250890.s001.pdf]

Influenza forecasting for French regions combining EHR, web and climatic data sources with a machine learning ensemble approach

Canelle Poirier [1,2,9,10*], Yulin Hswen [3,4], Guillaume Bouzillé [1,2,5], Marc Cuggia [1,2,5], Audrey Lavenu [6,7,8], John S Brownstein [4,9], Thomas Brewer [4], Mauricio Santillana [9,10*]

1 INSERM, U1099, Rennes, F-35000, France;
2 Université de Rennes 1, LTSI, Rennes, F-35000, France;
3 Department of Social and Behavioral Sciences, Harvard T.H. Chan School of Public Health, Boston, MA, USA
4 Innovation Program, Boston Children's Hospital, Boston, MA, USA
5 CHU Rennes, Centre de Données Cliniques, Rennes, F-35000, France;
6 Université de Rennes 1, Faculté de médecine, Rennes, F-35043, France;
7 INSERM CIC 1414, Université de Rennes 1, Rennes, F-35043, France;
8 IRMAR, Institut de Recherche Mathématique de Rennes, UMR CNRS 6625, Rennes, France;
9 Department of Pediatrics, Harvard Medical School, Boston, MA, USA;
10 Computational Health Informatics Program, Boston Children's Hospital, Boston, MA, USA;

* **Correspondence**: Canelle Poirier <canelle.poirier@outlook.fr> and Mauricio Santillana <msantill@g.harvard.edu>

List of legends

S1 Fig. Correlation between French regions on the period starting from January 2013 to March 2017

S2 Fig. Evolution of Auvergne-Rhˆone-Alpes estimates over time for AR(52) and ARGONET models

S3 Fig. Evolution of Bourgogne-Franche-Comte´ estimates over time for AR(52) and ARGONET models

S4 Fig. Evolution of Bretagne estimates over time for AR(52) and ARGONET models

S5 Fig. Evolution of Centre-Val de Loire estimates over time for AR(52) and ARGONET models

S6 Fig. Evolution of Grand Est estimates over time for AR(52) and ARGONET models

S7 Fig. Evolution of Hauts de France estimates over time for AR(52) and ARGONET models

S8 Fig. Evolution of Ile de France estimates over time for AR(52) and ARGONET models

S9 Fig. Evolution of Normandie estimates over time for AR(52) and ARGONet models

S10 Fig. Evolution of Nouvelle-Aquitaine estimates over time for AR(52) and ARGONet models

S11 Fig. Evolution of Occitanie estimates over time for AR(52) and ARGONet models

S12 Fig. Evolution of Pays de la Loire estimates over time for AR(52) and ARGONet models

S13 Fig. Evolution of Provence-Alpes-Côte d'Azur estimates over time for AR(52) and ARGONet models

S14 Fig. Auvergne Real-time estimate

S15 Fig. Coefficients Auvergne Real-time estimate

S16 Fig. Auvergne One-week estimate

S17 Fig. Coefficients Auvergne One-week estimate

S18 Fig. Auvergne Two-week estimate

S19 Fig. Coefficients Auvergne Two-week estimate

S20 Fig. Bourgogne Franche Comté Real-time estimate

S21 Fig. Coefficients Bourgogne Franche Comté Real-time estimate

S22 Fig. Bourgogne One-week estimate

S23 Fig. Coefficients Bourgogne Franche Comté One-week estimate

S24 Fig. Bourgogne Two-week estimate

S25 Fig. Coefficients Bourgogne Franche Comté Two-week estimate

S26 Fig. Bretagne Real-time estimate

S27 Fig. Coefficients Bretagne Real-time estimate

S1 Table. Real time estimate: RMSE and PCC for ARGO models including only historical data (AR(52)) and the 10 most correlated variables from Google data, for the period starting from January 2015 to March 2017

S2 Table. One-week ahead forecast : RMSE and PCC for ARGO models including only historical data (AR(52)) and the 10 most correlated variables from Google data, for the period starting from January 2015 to March 2017

S28 Fig. Bretagne One-week estimate

S29 Fig. Coefficients Bretagne One-week estimate

S30 Fig. Bretagne Two-week estimate

S31 Fig. Coefficients Bretagne Two-week estimate

S32 Fig. Centre Val-de-Loire Real-time estimate

S33 Fig. Coefficients Centre Val-de-Loire Real-time estimate

S34 Fig. Centre Val-de-Loire One-week estimate

S35 Fig. Coefficients Centre Val-de-Loire One-week estimate

S36 Fig. Centre Val-de-Loire Two-week estimate

S37 Fig. Coefficients Centre Val-de-Loire Two-week estimate

S38 Fig. Grand Est Real-time estimate

S39 Fig. Coefficients Grand Est Real-time estimate

S40 Fig. Grand Est One-week estimate

S41 Fig. Coefficients Grand Est One-week estimate

S42 Fig. Grand Est Two-week estimate

S43 Fig. Coefficients Grand Est Two-week estimate

S44 Fig. Hauts de France Real-time estimate

S45 Fig. Coefficients Hauts de France Real-time estimate

S46 Fig. Hauts de France One-week estimate

S47 Fig. Coefficients Hauts de France One-week estimate

S3 Table. Two-week ahead forecast : RMSE and PCC for ARGO models including only historical data (AR(52)) and the 10 most correlated variables from Google data, for the period starting from January 2015 to March 2017

S4 Table. Real time estimate: RMSE and PCC for ARGO models including only historical data (AR(52)) and the 10 most correlated variables from Hospital data, for the period starting from January 2015 to March 2017

S48 Fig. Hauts de France Two-week estimate

S5 Table. One-week ahead forecast: RMSE and PCC for ARGO models including only historical data (AR(52)) and the 10 most correlated variables from Hospital data, for the period starting from January 2015 to March 2017

S6 Table. Two-week ahead forecast: RMSE and PCC for ARGO models including only historical data (AR(52)) and the 10 most correlated variables from Hospital data, for the period starting from January 2015 to March 2017

S7 Table. PCC and RMSE for real-time estimate for all french regions for the period starting from January 2015 to March 2017 (only during the reporting period (week 40 to week 15))

S8 Table. PCC and RMSE for one-week ahead estimate for all french regions for the period starting from January 2015 to March 2017 (only during the reporting period (week 40 to week 15))

S9 Table. PCC and RMSE for two-week ahead estimate for all french regions for the period starting from January 2015 to March 2017 (only during the reporting period (week 40 to week 15))

S49 Fig. Coefficients Hauts de France Two-week estimate

S50 Fig. Ile de France Real-time estimate

S51 Fig. Coefficients Ile de France Real-time estimate

S52 Fig. Ile de France One-week estimate

S53 Fig. Coefficients Ile de France One-week estimate

S54 Fig. Ile de France Two-week estimate

S55 Fig. Coefficients Ile de France Two-week estimate

S56 Fig. Normandie Real-time estimate

S57 Fig. Coefficients Normandie Real-time estimate

S58 Fig. Normandie One-week estimate

S59 Fig. Coefficients Normandie One-week estimate

S60 Fig. Normandie Two-week estimate

S61 Fig. Coefficients Normandie Two-week estimate

S62 Fig. Occitanie Real-time estimate

S63 Fig. Coefficients Occitanie Real-time estimate

S64 Fig. Occitanie One-week estimate

S65 Fig. Coefficients Occitanie One-week estimate

S66 Fig. Occitanie Two-week estimate

S67 Fig. Coefficients Occitanie Two-week estimate

S68 Fig. Pays de la Loire Real-time estimate

S69 Fig. Coefficients Pays de la Loire Real-time estimate

S70 Fig. Pays de la Loire One-week estimate

S71 Fig. Coefficients Pays de la Loire One-week estimate

S72 Fig. Pays de la Loire Two-week estimate

S73 Fig. Coefficients Pays de la Loire Two-week estimate

S74 Fig. Provence Alpes Cˆote d'Azur Real-time estimate

S75 Fig. Coefficients Provence Alpes Cˆote d'Azur Real-time estimate

S76 Fig. Provence Alpes Cˆote d'Azur One-week estimate

S77 Fig. Coefficients Provence Alpes Cˆote d'Azur One-week estimate

S78 Fig. Provence Alpes Cˆote d'Azur Two-week estimate

S79 Fig. Coefficients Provence Alpes Cˆote d'Azur Two-week estimate

S80 Fig. ARGONet real-time estimates and its 95% confidence interval $[y\hat{}_t – RMSE; y\hat{}_t + RMSE]$

S81 Fig. ARGONet one-week estimates and its 95% confidence interval $[y\hat{}_t – RMSE; y\hat{}_t + RMSE]$

S82 Fig. ARGONet two-week estimates and its 95% confidence interval $[y\hat{}_t – RMSE; y\hat{}_t + RMSE]$

S83 Fig. Distribution of the residuals $(y_t - y\hat{}_t)$ for the real-time estimates obtained with ARGONet, showing that the RMSE is comparable to the standard deviation of the gaussian distribution.

S84 Fig. Distribution of the residuals $(y_t - y\hat{}_t)$ for the one-week estimates obtained with ARGONet, showing that the RMSE is comparable to the standard deviation of the gaussian distribution.

S85 Fig. Distribution of the residuals $(y_t - y\hat{}_t)$ for the two-week estimates obtained with ARGONet, showing that the RMSE is comparable to the standard deviation of the gaussian distribution.

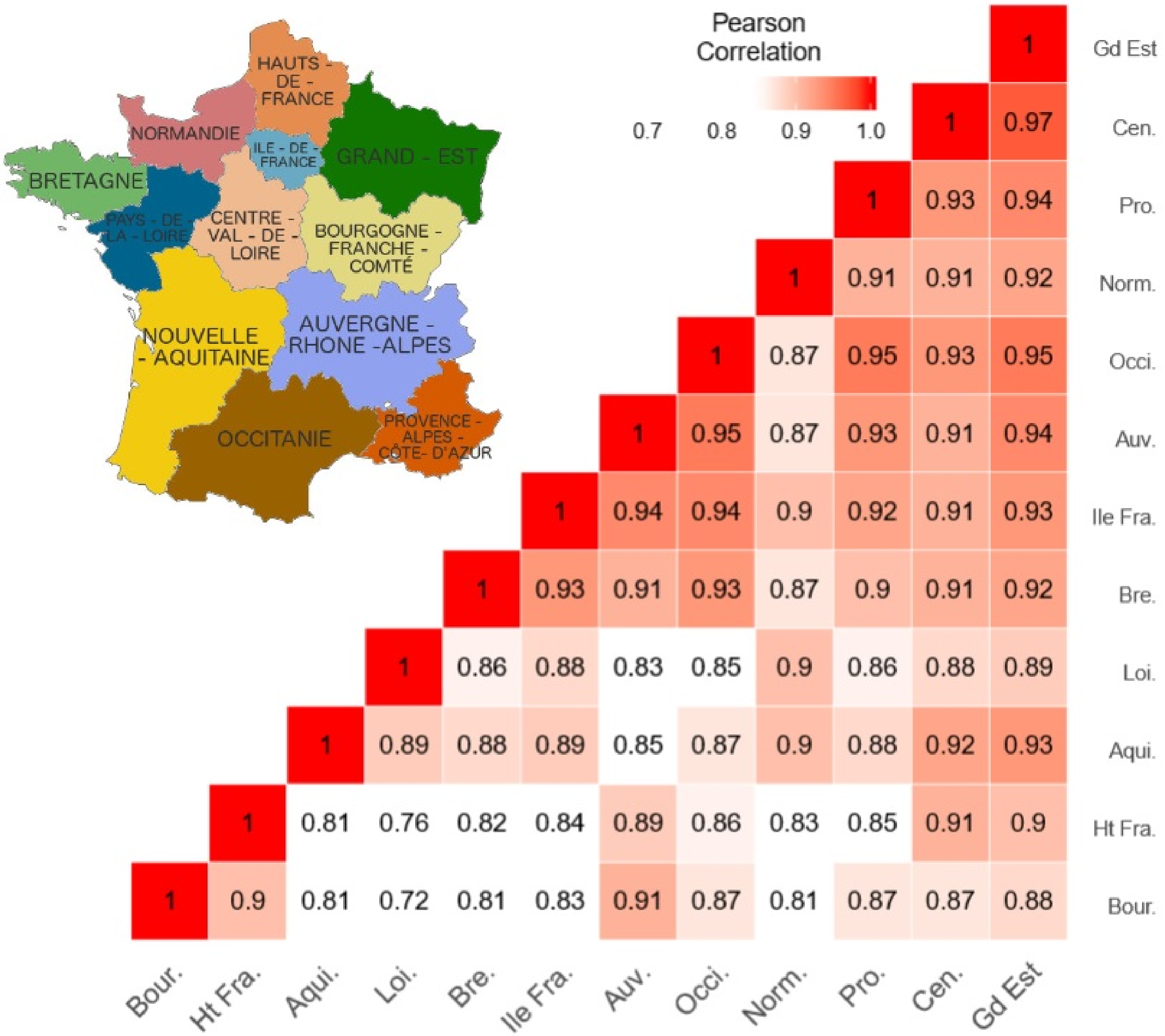

Correlation between French regions on the period starting from January 2013 to March 2017

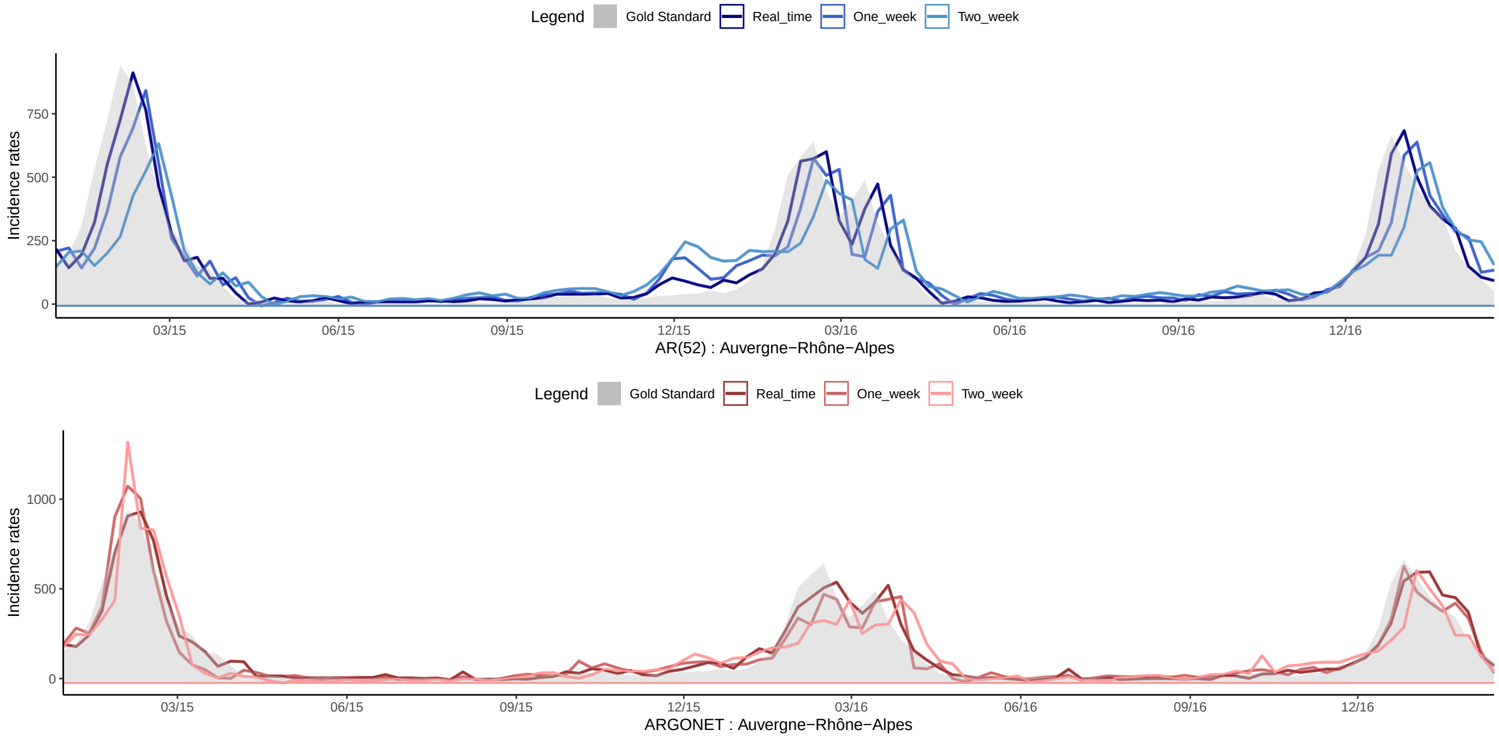

Evolution of Auvergne-Rhône-Alpes estimates over time for AR(52) and ARGONET models

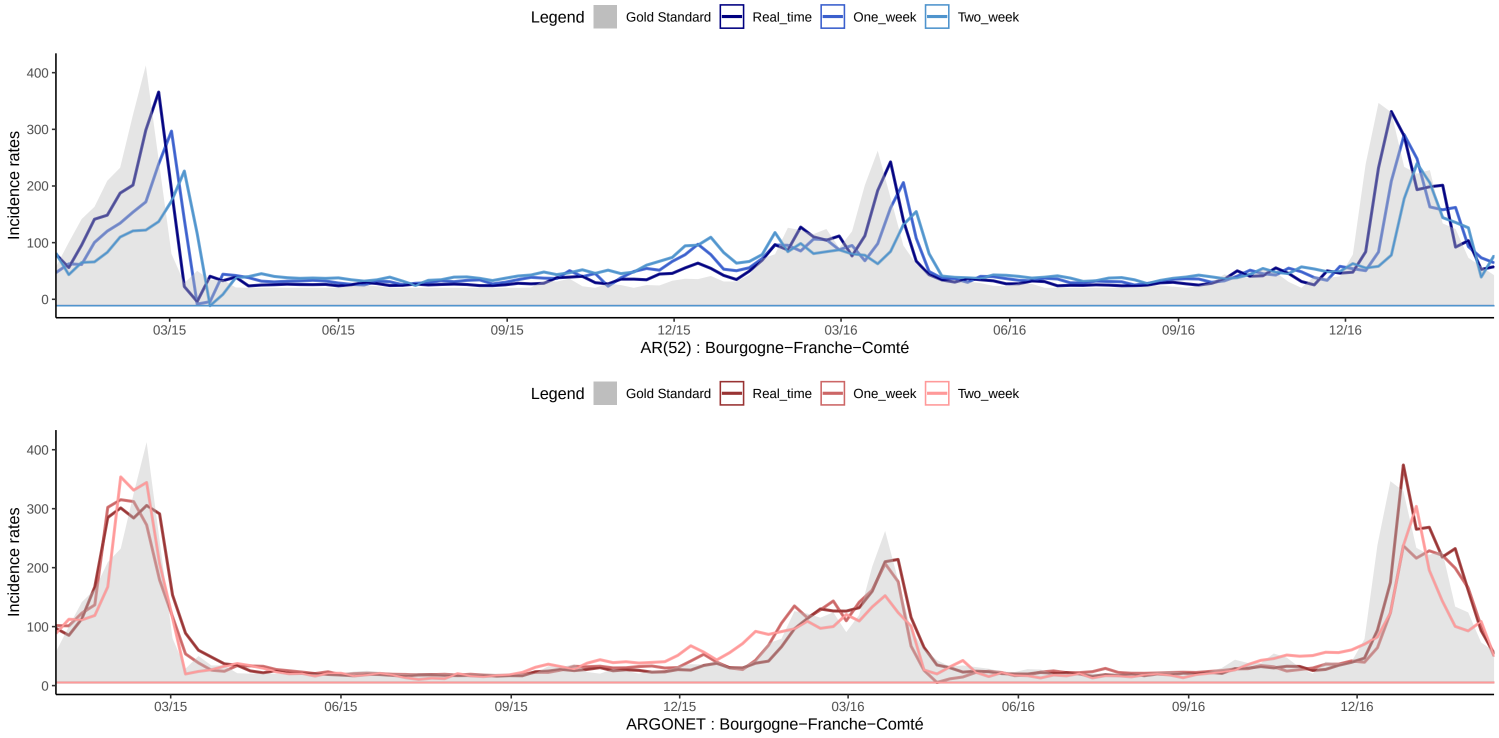

Evolution of Bourgogne-Franche-Comté estimates over time for AR(52) and ARGONET models

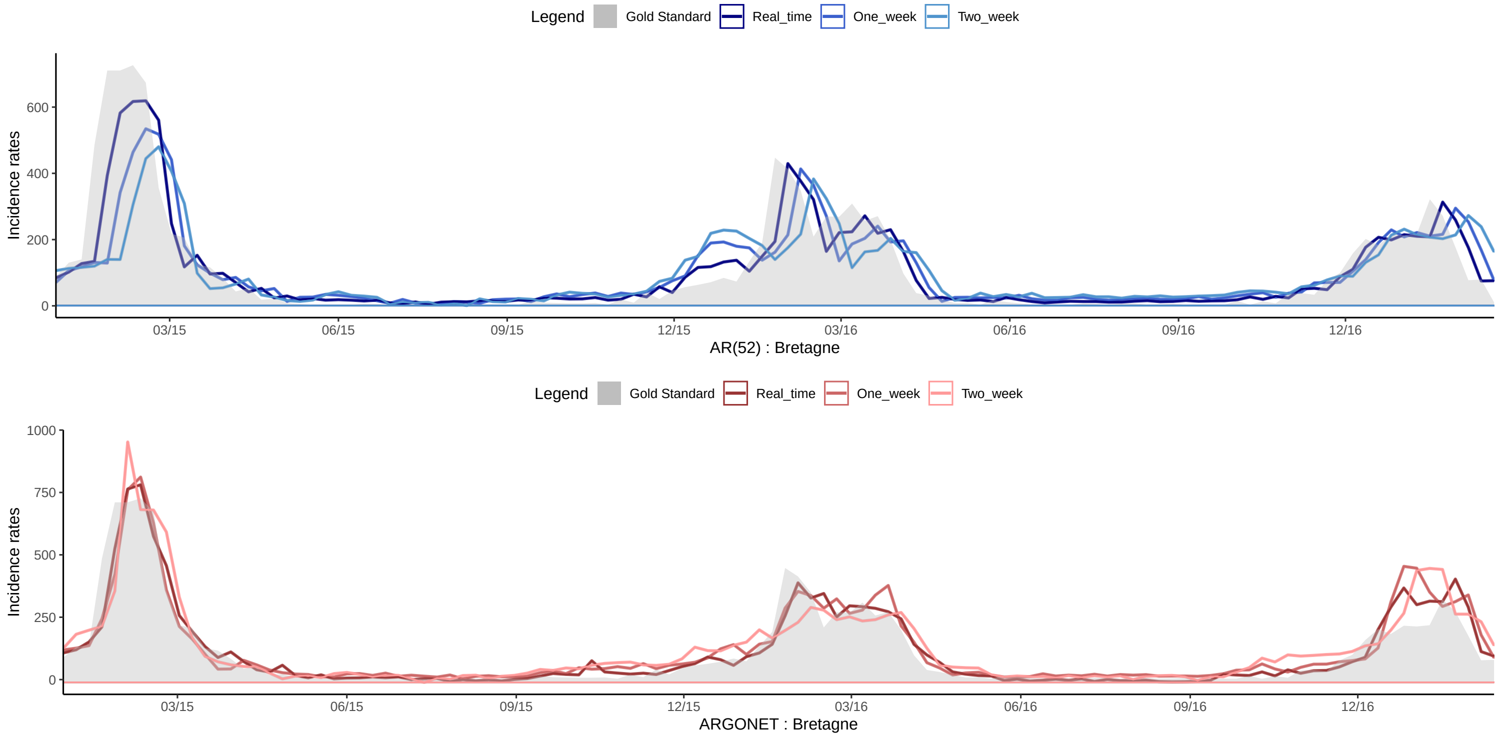

Evolution of Bretagne estimates over time for AR(52) and ARGONET models

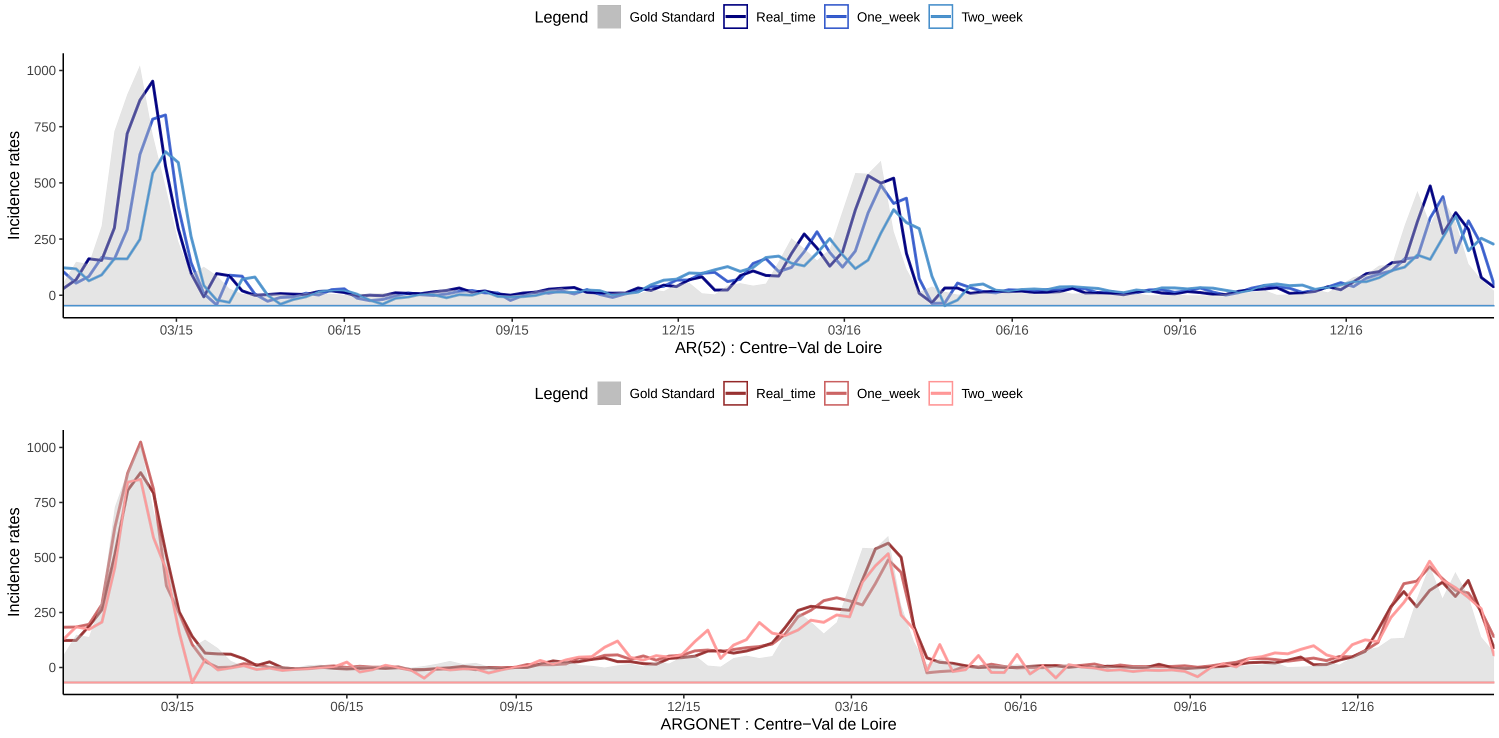

Evolution of Centre-Val de Loire estimates over time for AR(52) and ARGONET models

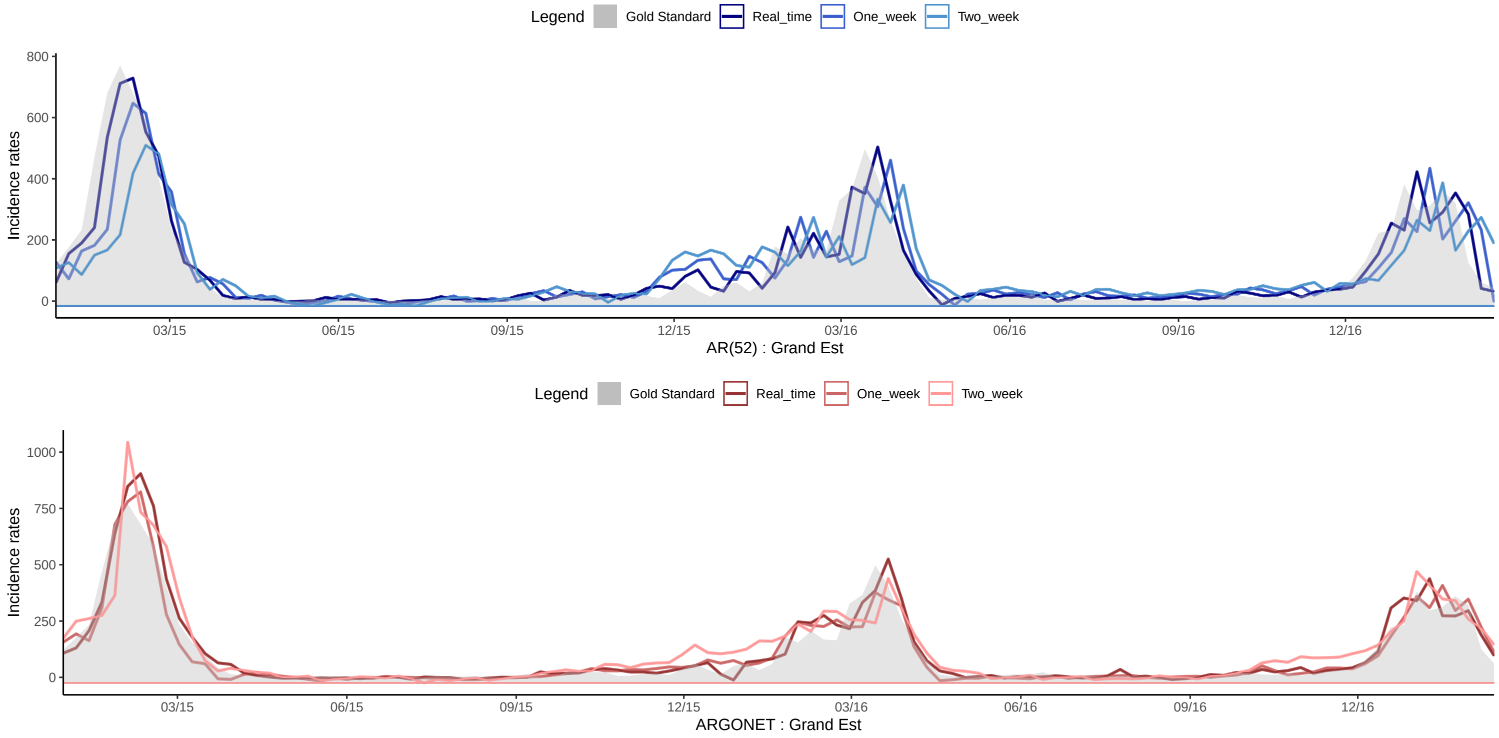

Evolution of Grand Est estimates over time for AR(52) and ARGONET models

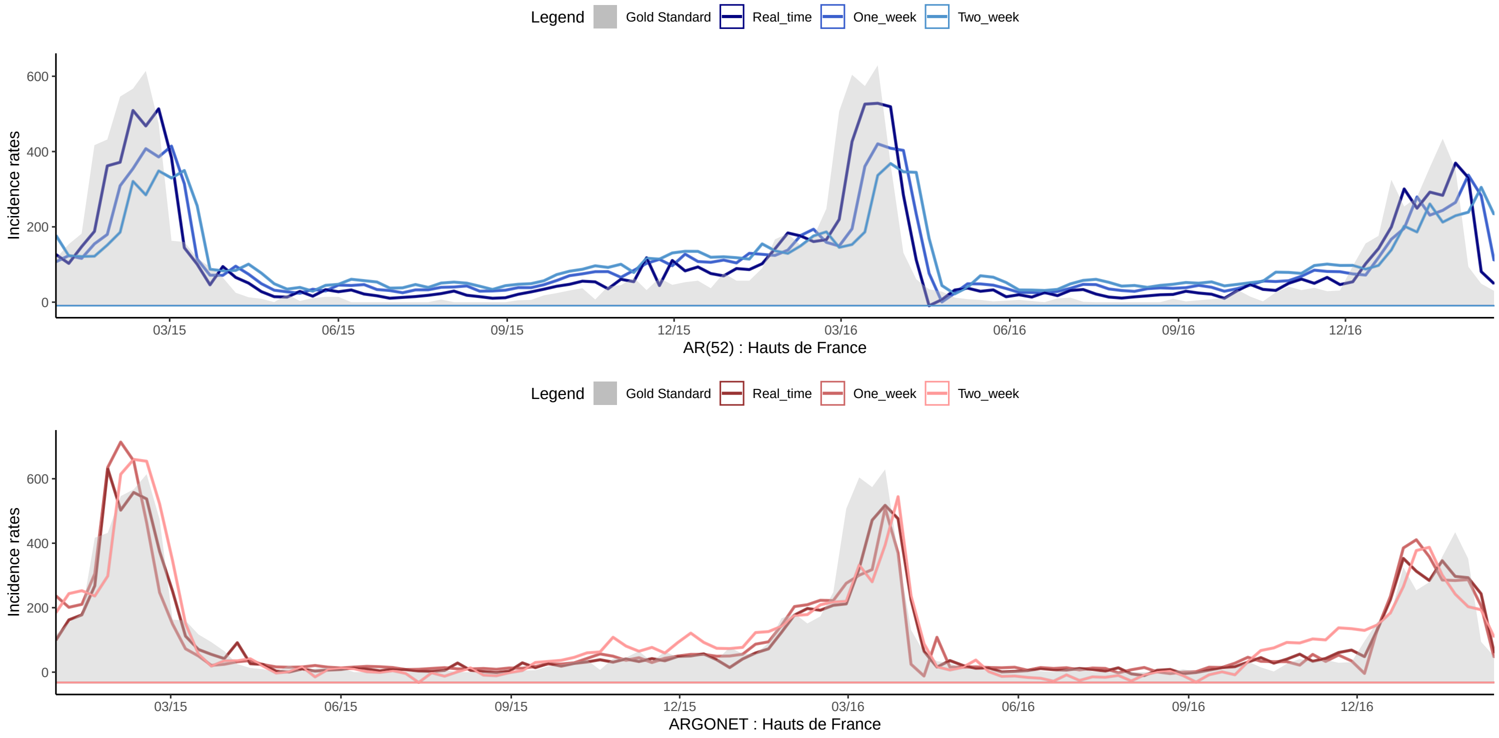

Evolution of Hauts de France estimates over time for AR(52) and ARGONET models

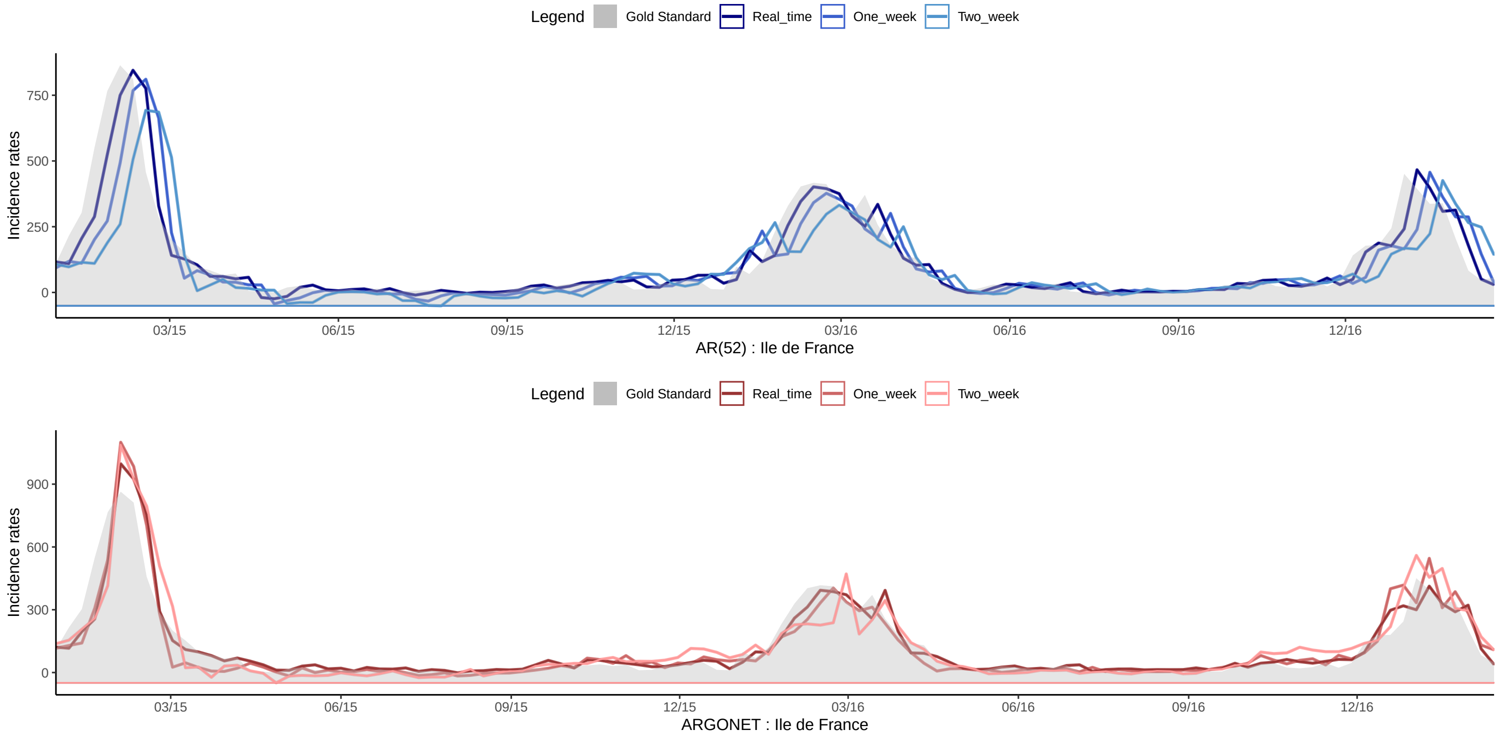

Evolution of Ile de France estimates over time for AR(52) and ARGONET models

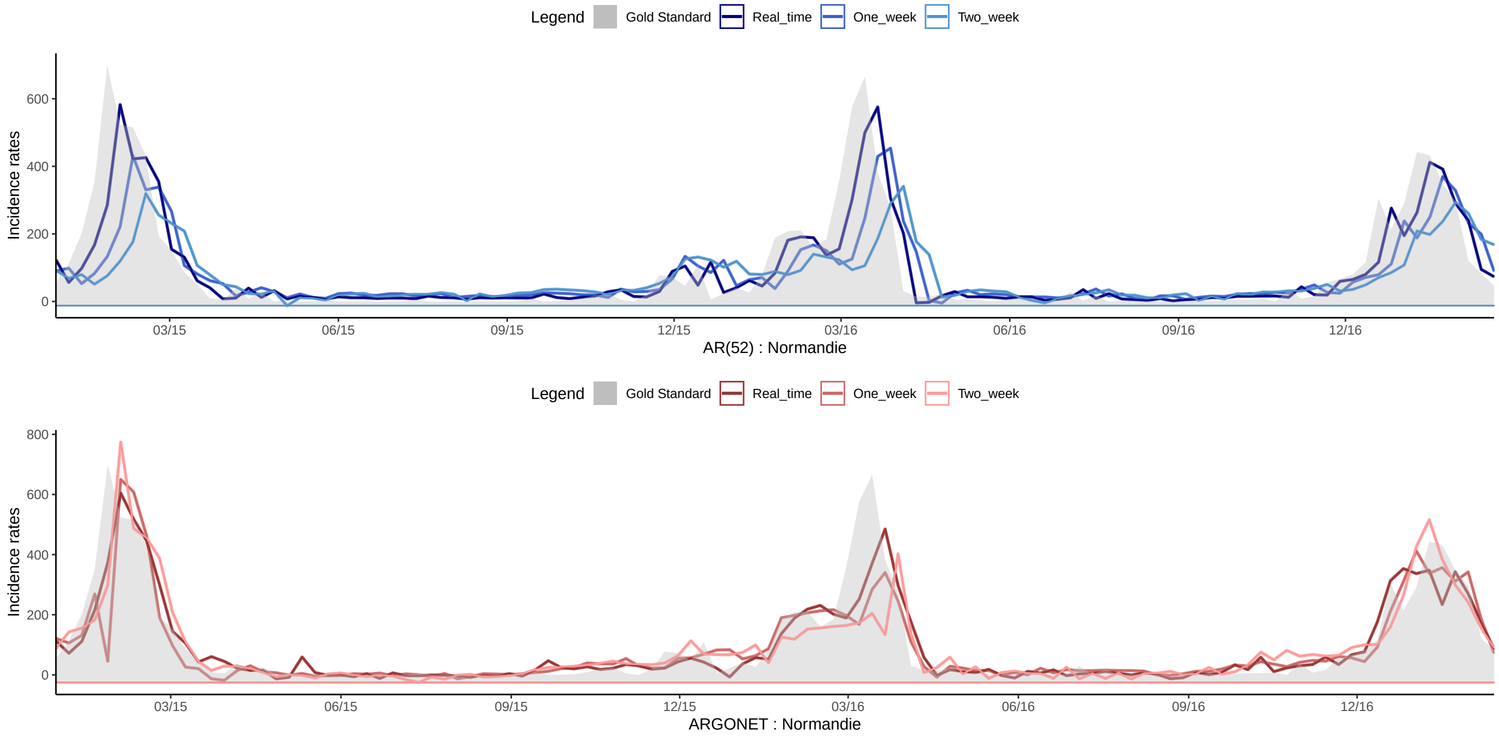

Evolution of Normandie estimates over time for AR(52) and ARGONet models

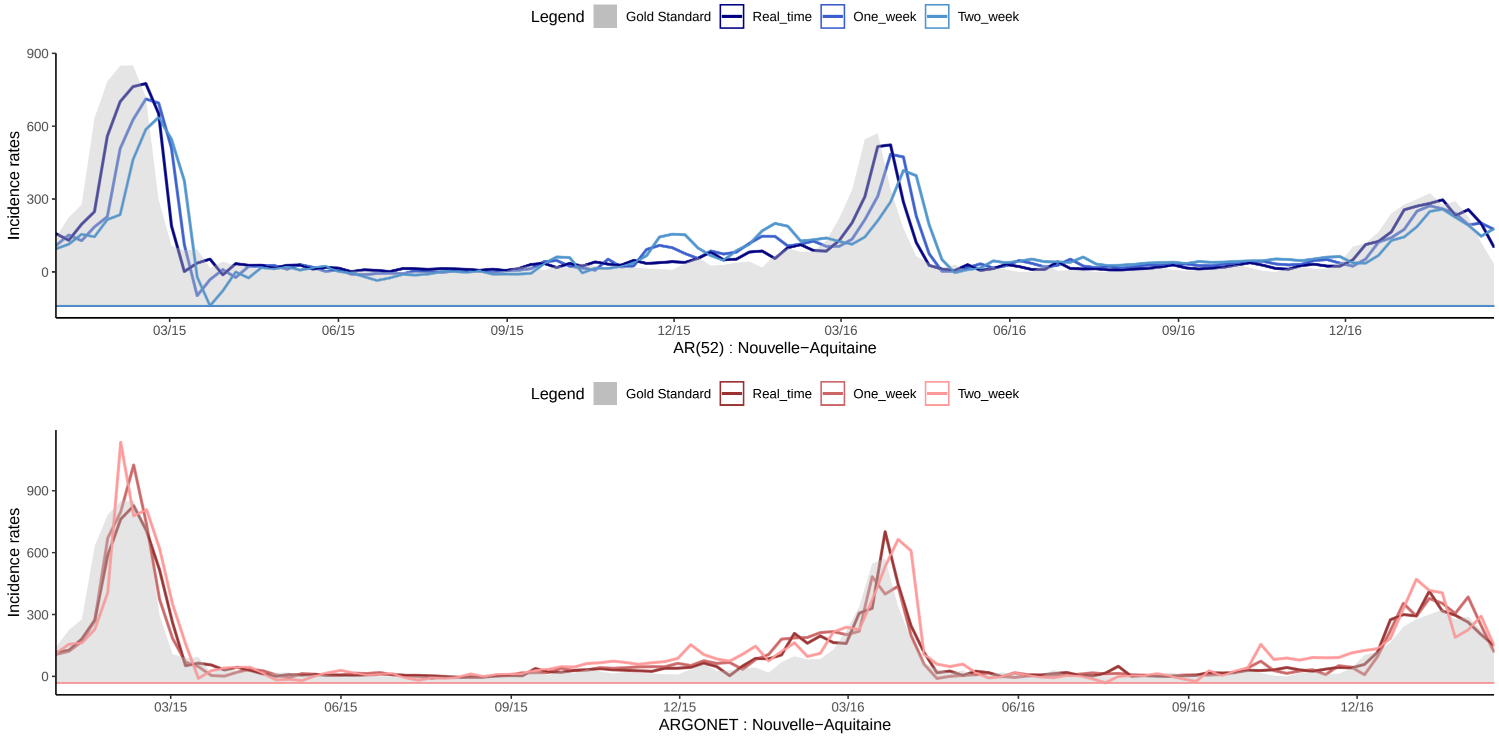

Evolution of Nouvelle-Aquitaine estimates over time for AR(52) and ARGONet models

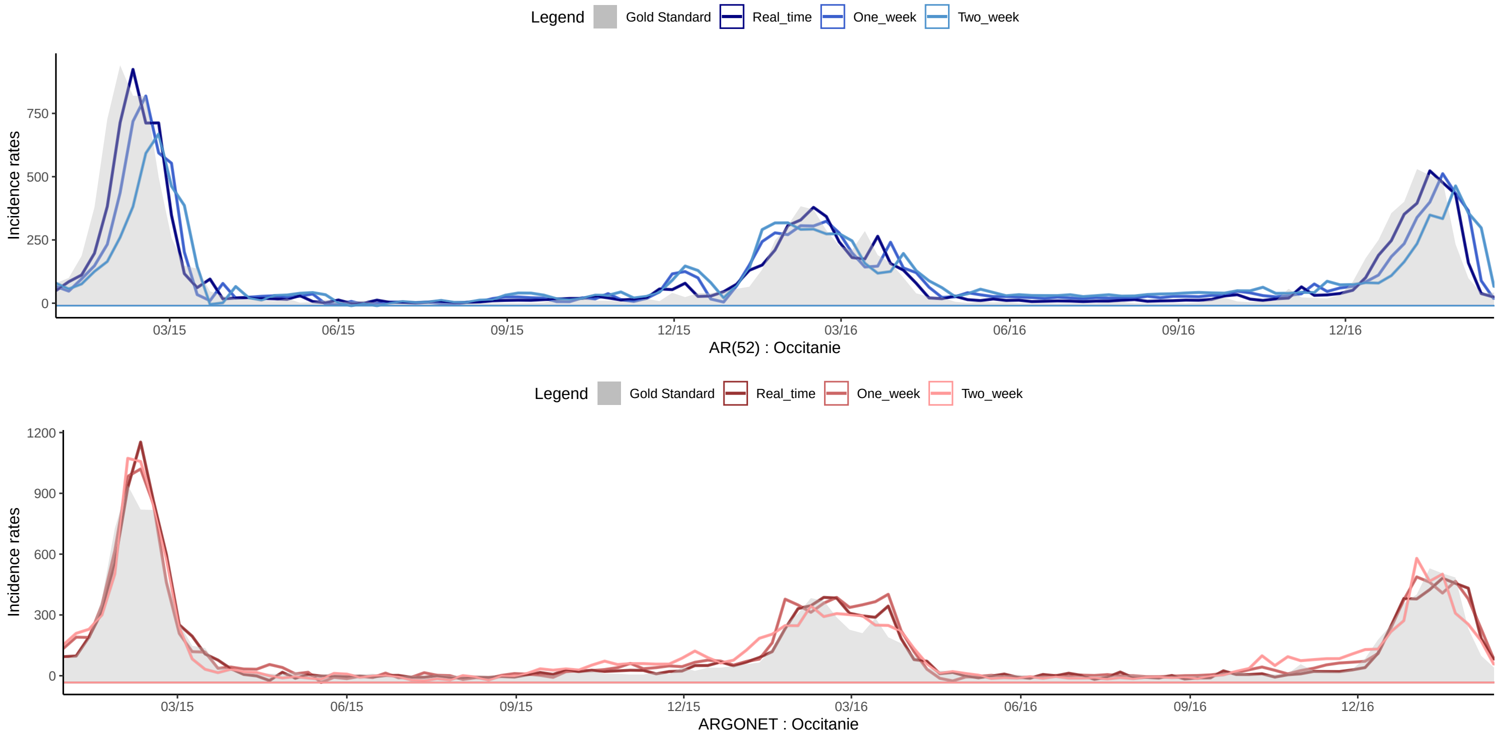

Evolution of Occitanie estimates over time for AR(52) and ARGONet models

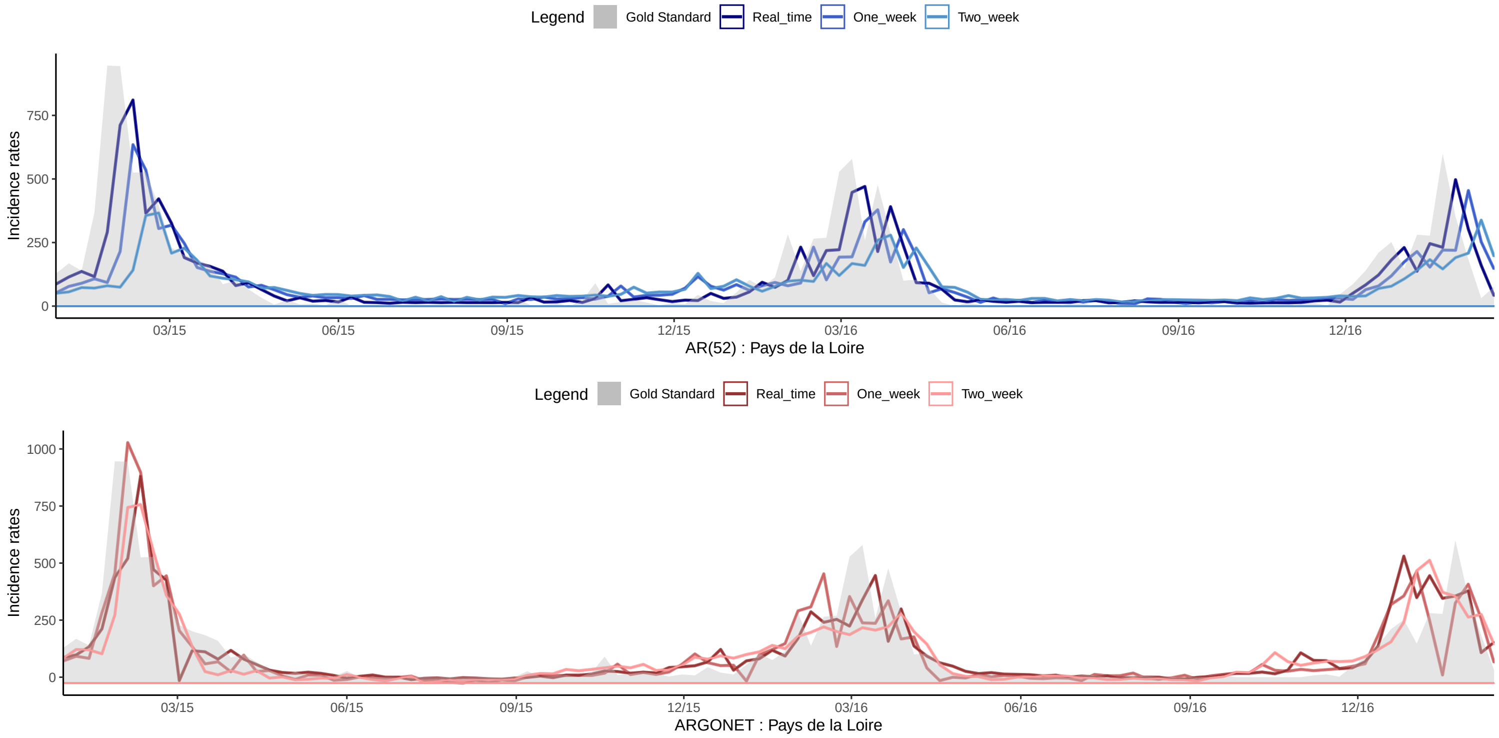

Evolution of Pays de la Loire estimates over time for AR(52) and ARGONet models

	Auv.	Bour.	Bre.	Cen.	Gd Est	Ht Fra.	Ile Fra.	Norm.	Aqui.	Occi.	Loi.	Pro.
**RMSE**												
AR(52)	**59.02**	36.24	65.41	77.00	**50.05**	**67.52**	61.34	75.29	71.55	58.48	102.38	82.49
ARGO	71.05	**35.12**	**60.72**	**64.49**	52.81	81.20	**56.41**	**65.58**	**64.40**	**55.22**	**100.67**	**66.28**
**PCC**												
AR(52)	0.958	0.898	0.916	0.919	0.952	0.915	0.935	0.879	0.919	0.946	0.815	0.929
ARGO	**0.967**	**0.929**	**0.941**	**0.959**	**0.967**	**0.935**	**0.949**	**0.911**	**0.944**	**0.976**	**0.839**	**0.964**

Real time estimate: RMSE and PCC for ARGO models including only historical data (AR(52)) and the 10 most correlated variables from Google data, for the period starting from January 2015 to March 2017

	Auv.	Bour.	Bre.	Cen.	Gd Est	Ht Fra.	Ile Fra.	Norm.	Aqui.	Occi.	Loi.	Pro.
**RMSE**												
AR(52)	**105.36**	59.41	99.54	122.54	**83.21**	103.89	101.55	110.63	117.00	102.94	139.49	134.43
ARGO	120.47	**56.61**	**80.86**	**97.55**	89.18	**96.80**	**94.98**	**92.36**	**106.34**	**93.04**	**132.67**	**99.73**
**PCC**												
AR(52)	0.868	0.692	0.782	0.779	0.861	0.782	0.813	0.707	0.758	0.860	0.645	0.804
ARGO	**0.890**	**0.832**	**0.884**	**0.903**	**0.913**	**0.875**	**0.847**	**0.812**	**0.835**	**0.924**	**0.713**	**0.914**

One-week ahead forecast : RMSE and PCC for ARGO models including only historical data (AR(52)) and the 10 most correlated variables from Google data, for the period starting from January 2015 to March 2017

	Auv.	Bour.	Bre.	Cen.	Gd Est	Ht Fra.	Ile Fra.	Norm.	Aqui.	Occi.	Loi.	Pro.
**RMSE**												
AR(52)	144.22	69.89	116.89	155.47	111.85	124.06	129.82	132.51	145.67	128.13	157.68	164.00
ARGO	**144.14**	**64.95**	**115.18**	**116.32**	**101.88**	**123.89**	**113.93**	**109.75**	**137.53**	**114.67**	**148.63**	**120.77**
**PCC**												
AR(52)	0.731	0.522	0.665	0.609	0.733	0.658	0.688	0.539	0.610	0.731	0.528	0.699
ARGO	**0.814**	**0.697**	**0.776**	**0.832**	**0.834**	**0.823**	**0.775**	**0.714**	**0.716**	**0.853**	**0.620**	**0.858**

Two-week ahead forecast : RMSE and PCC for ARGO models including only historical data (AR(52)) and the 10 most correlated variables from Google data, for the period starting from January 2015 to March 2017

	Auv.	Bour.	Bre.	Cen.	Gd Est	Ht Fra.	Ile Fra.	Norm.	Aqui.	Occi.	Loi.	Pro.
**RMSE**												
AR(52)	**59.02**	36.24	65.41	77.00	**50.05**	**67.52**	61.34	75.29	**71.55**	58.48	102.38	82.49
ARGO	60.65	**34.43**	**57.34**	**69.62**	52.27	72.99	**60.77**	**67.93**	72.44	**56.94**	**95.43**	**78.16**
**PCC**												
AR(52)	0.958	0.898	0.916	0.919	0.952	0.915	0.935	0.879	0.919	0.946	0.815	0.929
ARGO	**0.964**	**0.914**	**0.943**	**0.933**	**0.953**	**0.922**	**0.936**	**0.900**	**0.921**	**0.963**	**0.853**	**0.941**

Real time estimate: RMSE and PCC for ARGO models including only historical data (AR(52)) and the 10 most correlated variables from Hospital data, for the period starting from January 2015 to March 2017

	Auv.	Bour.	Bre.	Cen.	Gd Est	Ht Fra.	Ile Fra.	Norm.	Aqui.	Occi.	Loi.	Pro.
**RMSE**												
AR(52)	**105.36**	59.41	99.54	122.54	**83.21**	103.89	**101.55**	110.63	117.00	102.94	139.49	134.43
ARGO	105.93	**55.10**	**90.00**	**112.96**	91.38	**96.12**	101.72	**97.82**	**113.63**	**91.66**	**133.38**	**124.83**
**PCC**												
AR(52)	0.868	0.692	0.782	0.779	**0.861**	0.782	0.813	0.707	0.758	0.860	0.645	0.804
ARGO	**0.882**	**0.778**	**0.841**	**0.811**	0.857	**0.828**	**0.816**	**0.778**	**0.782**	**0.903**	**0.684**	**0.835**

One-week ahead forecast : RMSE and PCC for ARGO models including only historical data (AR(52)) and the 10 most correlated variables from Hospital data, for the period starting from January 2015 to March 2017

	Auv.	Bour.	Bre.	Cen.	Gd Est	Ht Fra.	Ile Fra.	Norm.	Aqui.	Occi.	Loi.	Pro.
**RMSE**												
AR(52)	144.22	69.89	116.89	155.47	**111.85**	124.06	129.82	132.51	145.67	128.13	157.68	164.00
ARGO	**141.39**	**64.98**	**107.81**	**144.14**	119.66	**107.47**	**129.14**	**118.83**	**142.21**	**123.51**	**142.78**	**147.46**
**PCC**												
AR(52)	0.731	0.522	0.665	0.609	0.733	0.658	**0.688**	0.539	0.610	0.731	0.528	0.699
ARGO	**0.754**	**0.590**	**0.732**	**0.681**	**0.687**	**0.741**	0.678	**0.668**	**0.632**	**0.772**	**0.618**	**0.757**

Two-week ahead forecast : RMSE and PCC for ARGO models including only historical data (AR(52)) and the 10 most correlated variables from Hospital data, for the period starting from January 2015 to March 2017

	Auv.	Bour.	Bre.	Cen.	Gd Est	Ht Fra.	Ile Fra.	Norm.	Aqui.	Occi.	Loi.	Pro.
**RMSE**												
Baseline	119.38	60.16	111.58	152.79	103.59	117.63	112.15	125.94	126.09	124.33	182.09	161.24
AR(52)	**94.84**	53.01	110.80	131.90	83.46	109.87	103.93	121.98	122.18	102.86	181.04	140.50
Argo	106.38	50.40	**88.37**	**93.44**	83.94	130.28	**92.46**	**99.72**	109.11	**83.00**	161.89	105.65
Net	105.55	45.00	113.54	104.63	76.80	**99.17**	106.66	129.19	108.50	96.83	178.21	109.76
K=1	98.83	49.75	100.74	97.52	83.89	102.55	96.26	105.68	**94.57**	85.29	170.06	102.68
K=2	97.54	48.34	99.74	105.62	77.56	107.73	94.73	109.39	**94.68**	83.19	166.66	99.47
K=3	97.91	47.90	97.73	107.02	**73.11**	105.28	96.45	121.77	101.39	83.85	**152.12**	106.23
K=4	106.59	48.04	104.63	102.04	78.55	119.71	98.15	**105.24**	101.30	**82.81**	**154.74**	108.07
Mean	99.98	**40.48**	93.59	**90.84**	73.26	100.90	95.89	107.64	104.08	79.24	155.64	**90.63**
Lm	**85.06**	**40.63**	**85.94**	93.94	**70.23**	**94.24**	**93.47**	130.02	108.02	74.18	168.16	**97.05**
**PCC**												
Baseline	0.871	0.795	0.824	0.816	0.862	0.811	0.852	0.786	0.864	0.864	0.661	0.821
AR(52)	0.917	0.824	0.824	0.861	0.910	0.829	0.874	0.793	0.869	0.905	0.630	0.863
Argo	0.947	**0.905**	**0.908**	**0.946**	0.940	0.874	**0.940**	**0.858**	0.906	**0.967**	0.732	0.931
Net	0.941	0.871	0.836	0.911	0.930	0.861	0.889	0.798	0.897	0.922	0.735	0.920
K=1	**0.948**	0.878	0.869	0.926	0.940	0.873	0.905	**0.854**	**0.923**	0.948	0.724	0.926
K=2	**0.948**	0.877	0.874	0.916	0.942	0.862	0.908	0.842	**0.923**	0.949	0.730	**0.937**
K=3	0.947	0.881	0.877	0.915	**0.943**	0.868	0.905	0.800	0.912	0.948	**0.779**	0.928
K=4	0.939	0.881	0.861	0.918	0.938	0.817	0.902	0.844	0.911	0.957	0.764	0.917
Mean	**0.950**	**0.907**	0.882	**0.938**	**0.943**	**0.886**	0.906	0.841	0.908	0.954	**0.769**	**0.943**
Lm	0.937	0.899	**0.899**	0.931	0.942	**0.874**	**0.910**	0.787	0.897	**0.964**	0.717	0.934

PCC and RMSE for real-time estimate for all french regions for the period starting from January 2015 to March 2017 (only during the reporting period (week 40 to week 15))

	Auv.	Bour.	Bre.	Cen.	Gd Est	Ht Fra.	Ile Fra.	Norm.	Aqui.	Occi.	Loi.	Pro.
**RMSE**												
Baseline	213.95	99.74	185.44	259.22	179.38	195.66	196.64	203.14	220.80	220.26	256.74	282.17
AR(52)	158.00	83.31	168.92	210.49	138.08	170.42	175.06	185.40	200.93	160.08	237.58	226.89
Argo	191.96	79.30	130.36	154.26	150.81	149.35	170.94	**152.96**	178.95	151.48	210.43	166.98
Net	161.26	50.85	112.45	138.33	119.14	126.33	133.75	189.80	125.68	149.69	228.42	143.90
K=1	152.03	59.02	123.41	143.91	121.93	140.26	**126.59**	183.30	129.60	142.80	**164.12**	149.65
K=2	182.55	66.82	127.09	149.09	137.35	150.82	165.72	186.53	142.18	142.62	206.64	139.38
K=3	185.55	61.00	121.31	154.60	134.43	150.74	149.78	187.72	152.04	137.66	**198.05**	166.40
K=4	187.85	54.23	108.72	170.01	137.53	151.63	146.12	187.50	132.83	138.15	205.66	177.72
Mean	**122.01**	**44.43**	**95.80**	**86.55**	**79.54**	**119.30**	**112.68**	**153.10**	**98.11**	**82.90**	**198.08**	**89.35**
Lm	**112.96**	**40.22**	**100.57**	**106.97**	**71.67**	**117.65**	133.45	179.12	**101.35**	**100.21**	223.26	**99.98**
**PCC**												
Baseline	0.578	0.469	0.508	0.476	0.587	0.470	0.546	0.437	0.583	0.569	0.331	0.452
AR(52)	0.750	0.516	0.518	0.613	0.737	0.522	0.628	0.453	0.613	0.753	0.290	0.607
Argo	0.807	0.817	0.744	0.842	0.818	0.784	0.748	**0.643**	0.724	0.864	0.549	0.839
Net	0.871	0.845	0.863	0.872	0.889	0.806	**0.921**	0.591	0.902	0.853	0.639	0.904
K=1	0.868	0.842	0.827	0.853	0.854	0.763	0.902	0.539	0.899	0.876	**0.741**	0.864
K=2	0.857	0.813	0.818	0.842	0.855	0.750	0.850	0.521	0.887	0.925	0.649	0.879
K=3	0.855	0.817	0.821	0.828	0.863	0.764	0.866	0.502	0.858	0.941	**0.678**	0.838
K=4	0.853	0.882	**0.871**	0.823	0.861	0.763	0.867	0.498	0.890	0.929	0.652	0.804
Mean	**0.912**	**0.907**	**0.868**	**0.940**	**0.940**	**0.828**	0.903	**0.671**	**0.916**	**0.951**	0.646	**0.948**
Lm	**0.908**	**0.897**	**0.868**	**0.913**	**0.937**	**0.820**	**0.914**	0.543	**0.920**	**0.944**	0.604	**0.941**

PCC and RMSE for one-week ahead estimate for all french regions for the period starting from January 2015 to March 2017(only during the reporting period (week 40 to week 15))

	Auv.	Bour.	Bre.	Cen.	Gd Est	Ht Fra.	Ile Fra.	Norm.	Aqui.	Occi.	Loi.	Pro.
**RMSE**												
Baseline	290.60	120.77	240.47	339.03	238.73	245.02	262.54	258.71	291.31	300.49	295.31	367.83
AR(52)	221.74	95.15	199.59	265.34	183.12	199.07	219.55	221.97	243.97	214.55	267.05	273.18
Argo	222.75	80.24	193.22	188.83	170.87	187.83	204.54	**180.70**	**226.71**	199.87	226.73	198.47
Net	215.92	47.45	169.50	136.23	174.70	161.90	197.90	191.75	245.09	89.80	**186.91**	170.21
K=1	203.26	59.53	**180.90**	**95.77**	176.09	161.58	192.96	190.52	243.56	**96.52**	209.78	172.93
K=2	229.94	57.63	195.04	**101.71**	185.42	158.32	201.88	196.06	244.90	104.74	209.84	176.72
K=3	222.50	57.78	190.76	120.39	196.40	179.73	**185.46**	203.32	256.79	98.70	189.64	173.00
K=4	222.83	58.20	198.87	129.61	201.92	178.82	191.13	191.63	261.78	106.74	190.37	208.12
Mean	**162.72**	**45.90**	**131.25**	133.94	**113.82**	**138.75**	**139.97**	**152.31**	**167.94**	106.39	**187.28**	**121.11**
Lm	**156.69**	**44.37**	204.40	129.73	**153.88**	**125.10**	215.13	200.98	296.20	**90.01**	201.61	**129.90**
**PCC**												
Baseline	0.220	0.235	0.185	0.122	0.278	0.177	0.201	0.084	0.281	0.205	0.138	0.082
AR(52)	0.438	0.319	0.258	0.304	0.487	0.273	0.363	0.098	0.386	0.504	0.048	0.383
Argo	0.672	0.687	0.491	0.712	0.667	0.709	0.593	0.467	0.548	0.692	0.446	0.733
Net	0.771	**0.875**	**0.765**	0.855	0.795	0.706	0.814	**0.655**	0.751	**0.932**	**0.661**	0.865
K=1	0.812	0.837	0.759	**0.937**	0.785	0.727	**0.845**	0.635	**0.776**	0.919	0.512	0.873
K=2	0.810	0.839	0.746	**0.922**	0.787	0.729	**0.844**	0.603	0.768	0.904	0.518	0.792
K=3	**0.814**	0.838	0.761	0.884	0.782	0.714	0.798	0.592	0.771	0.916	**0.658**	0.798
K=4	0.811	0.833	0.746	0.866	0.786	0.717	0.825	0.631	0.770	0.901	0.648	0.825
Mean	**0.816**	0.872	**0.764**	0.849	**0.852**	**0.783**	0.825	**0.671**	**0.782**	0.900	0.615	**0.899**
Lm	0.813	**0.881**	0.709	0.867	**0.811**	**0.774**	0.831	0.603	0.715	**0.938**	0.633	**0.892**

PCC and RMSE for two-week ahead estimate for all french regions for the period starting from January 2015 to March 2017(week 40 to week 15))

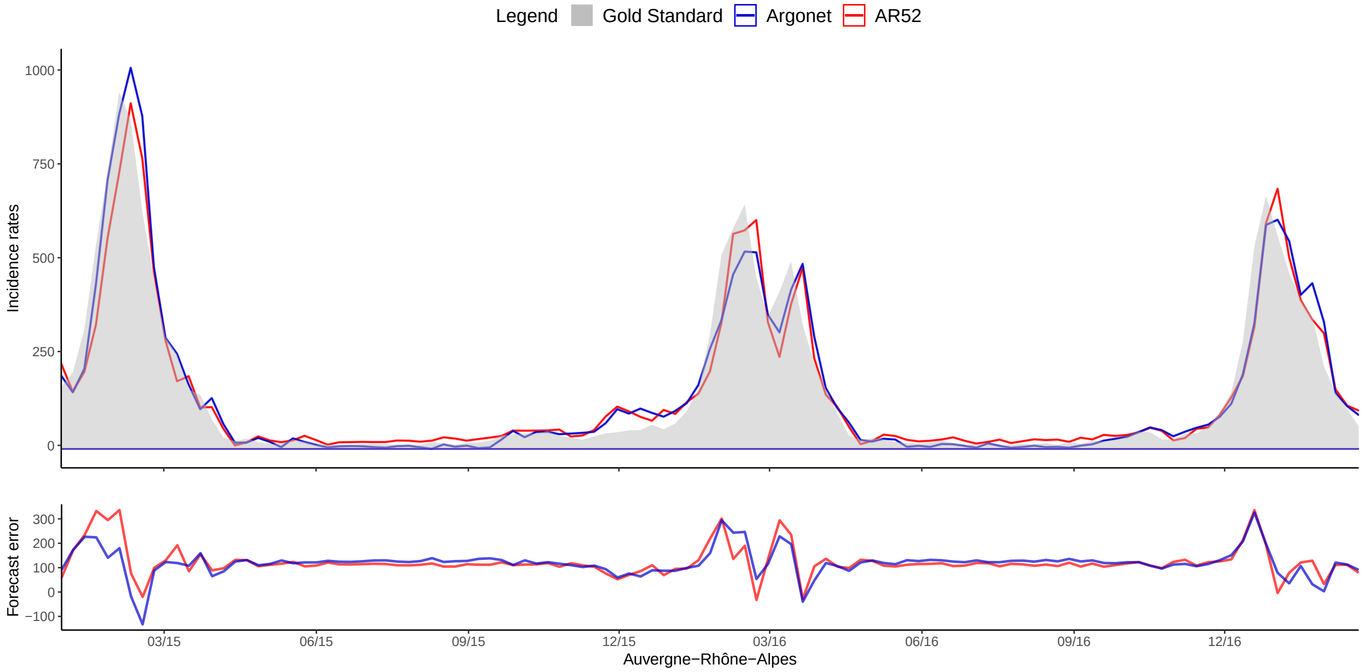

Auvergne Real-time estimate

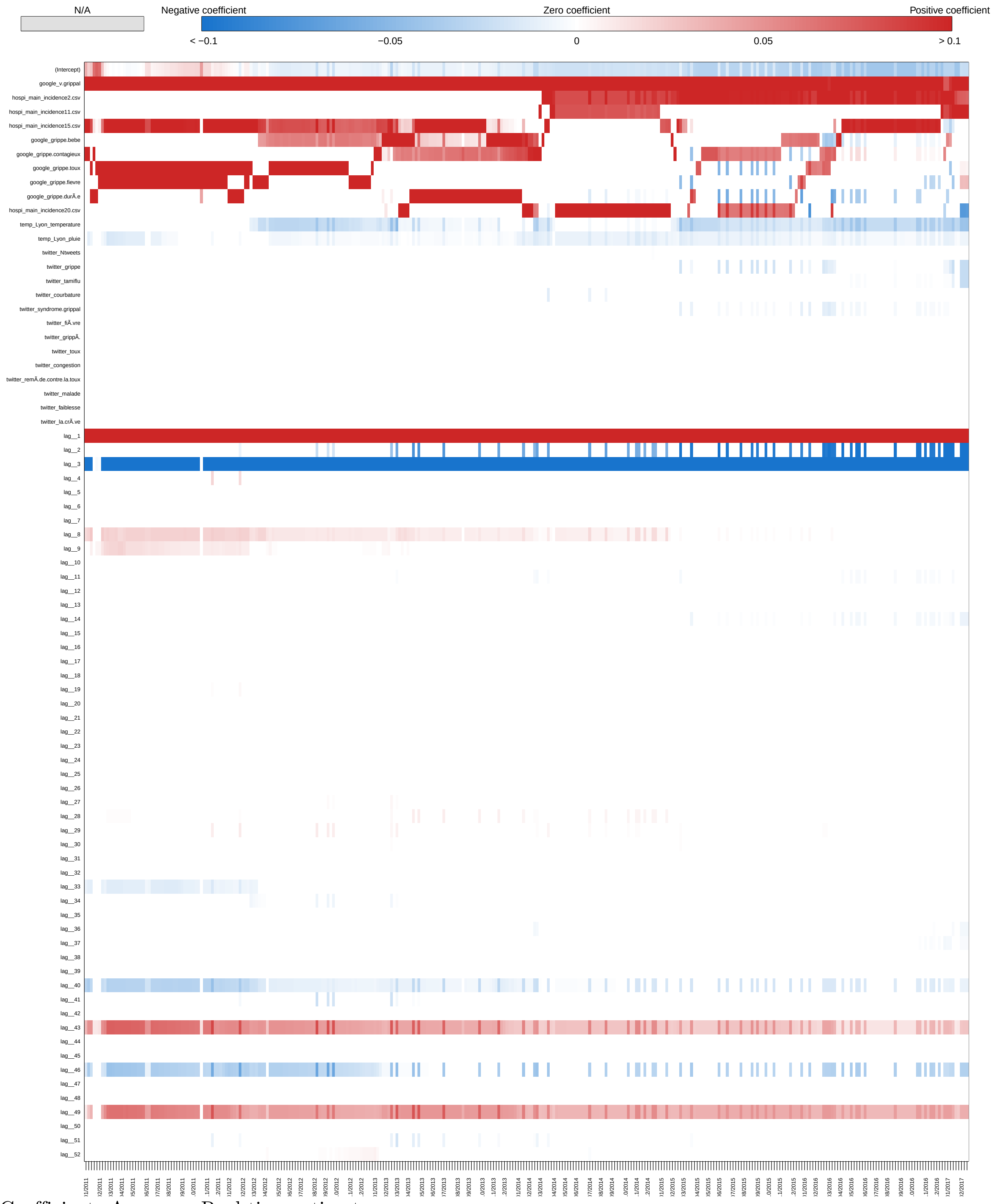

Coefficients Auvergne Real-time estimate

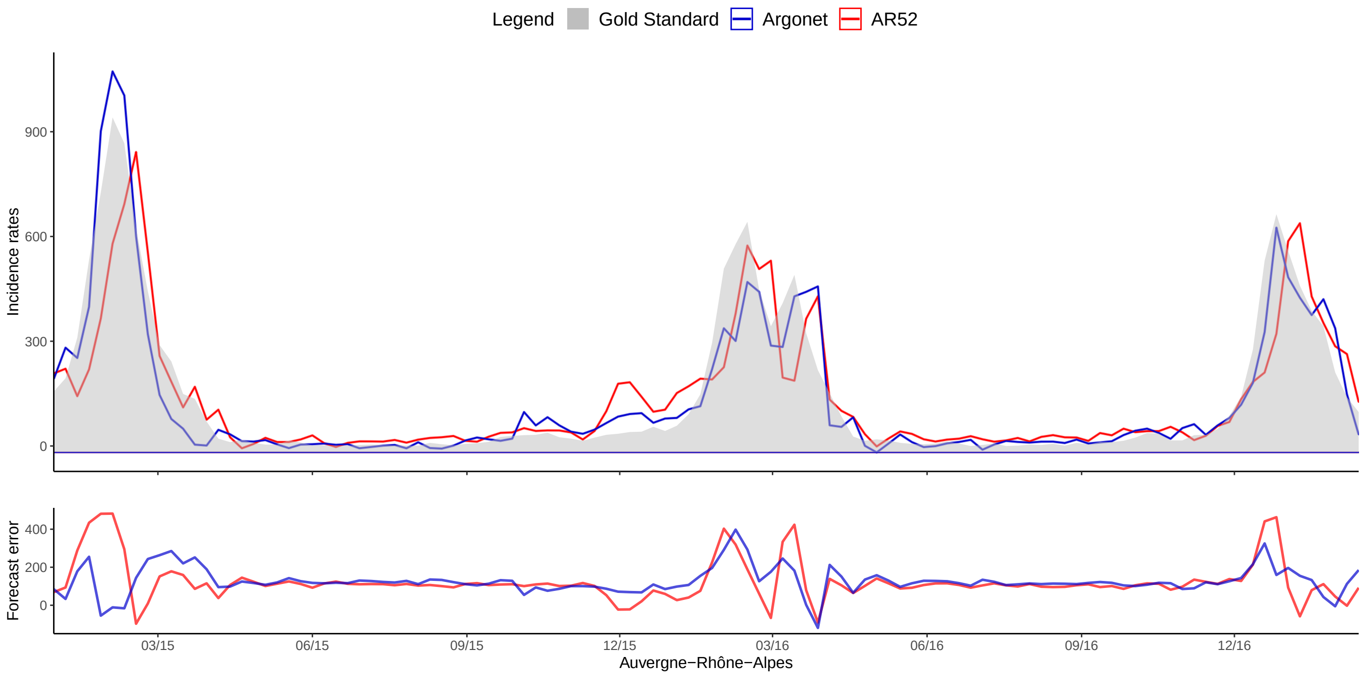

Auvergne One-week estimate

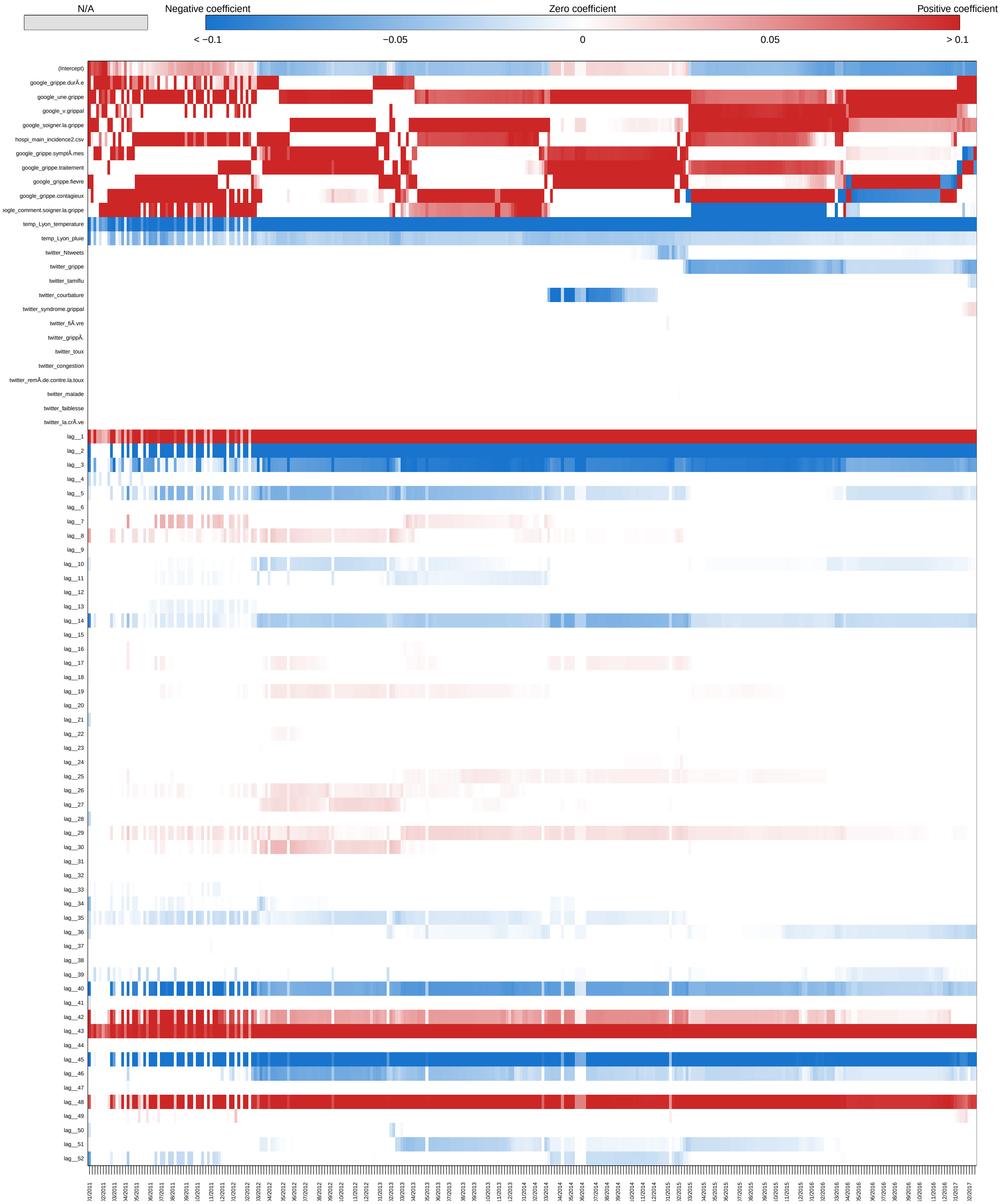

Coefficients Auvergne One-week estimate

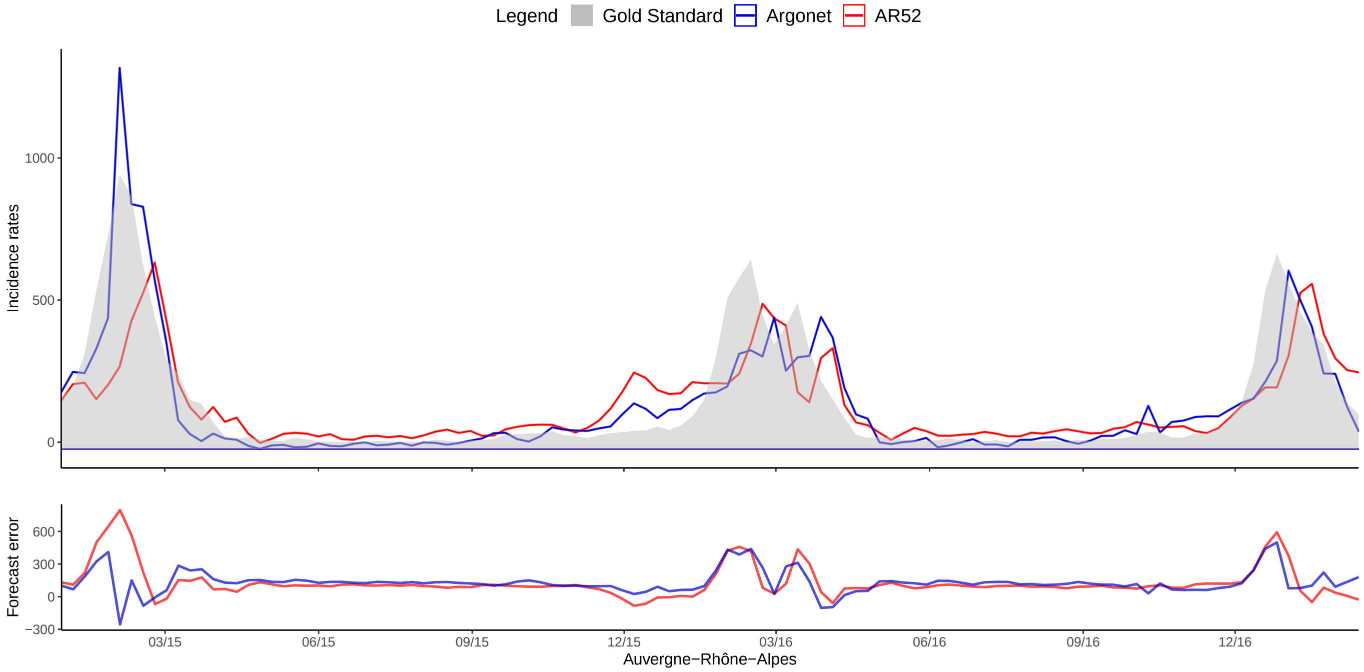

Auvergne Two-week estimate

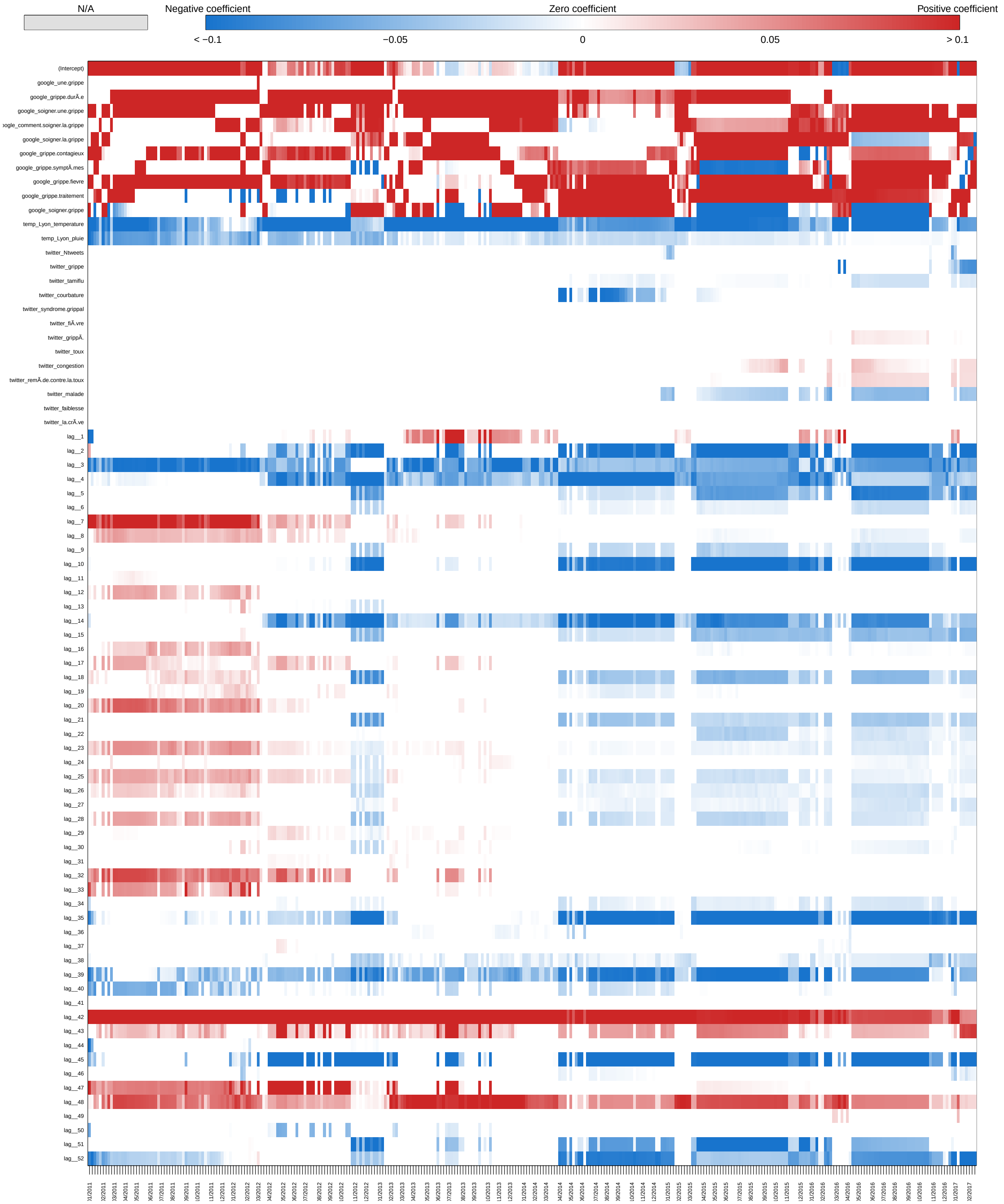

Coefficients Auvergne Two-week estimate

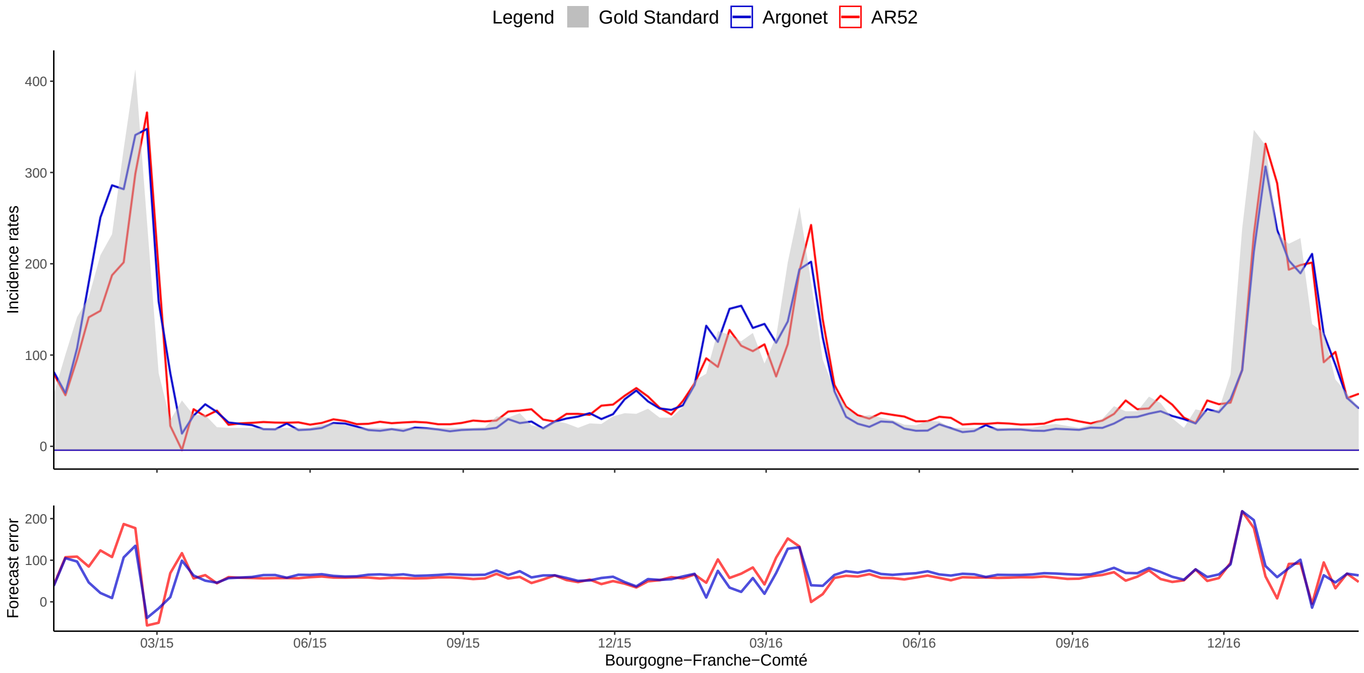

Bourgogne Franche Comté Real-time estimate

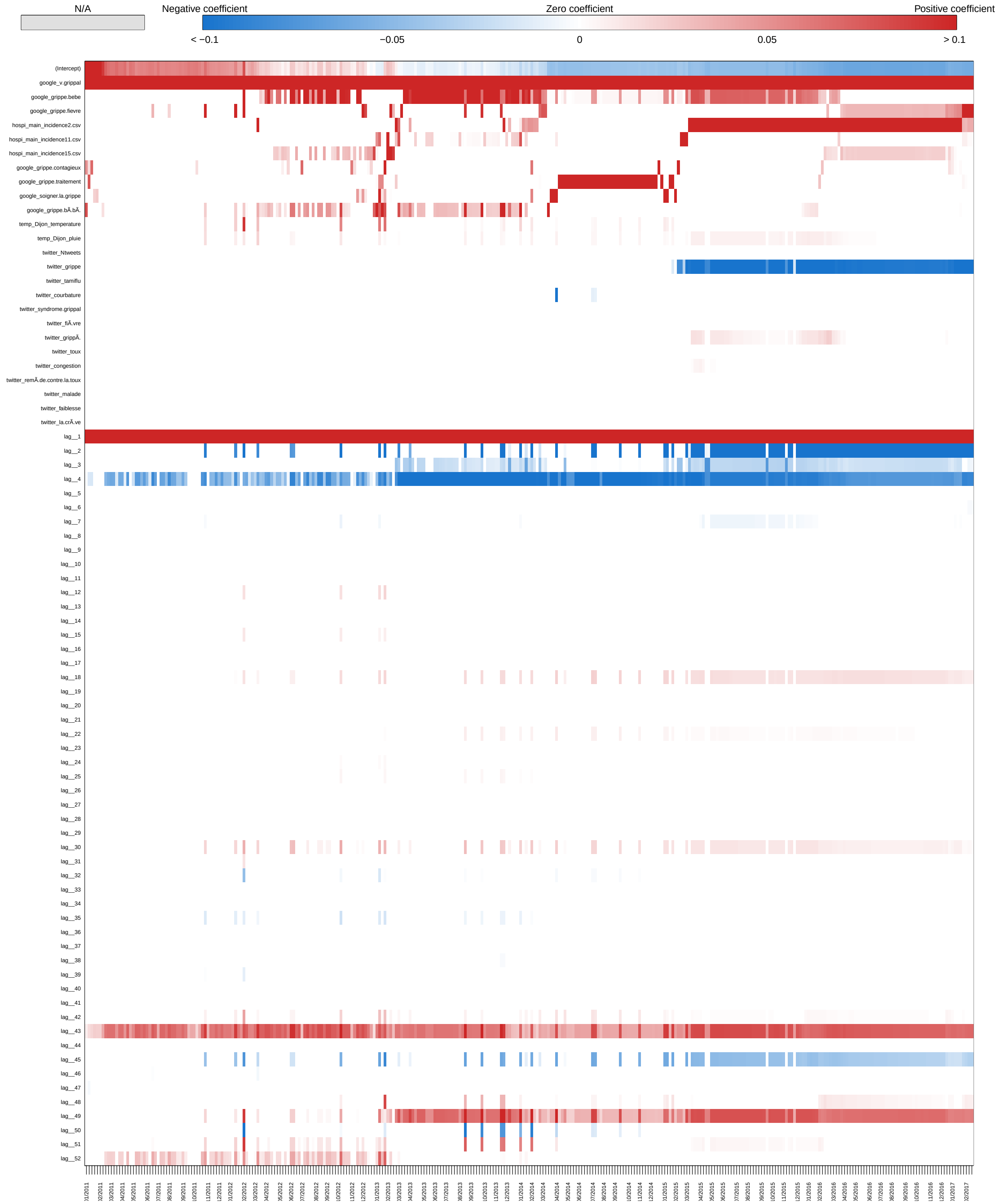

Coefficients Bourgogne Franche Comté Real-time estimate

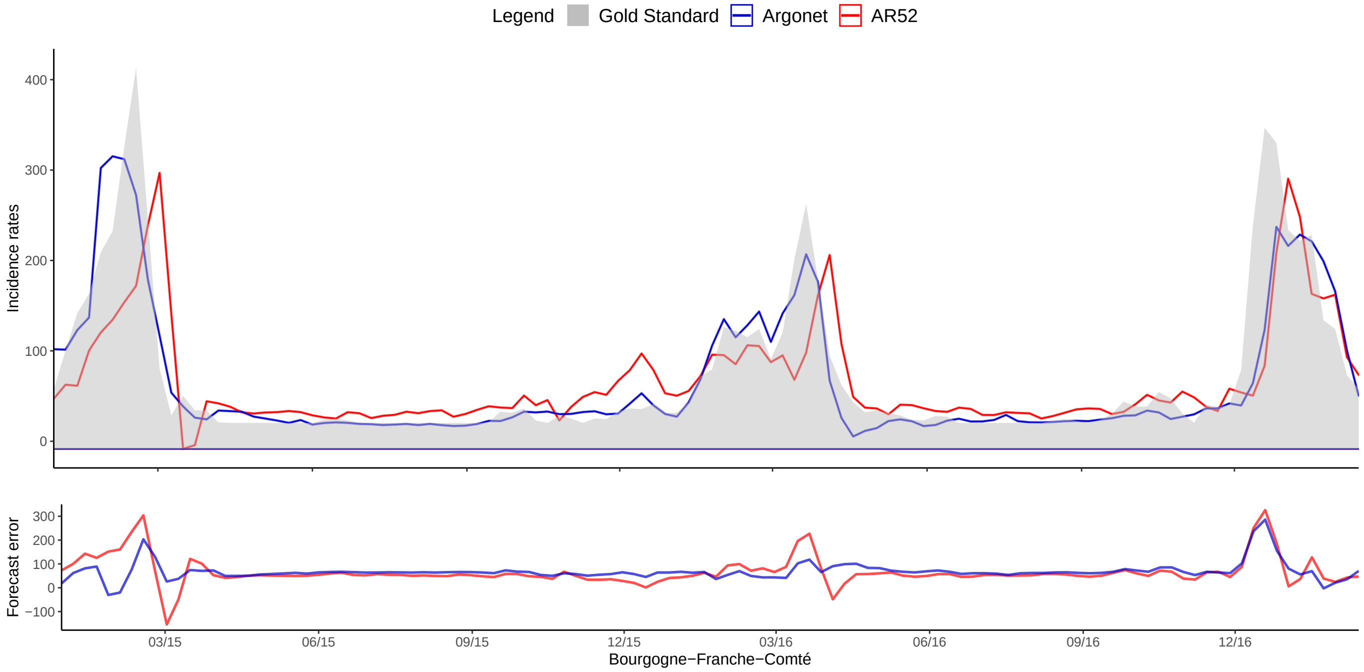

Bourgogne One-week estimate

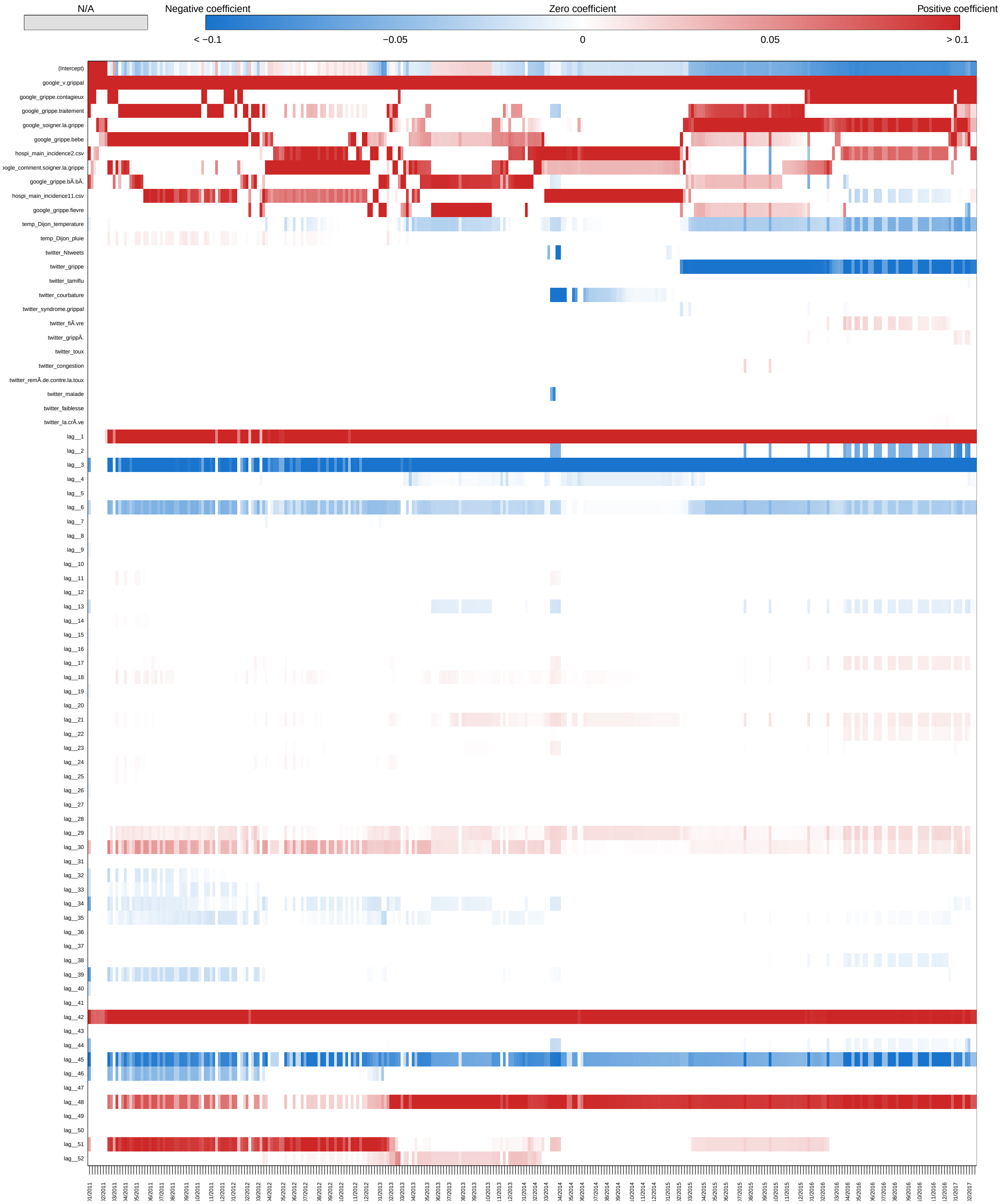

Coefficients Bourgogne Franche Comté One-week estimate

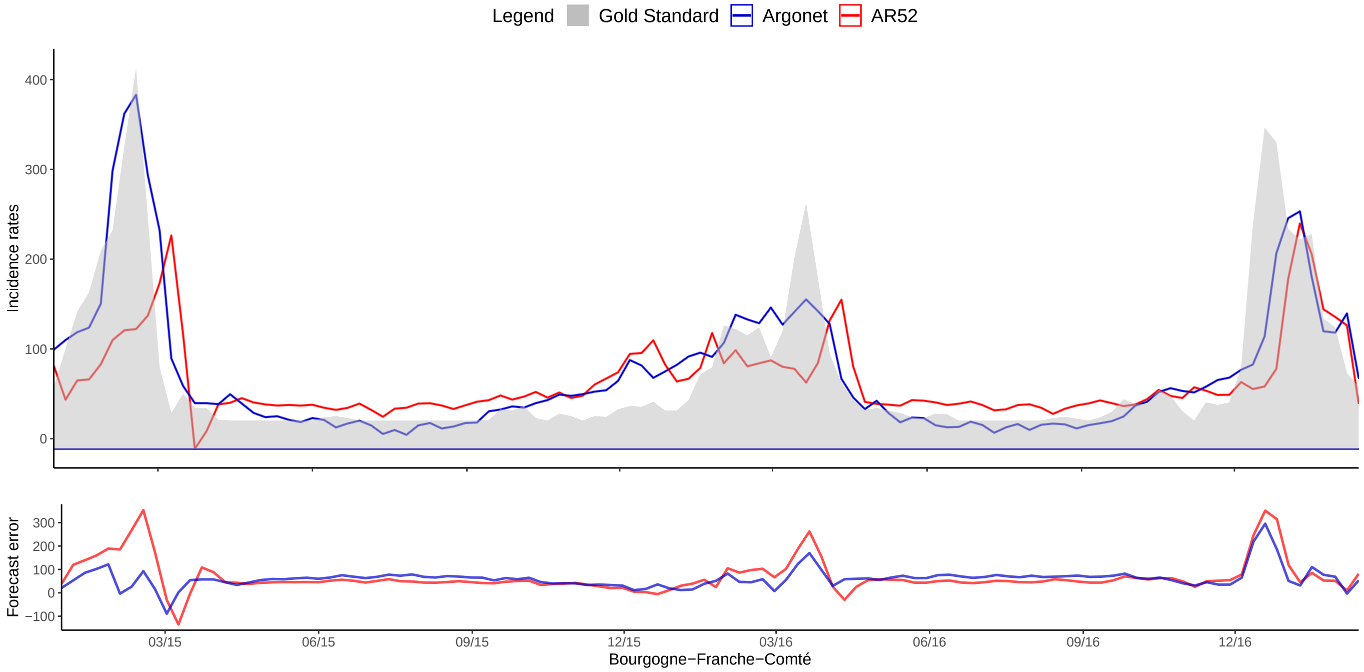

Bourgogne Two-week estimate

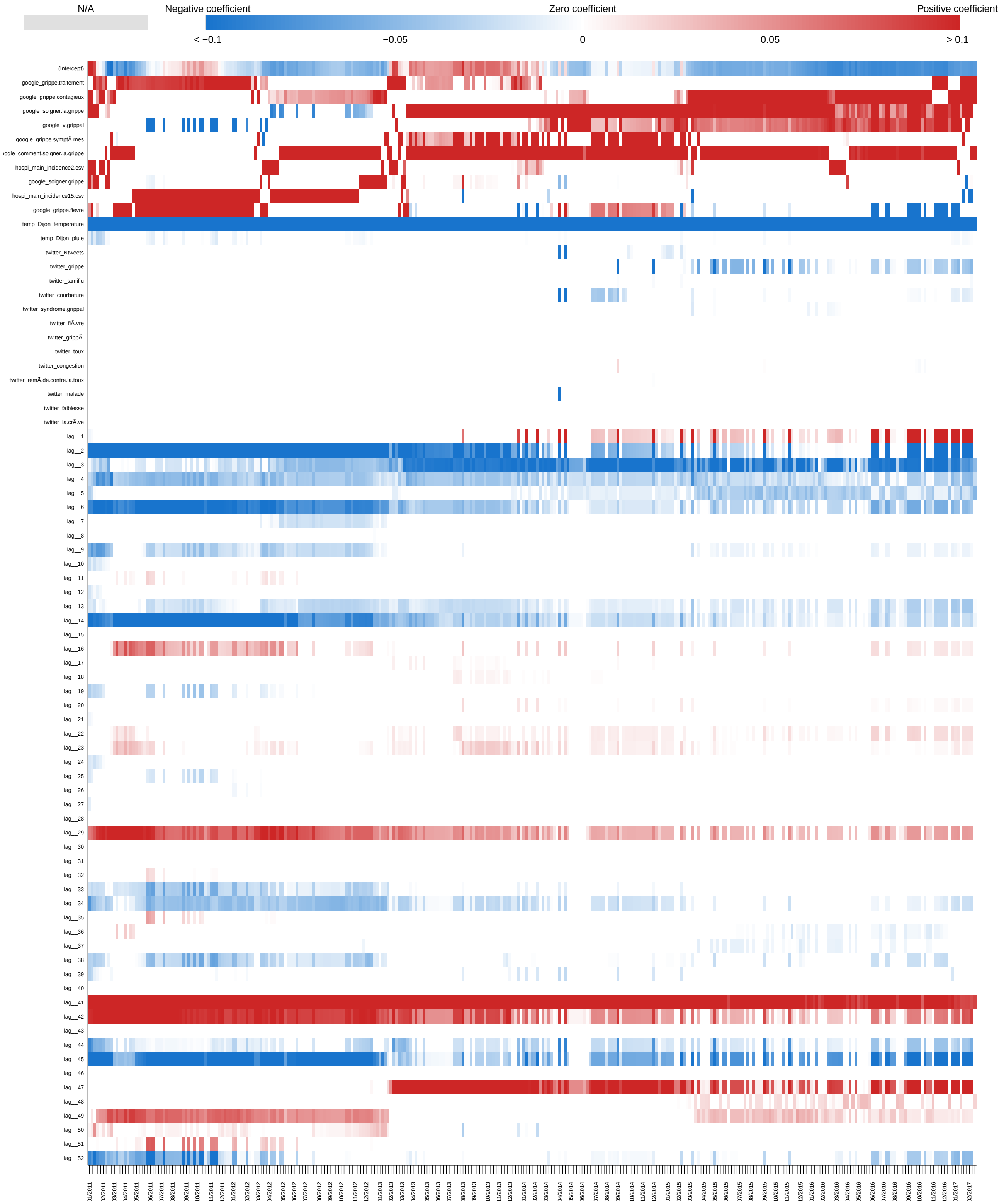

Coefficients Bourgogne Franche Comté Two-week estimate

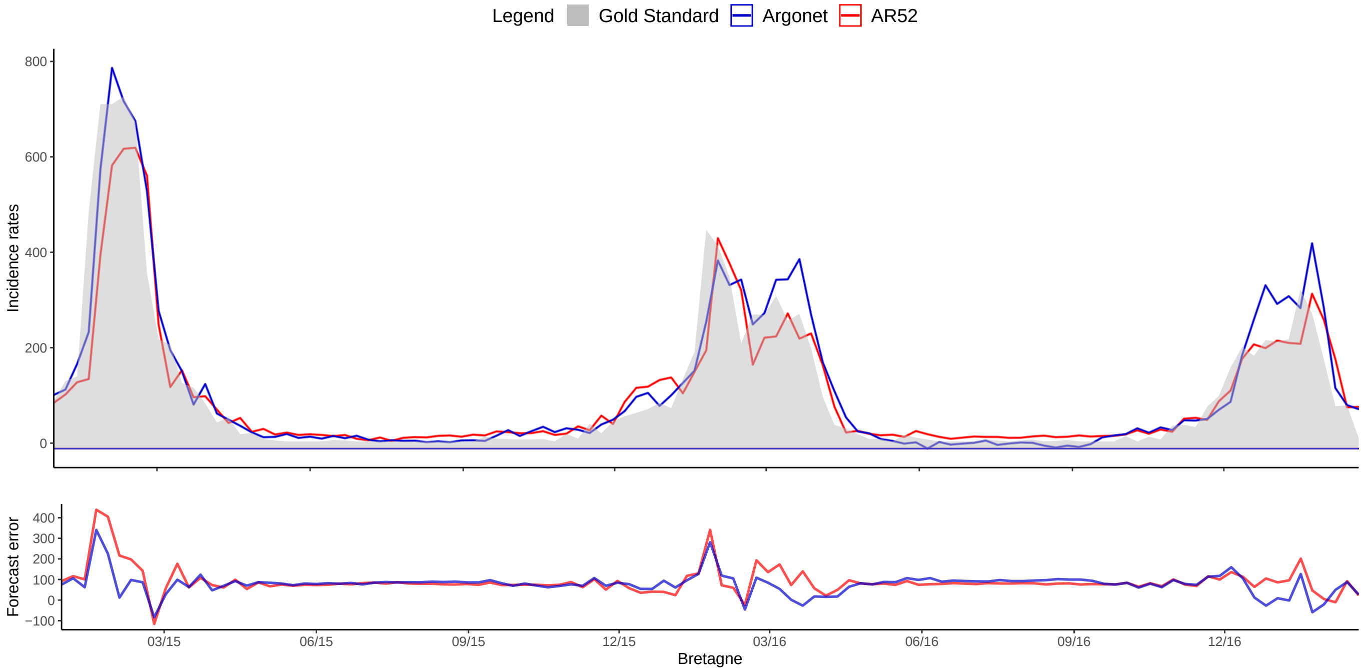

Bretagne Real-time estimate

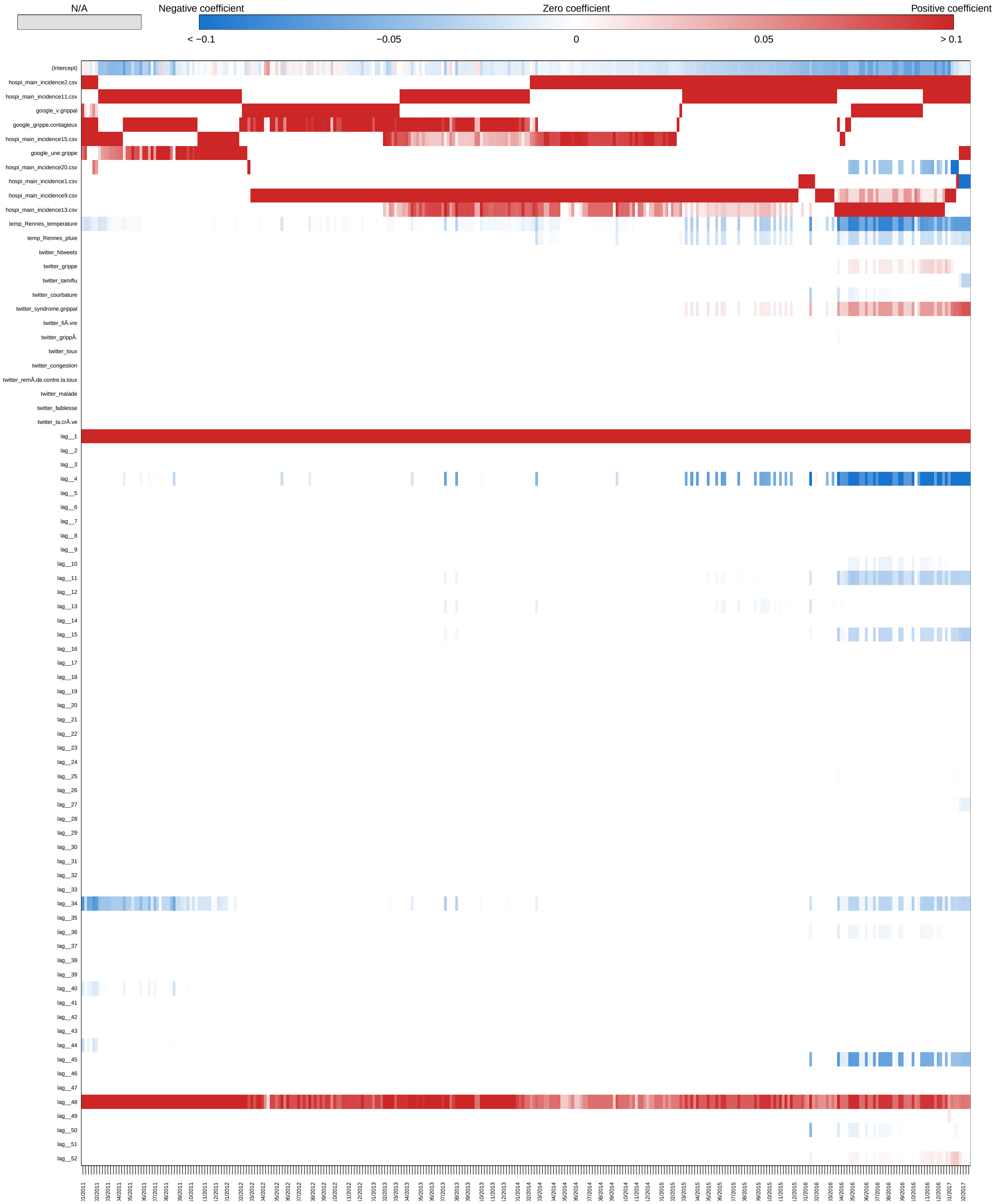

Coefficients Bretagne Real-time estimate

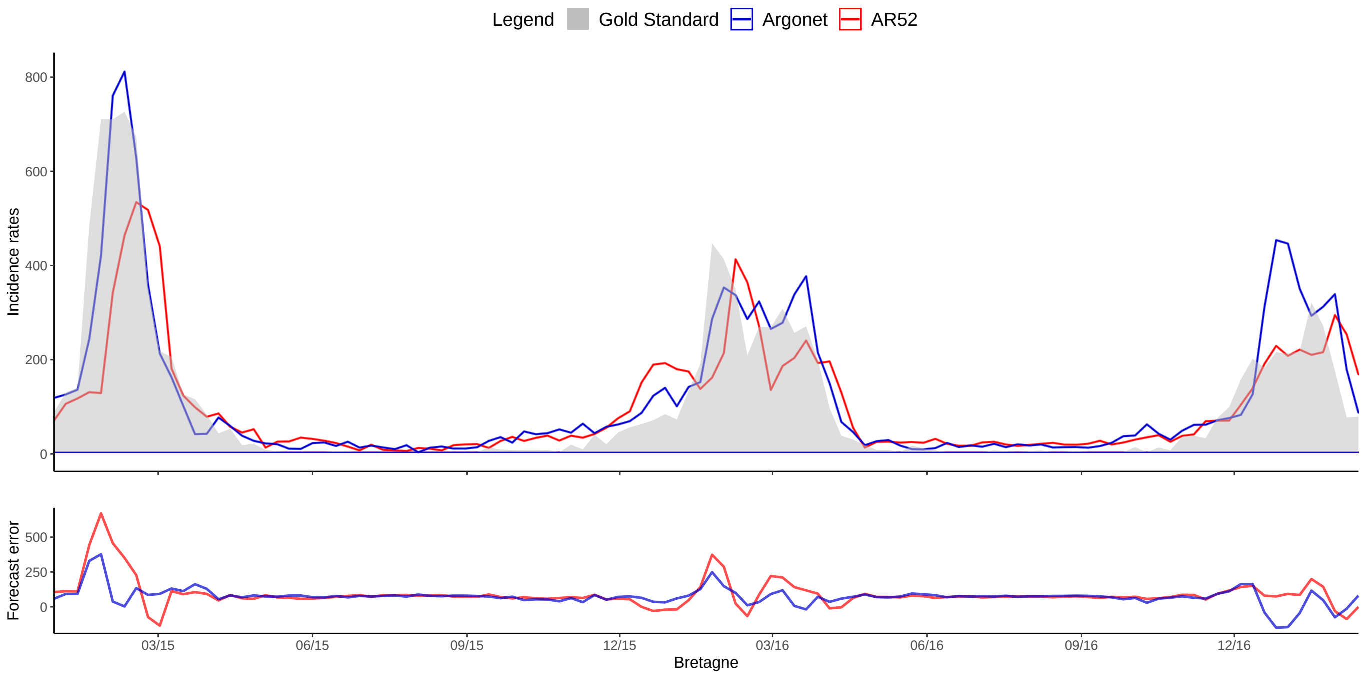

Bretagne One-week estimate

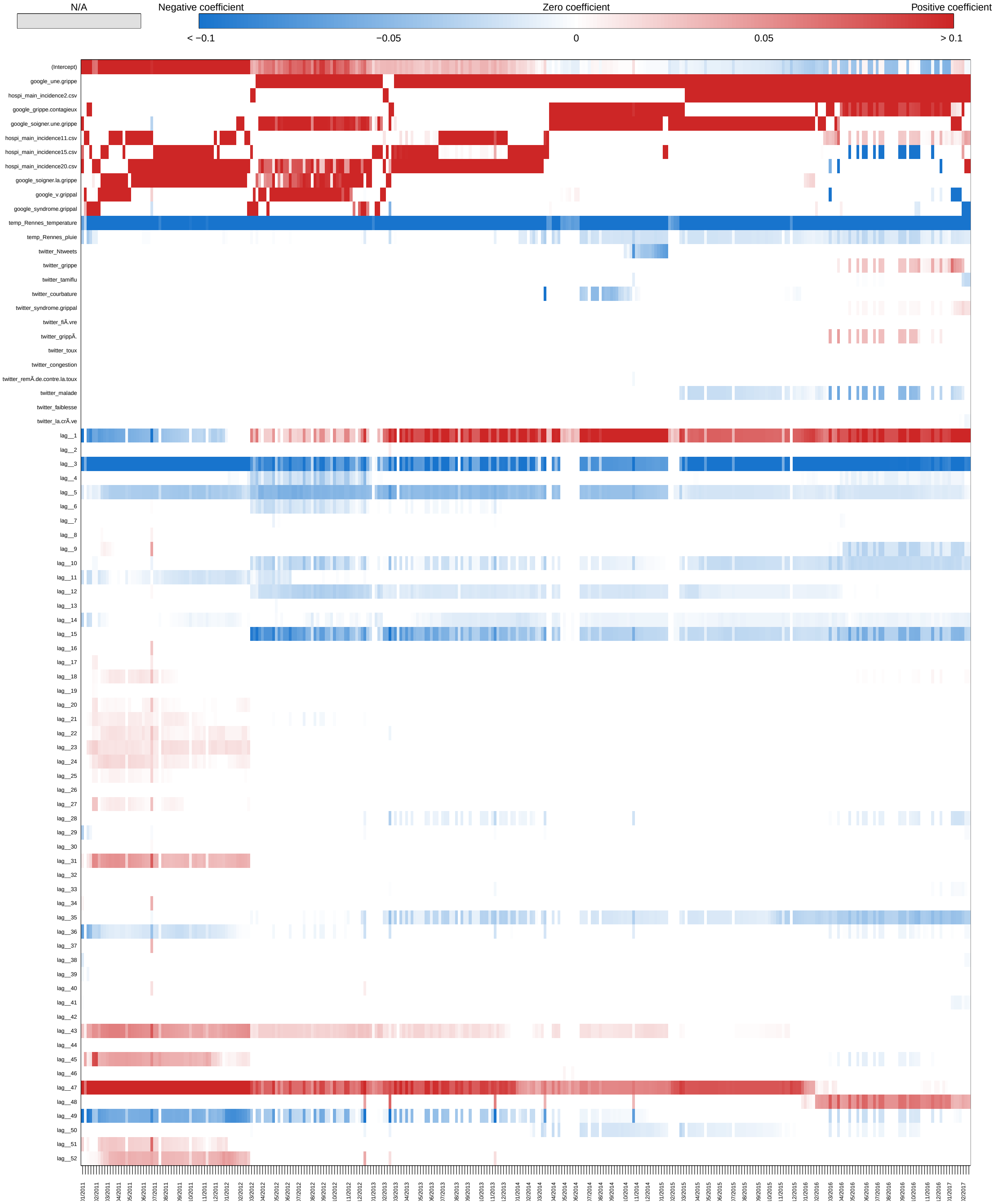

Coefficients Bretagne One-week estimate

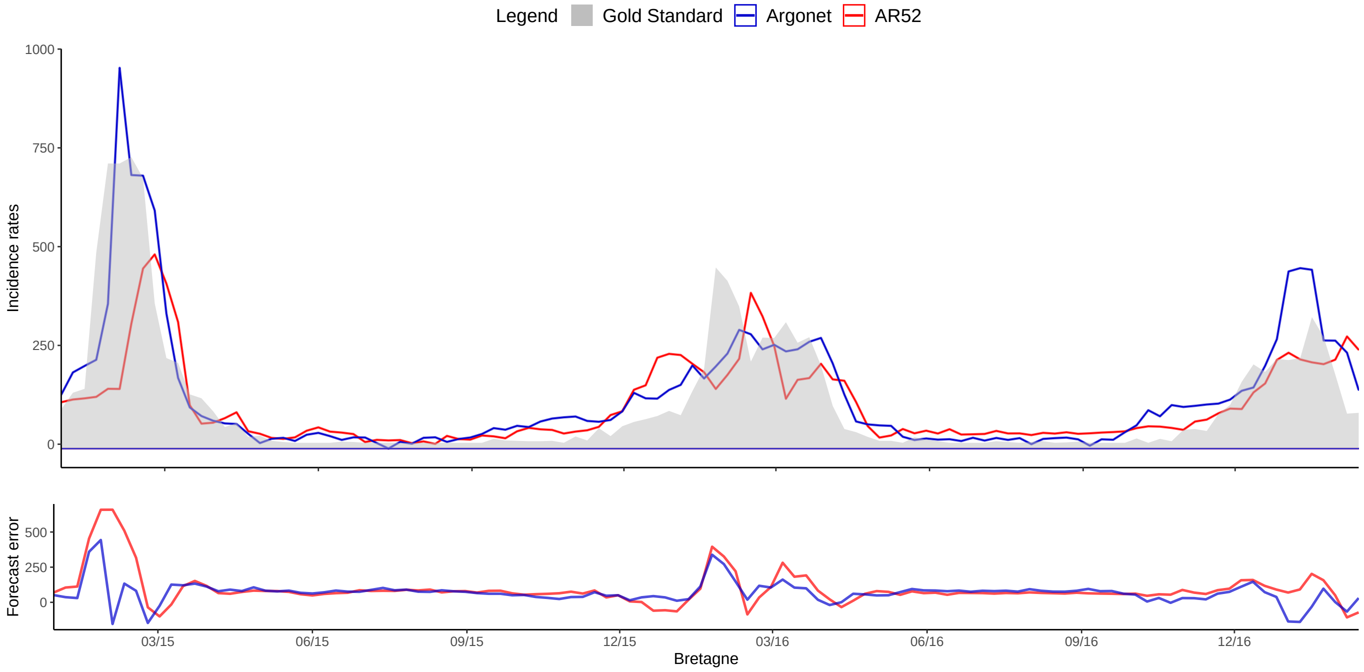

Bretagne Two-week estimate

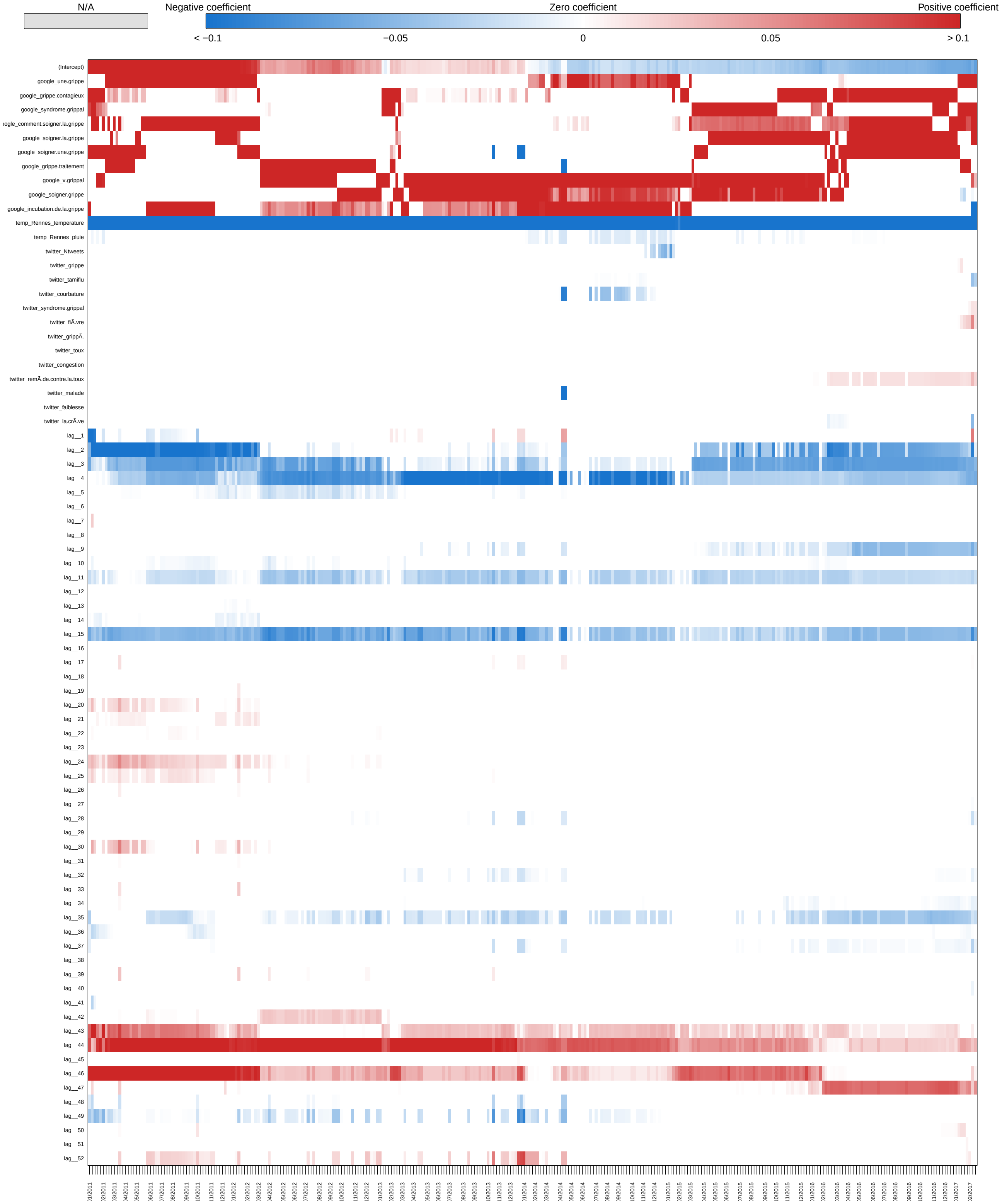

Coefficients Bretagne Two-week estimate

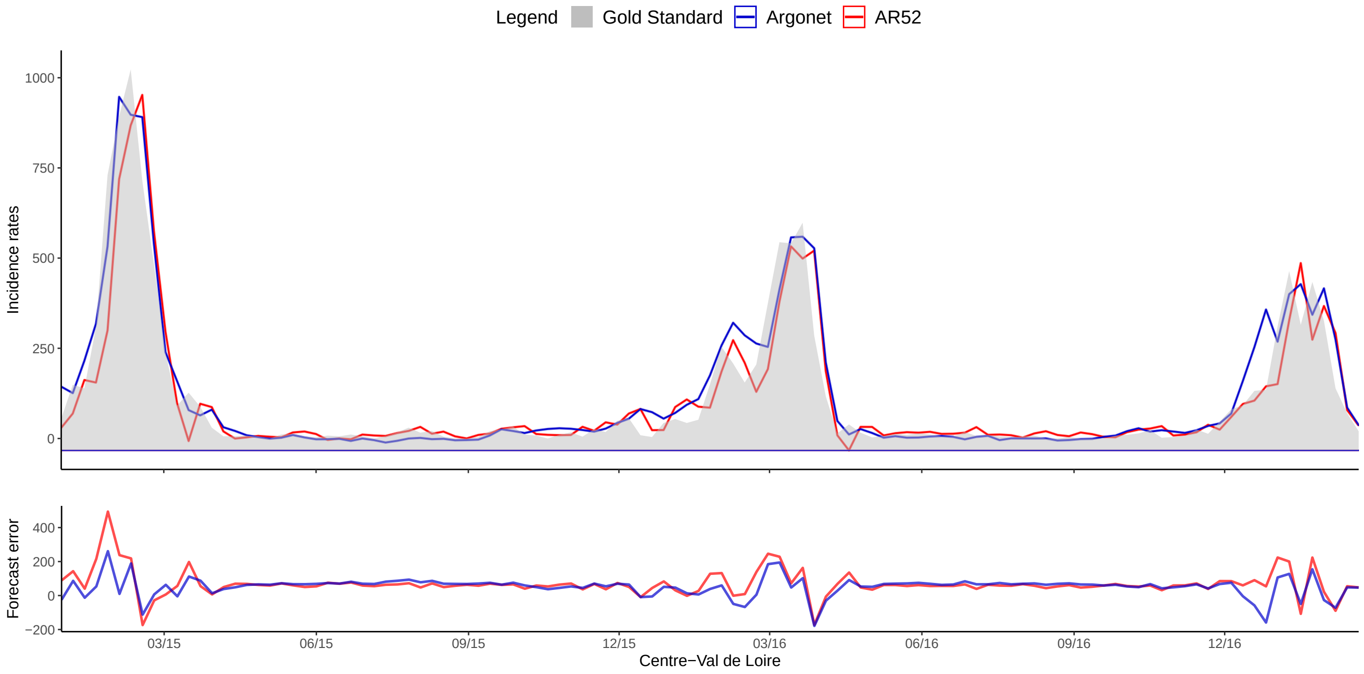

Centre Val-de-Loire Real-time estimate

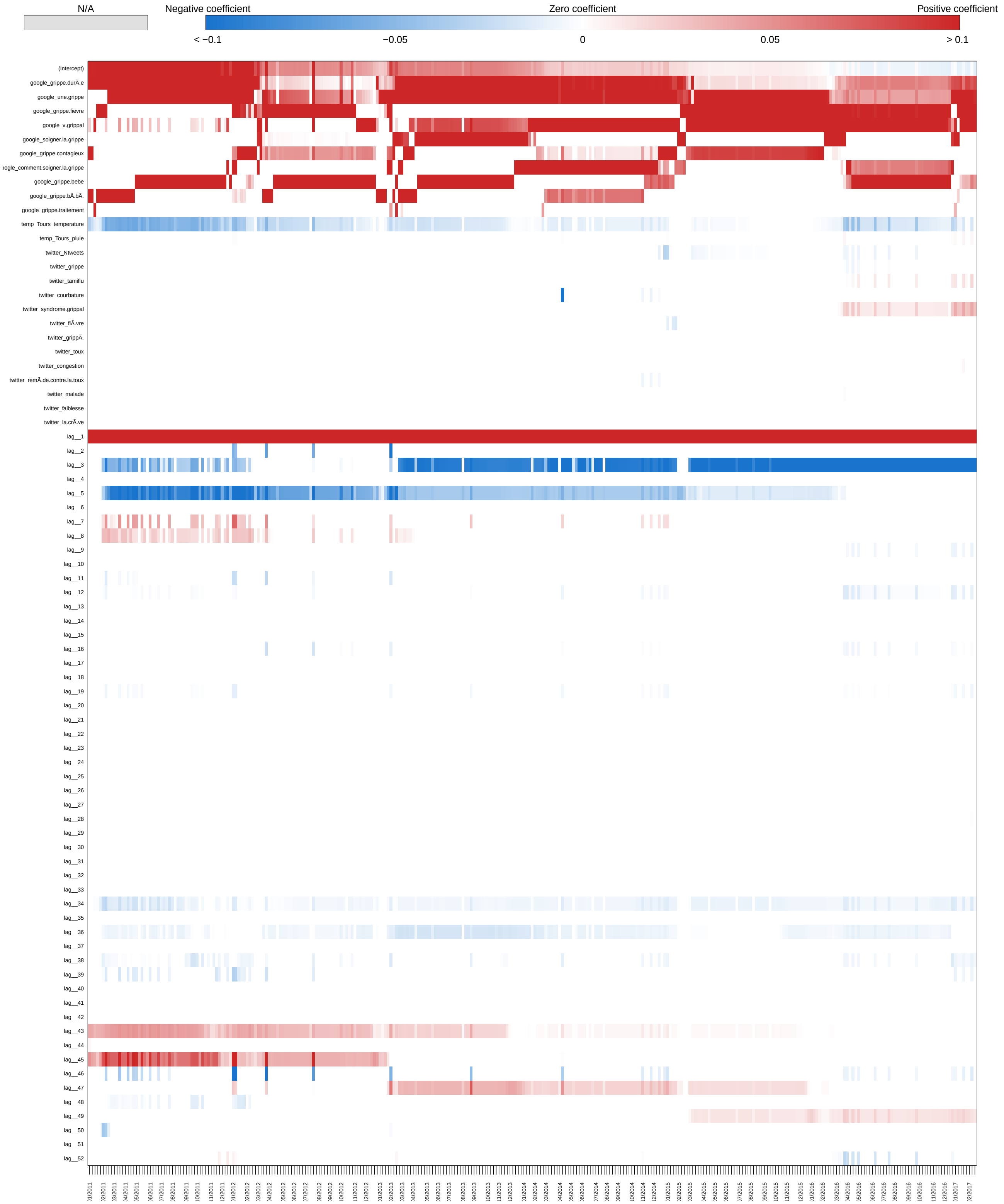

Coefficients Centre Val-de-Loire Real-time estimate

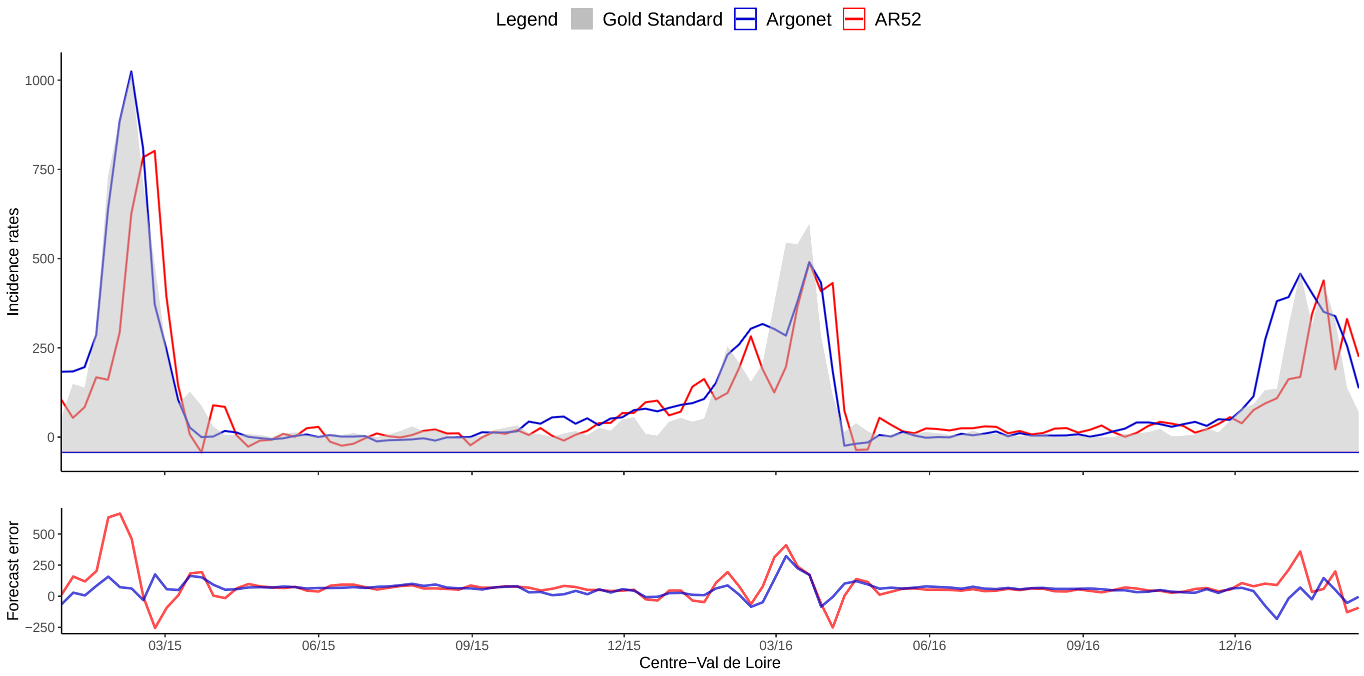

Centre Val-de-Loire One-week estimate

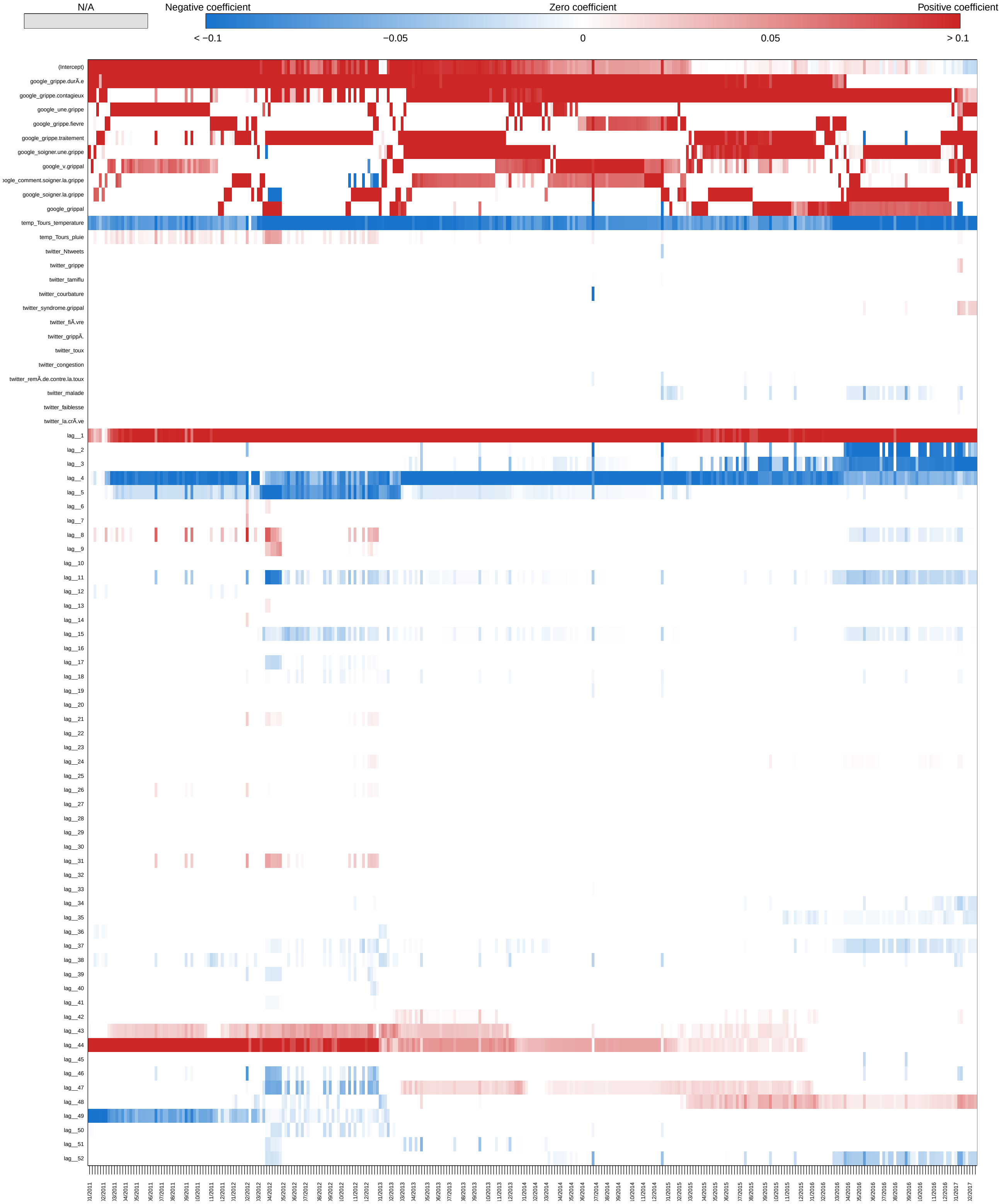

Coefficients Centre Val-de-Loire One-week estimate

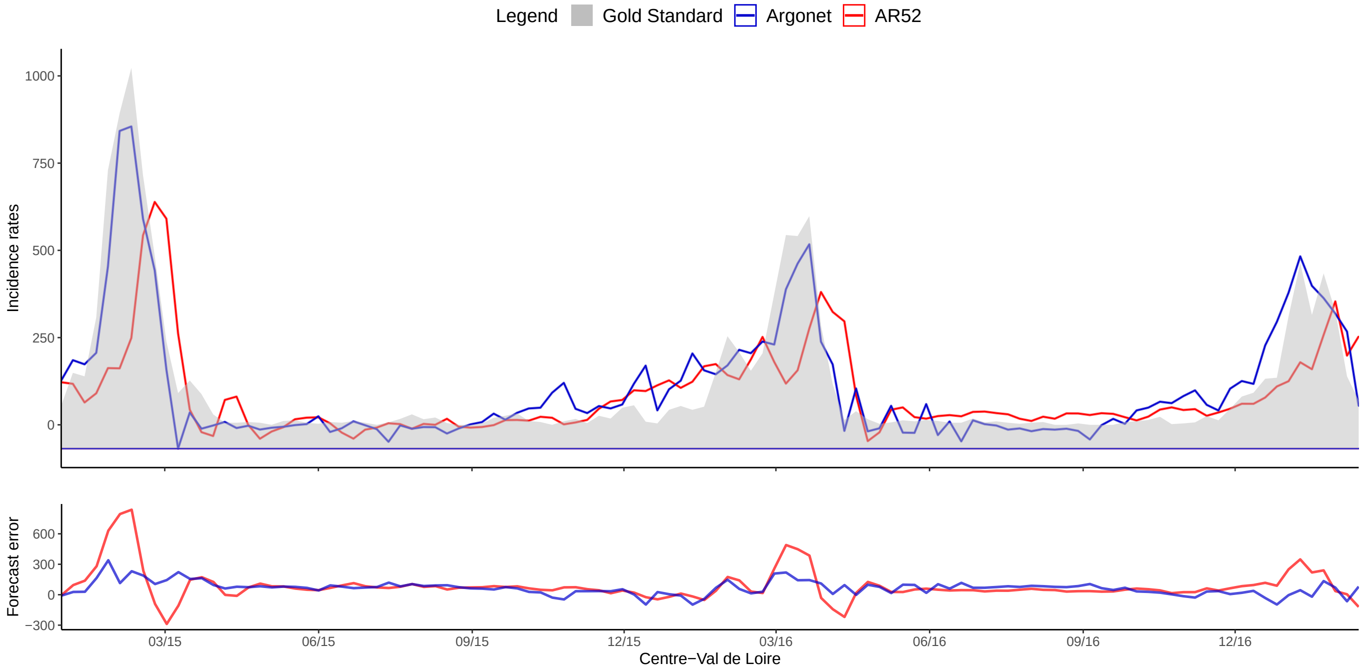

Centre Val-de-Loire Two-week estimate

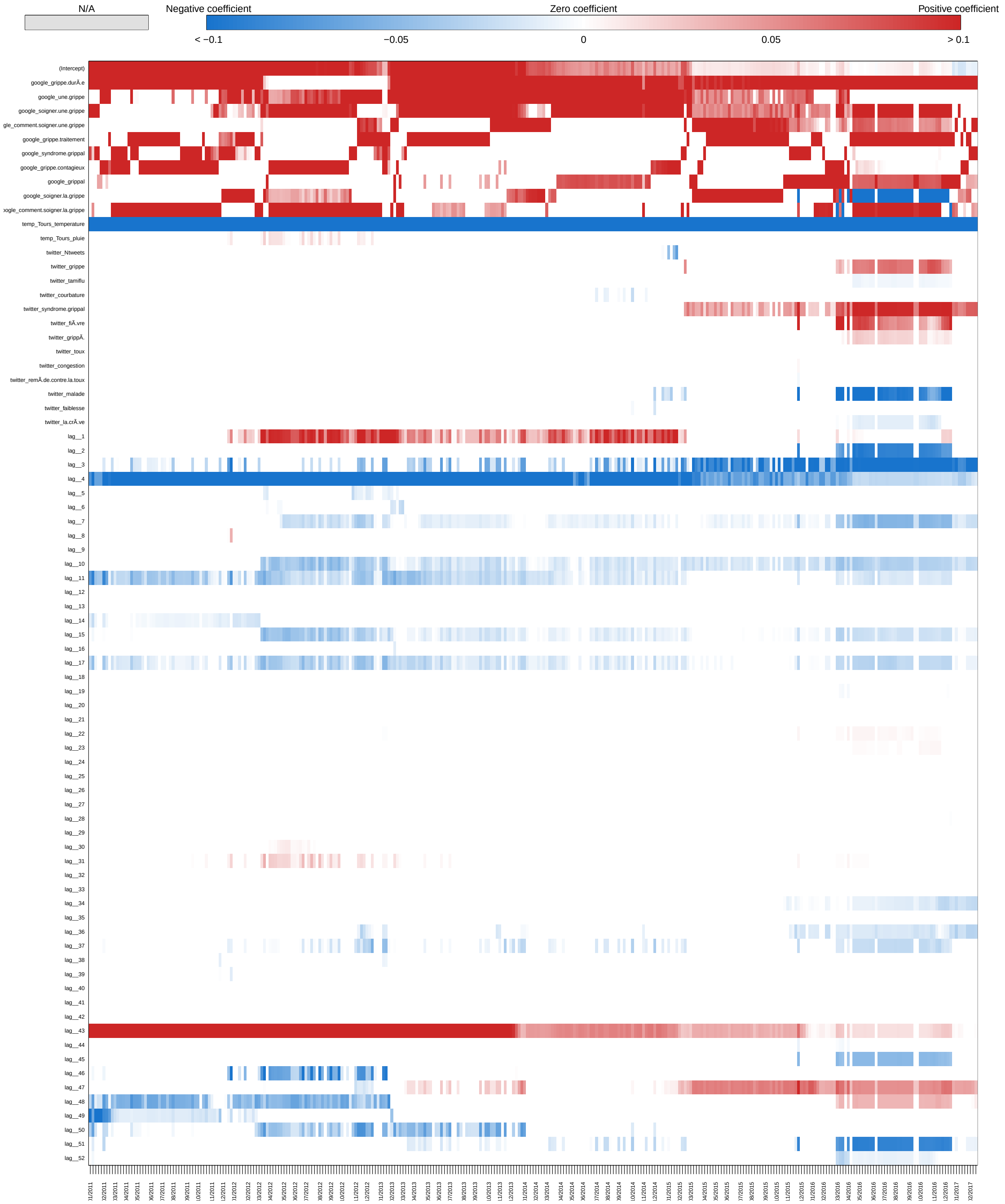

Coefficients Centre Val-de-Loire Two-week estimate

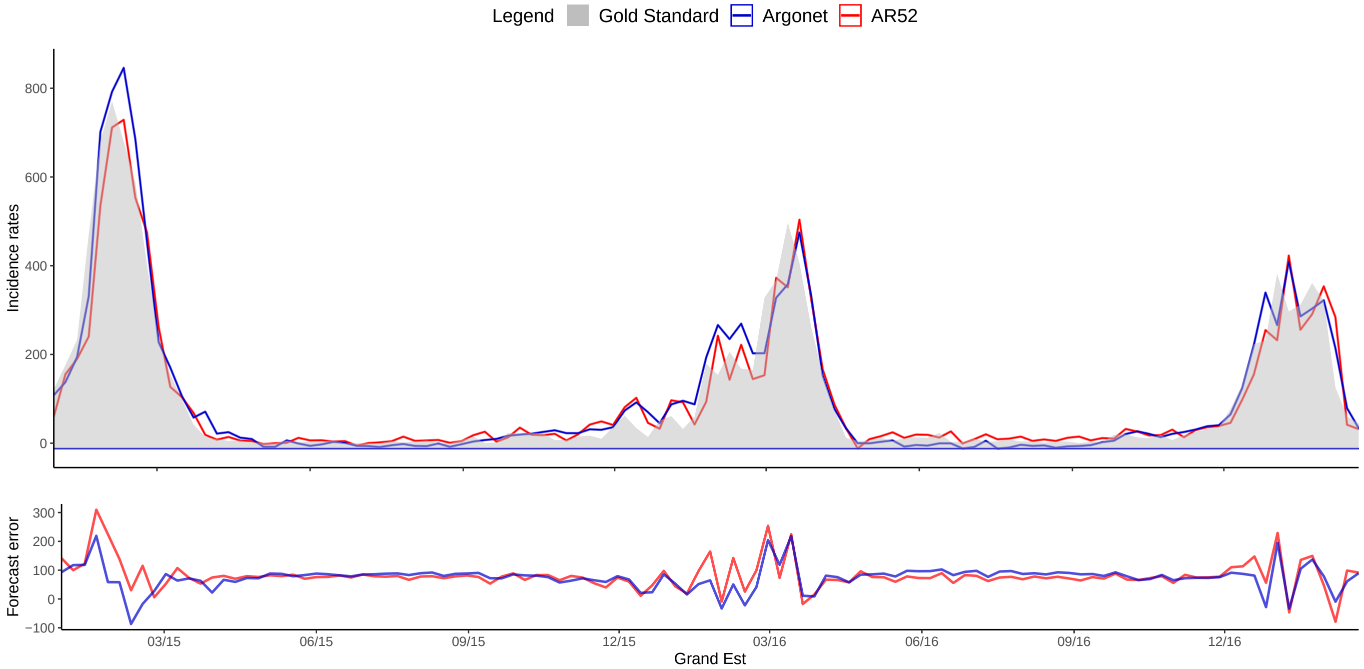

Grand Est Real-time estimate

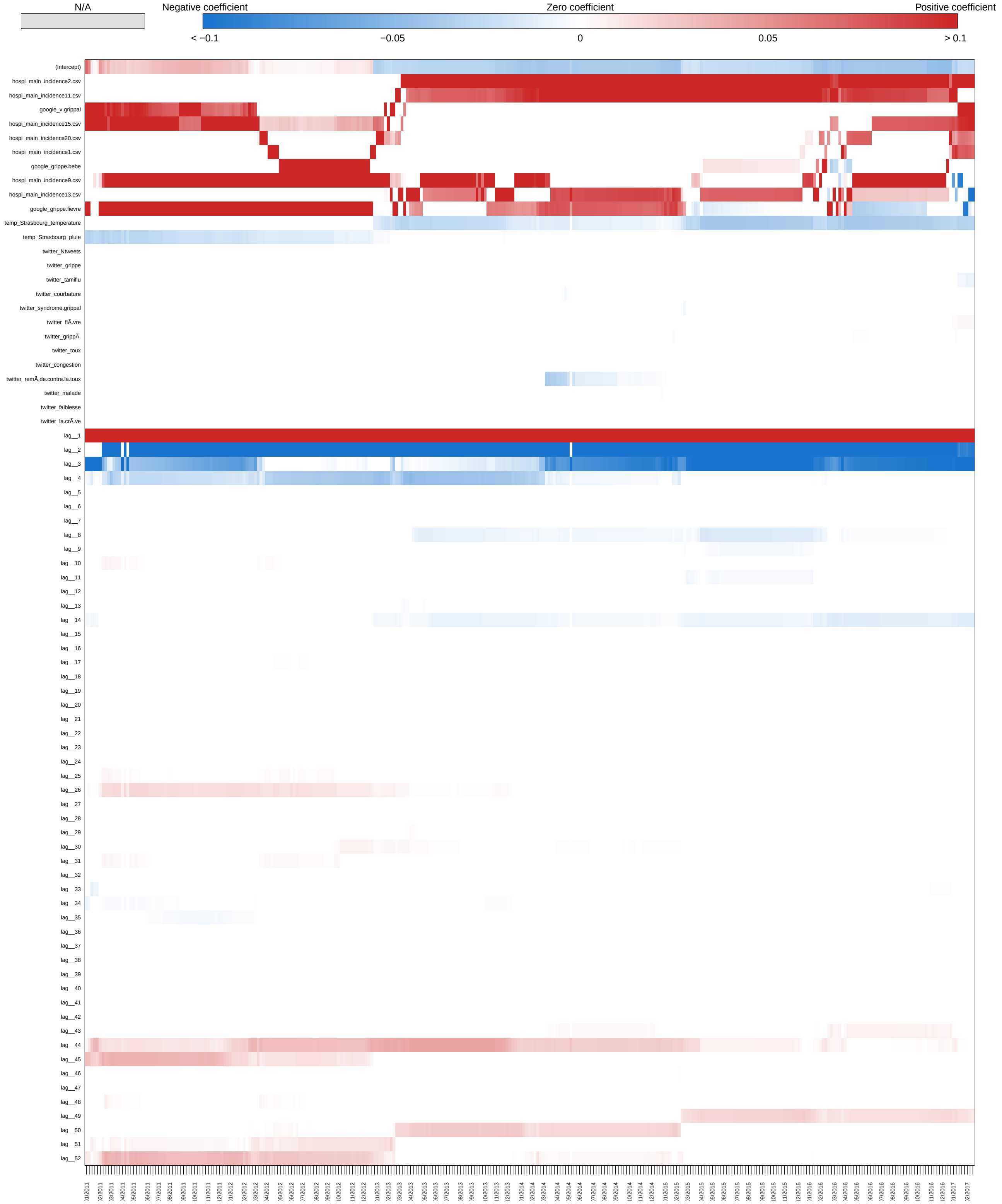

Coefficients Grand Est Real-time estimate

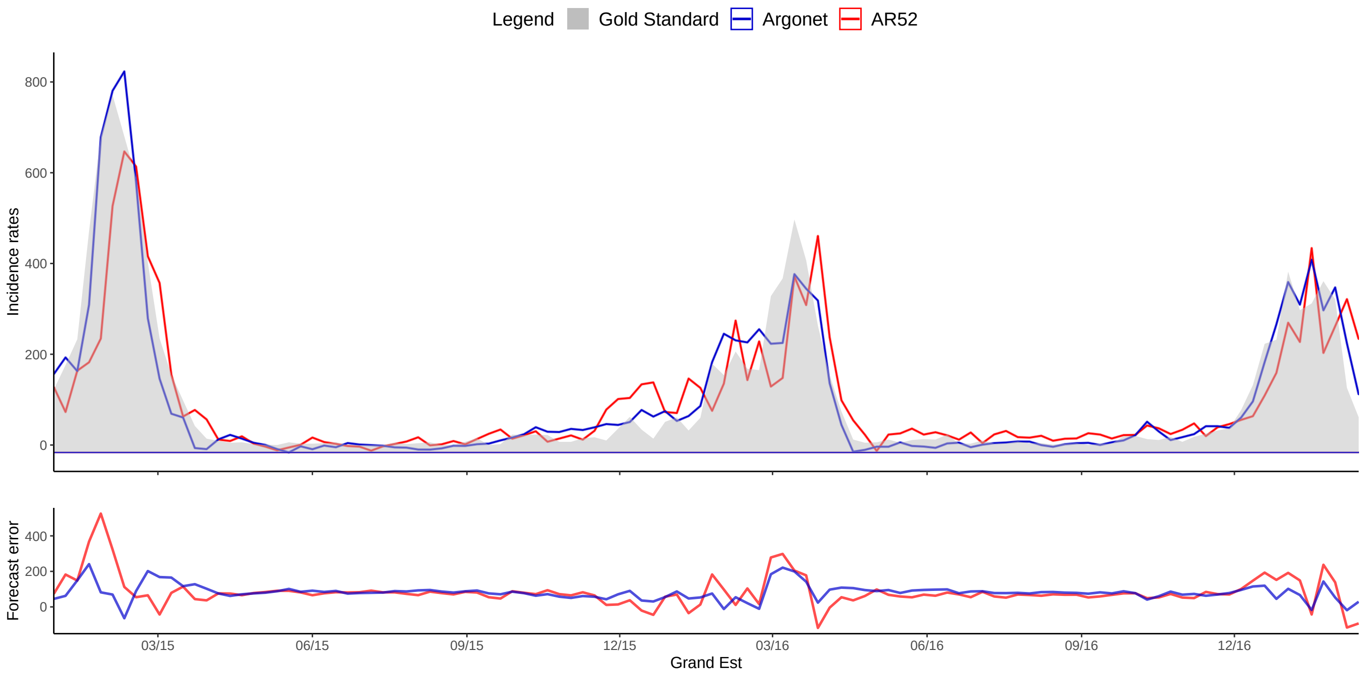

Grand Est One-week estimate

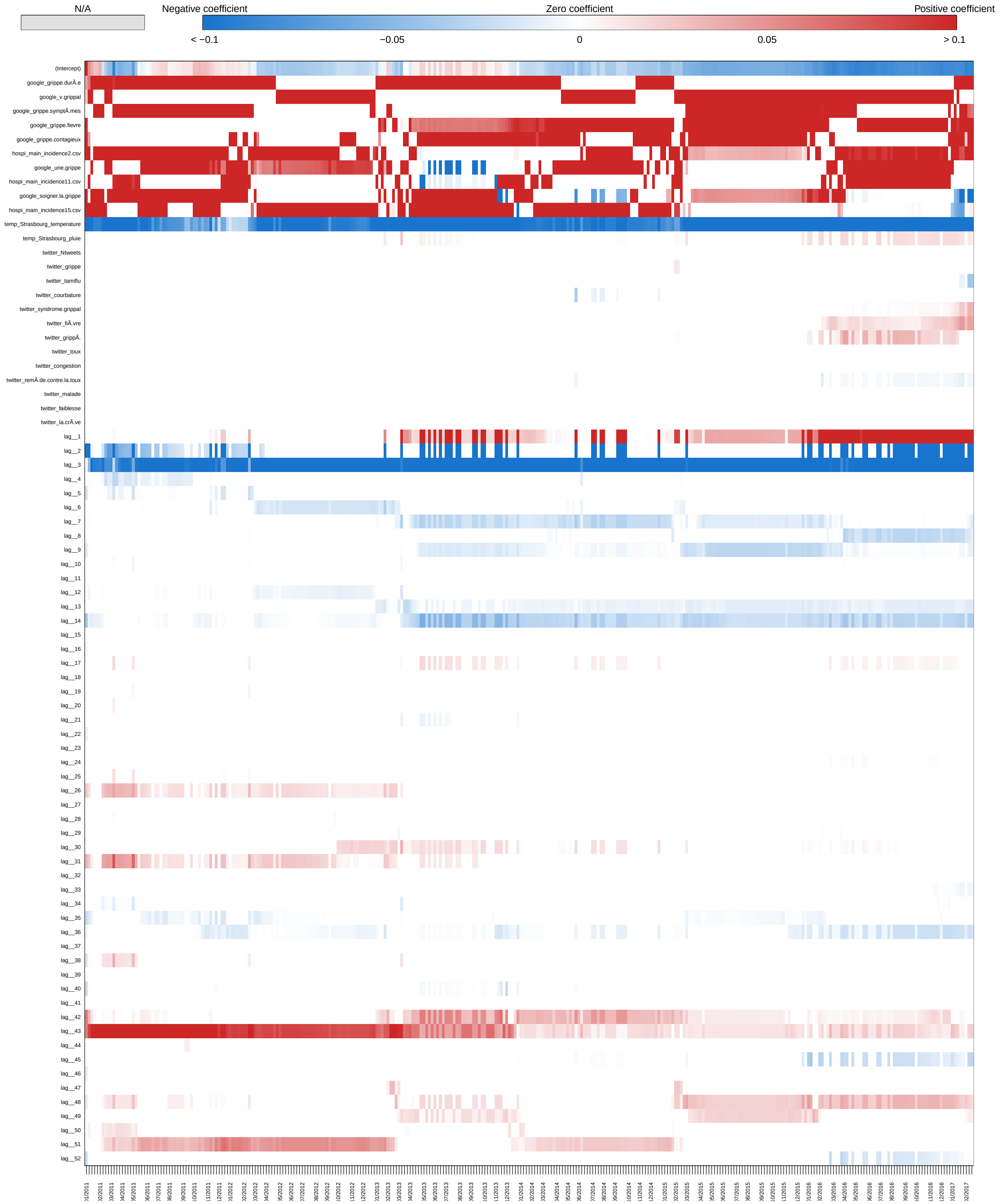

Coefficients Grand Est One-week estimate

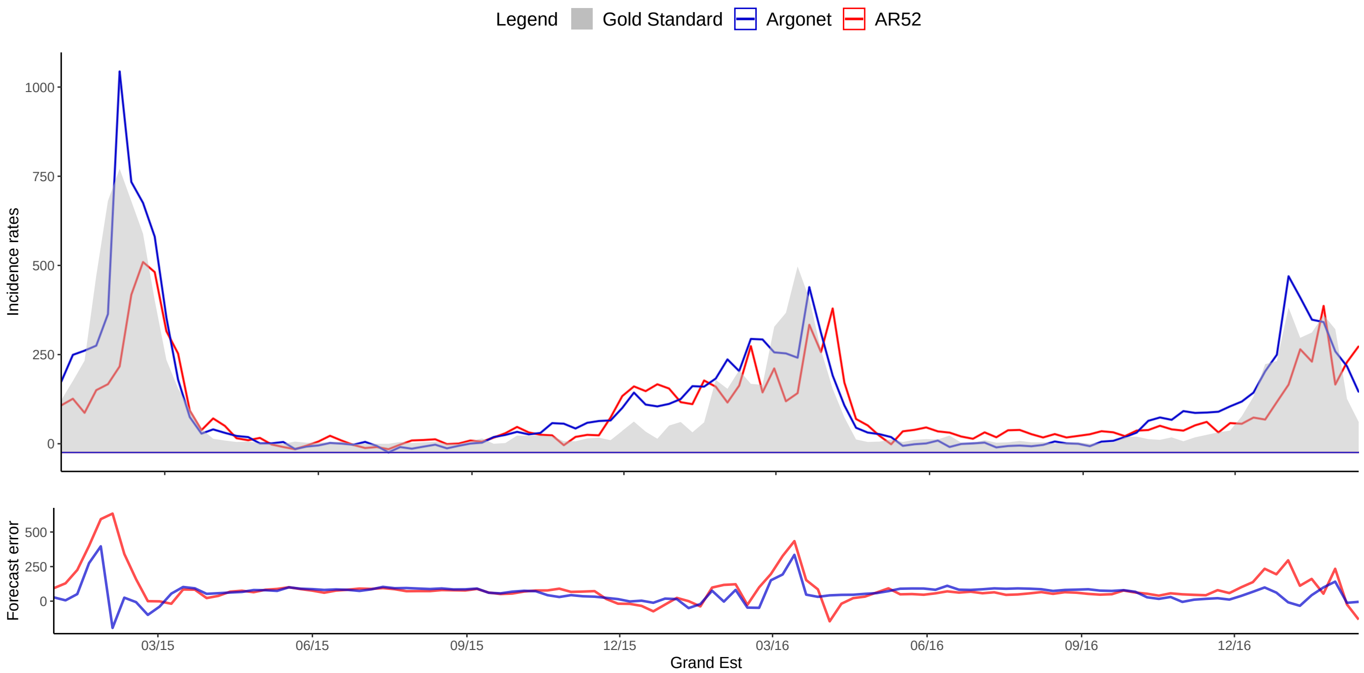

Grand Est Two-week estimate

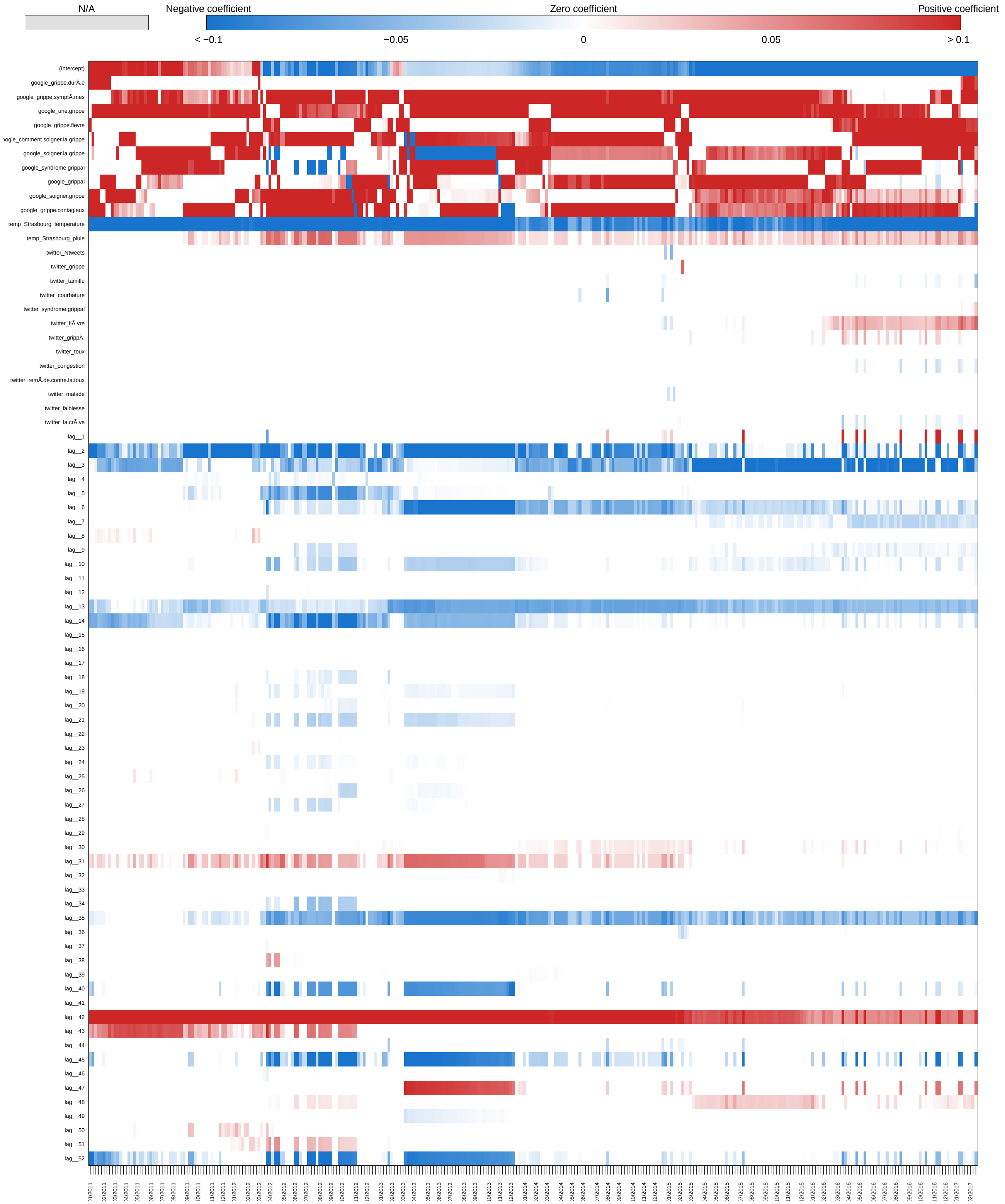

Coefficients Grand Est Two-week estimate

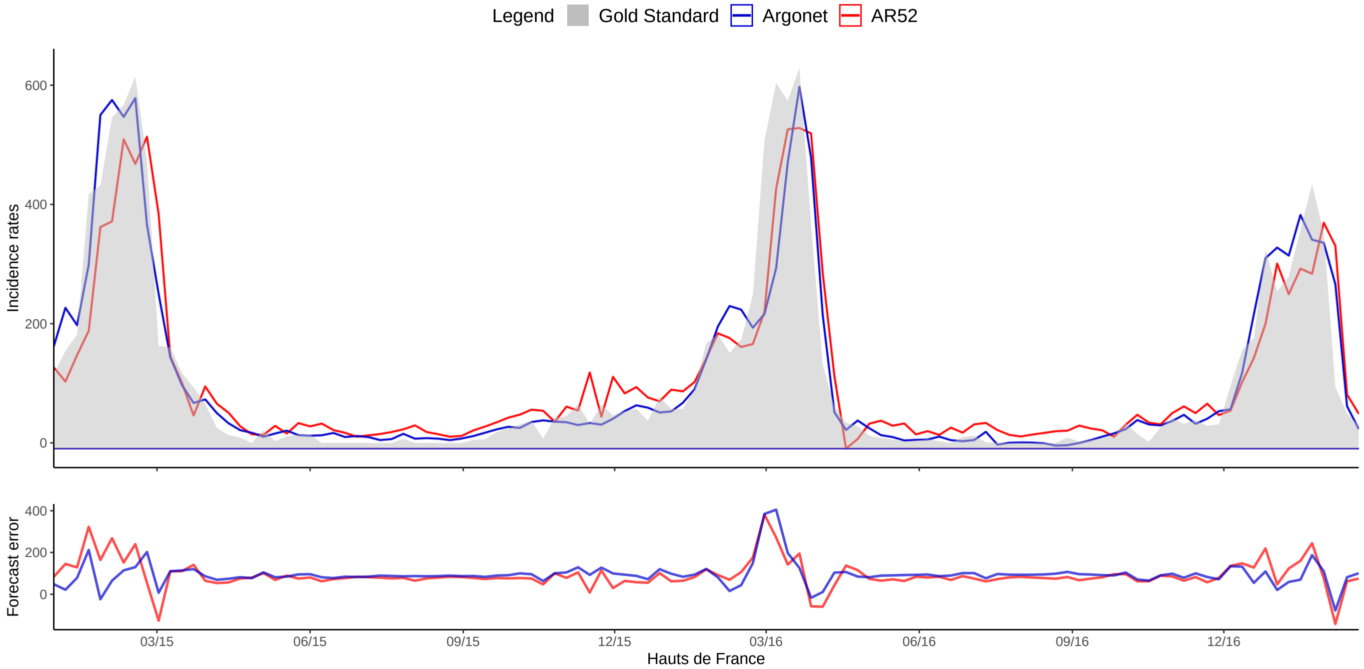

Hauts de France Real-time estimate

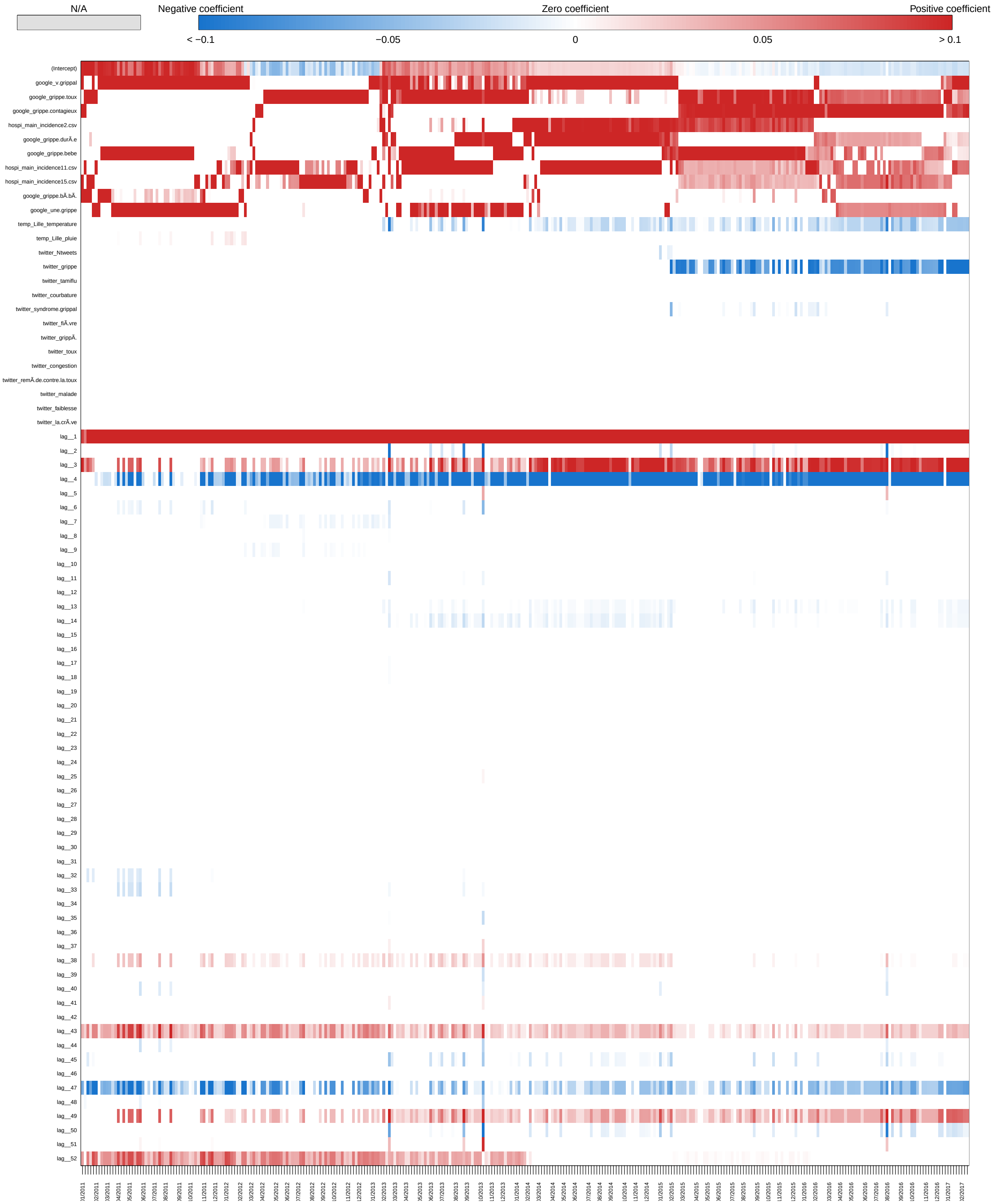

Coefficients Hauts de France Real-time estimate

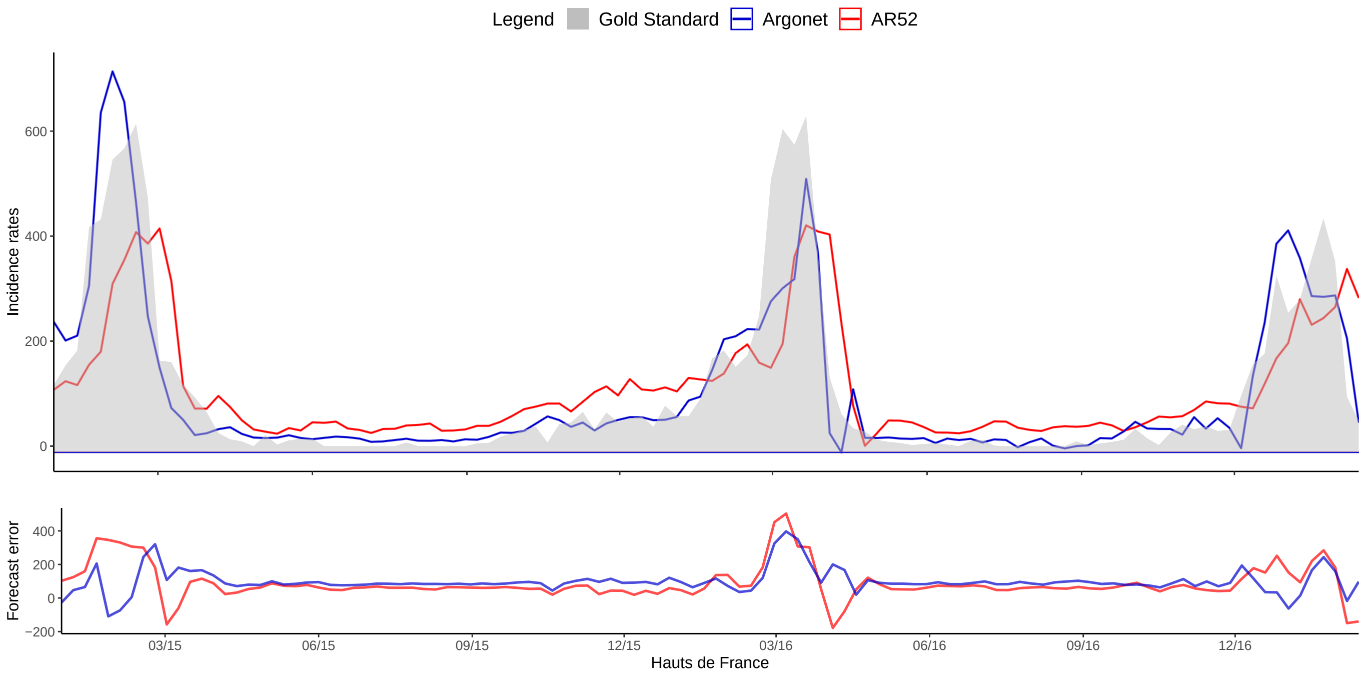

Hauts de France One-week estimate

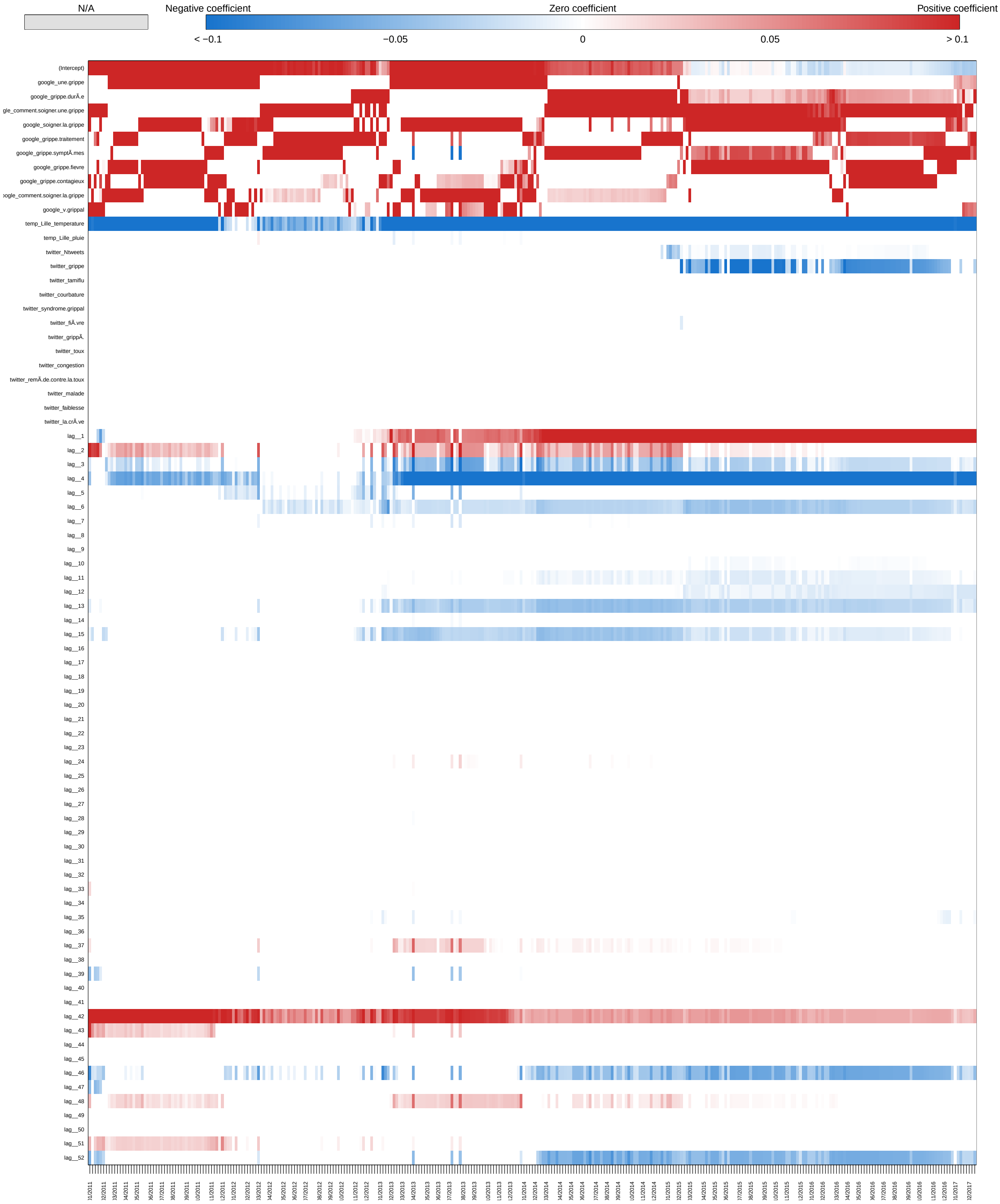

Coefficients Hauts de France One-week estimate

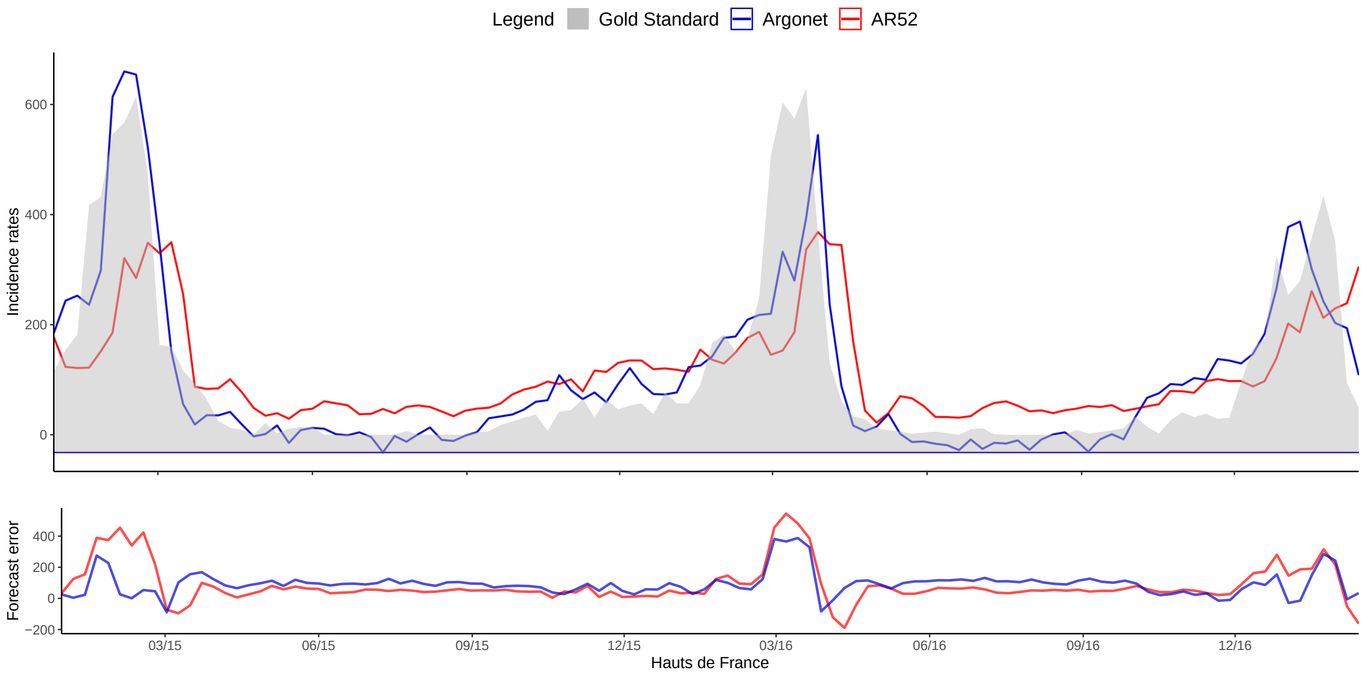

Hauts de France Two-week estimate

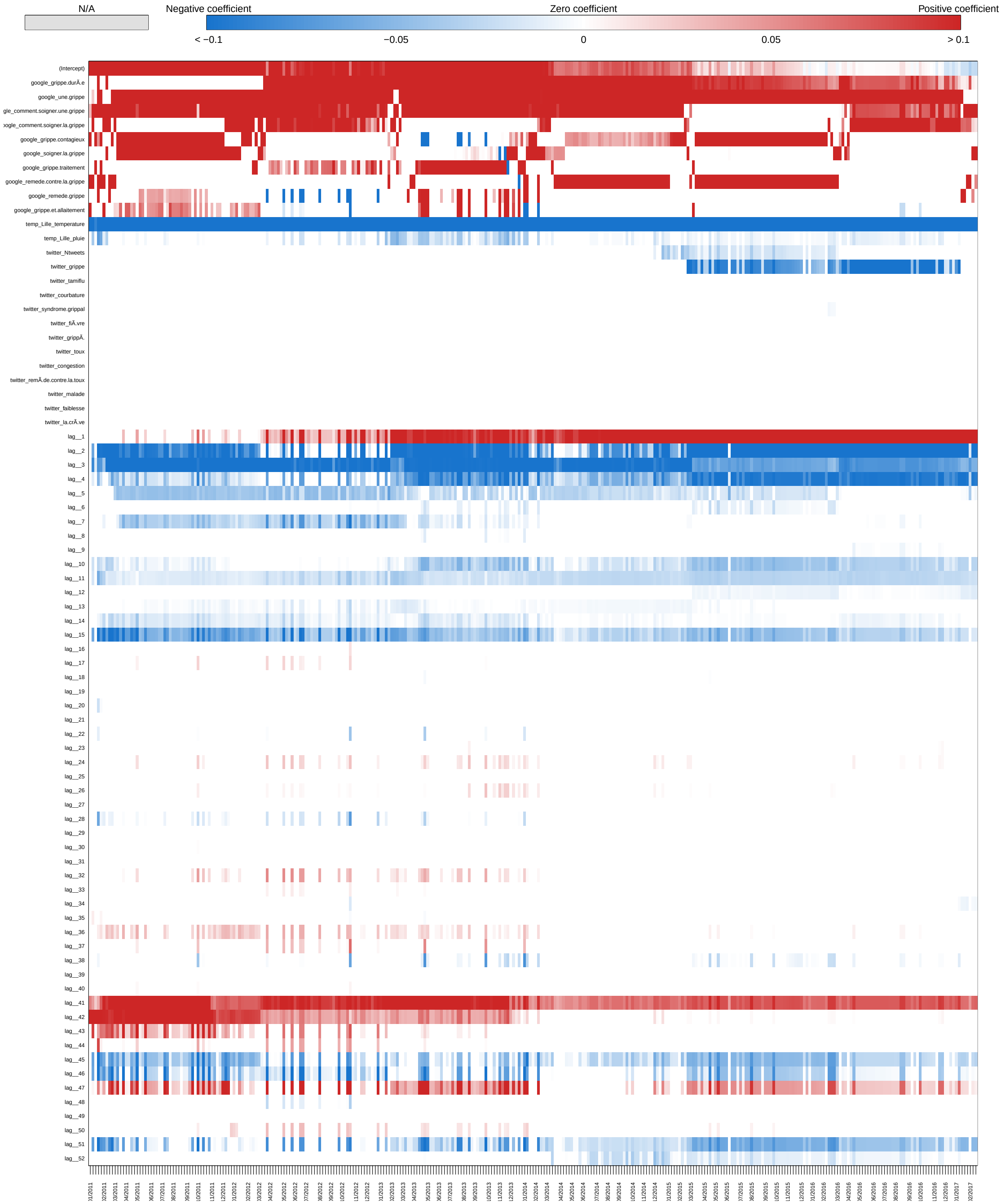

Coefficients Hauts de France Two-week estimate

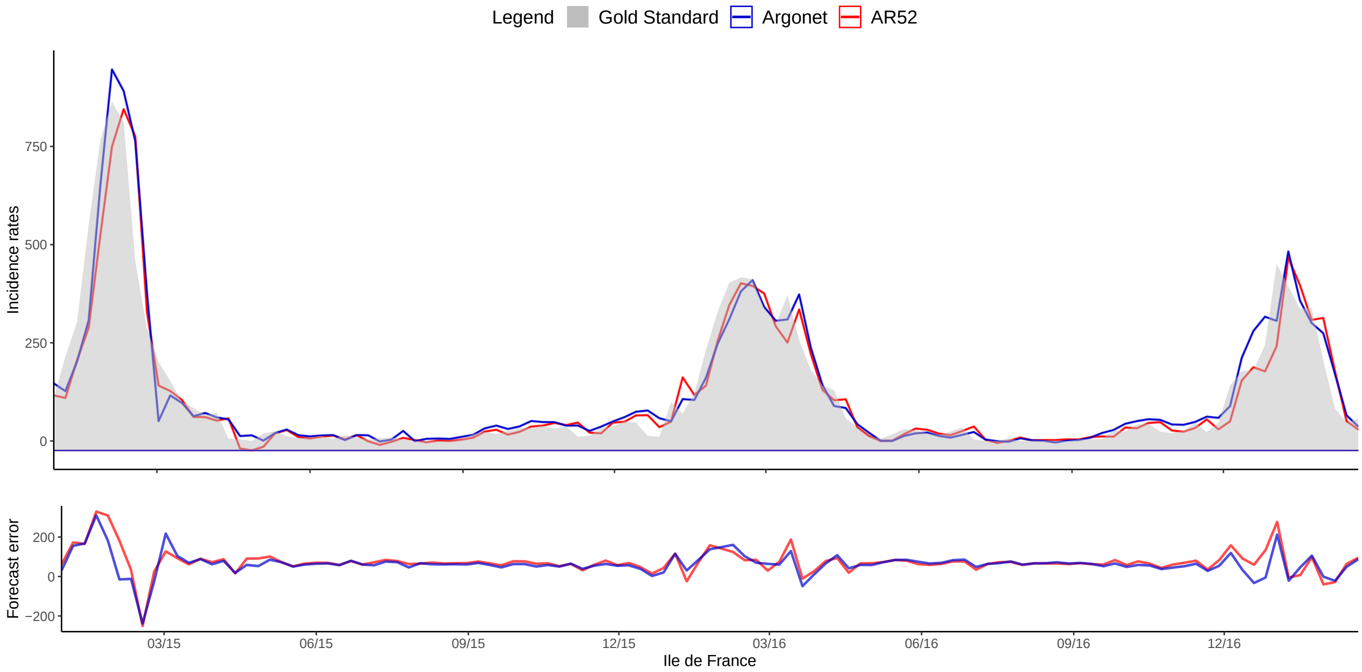

Ile de France Real-time estimate

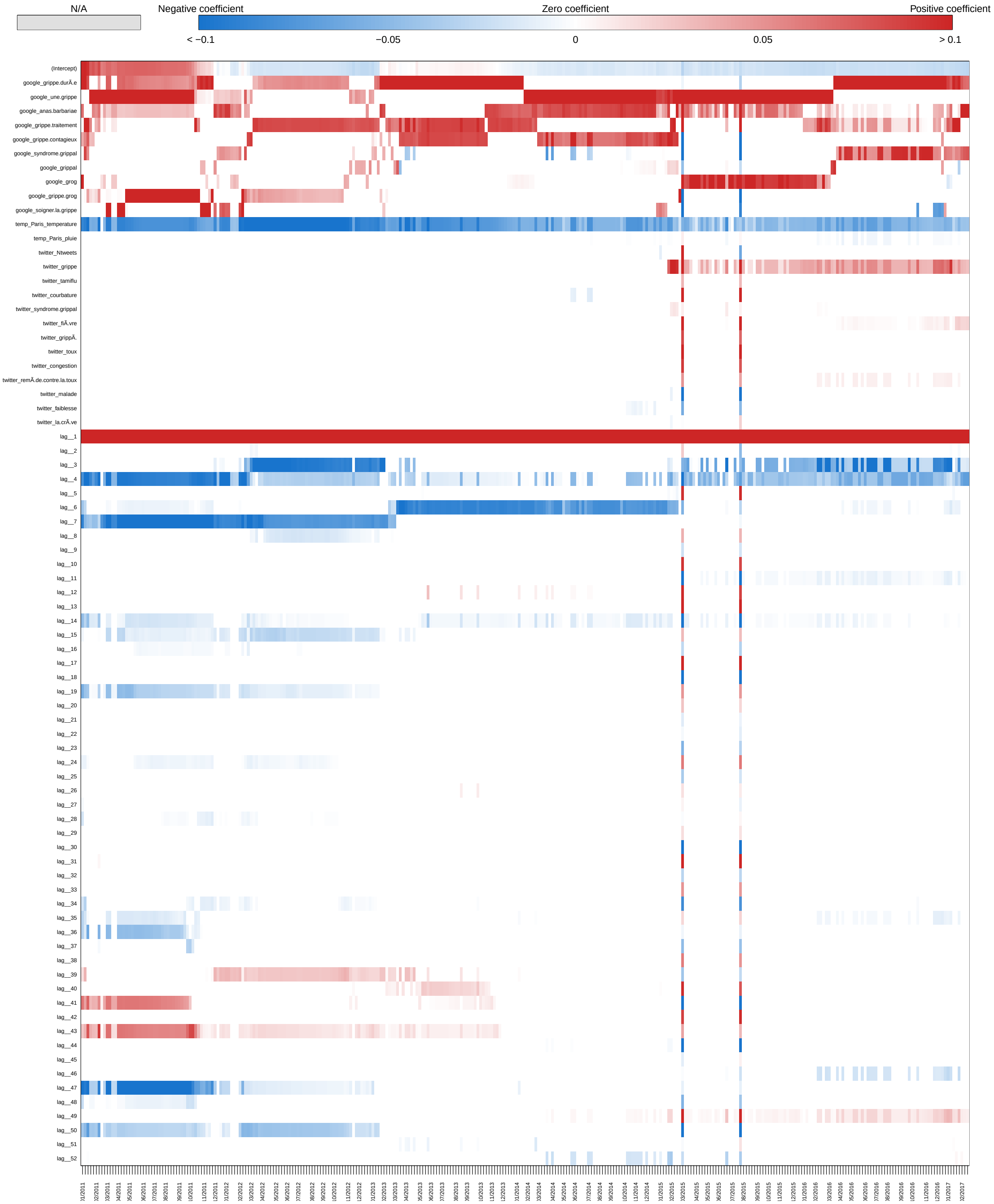

Coefficients Ile de France Real-time estimate

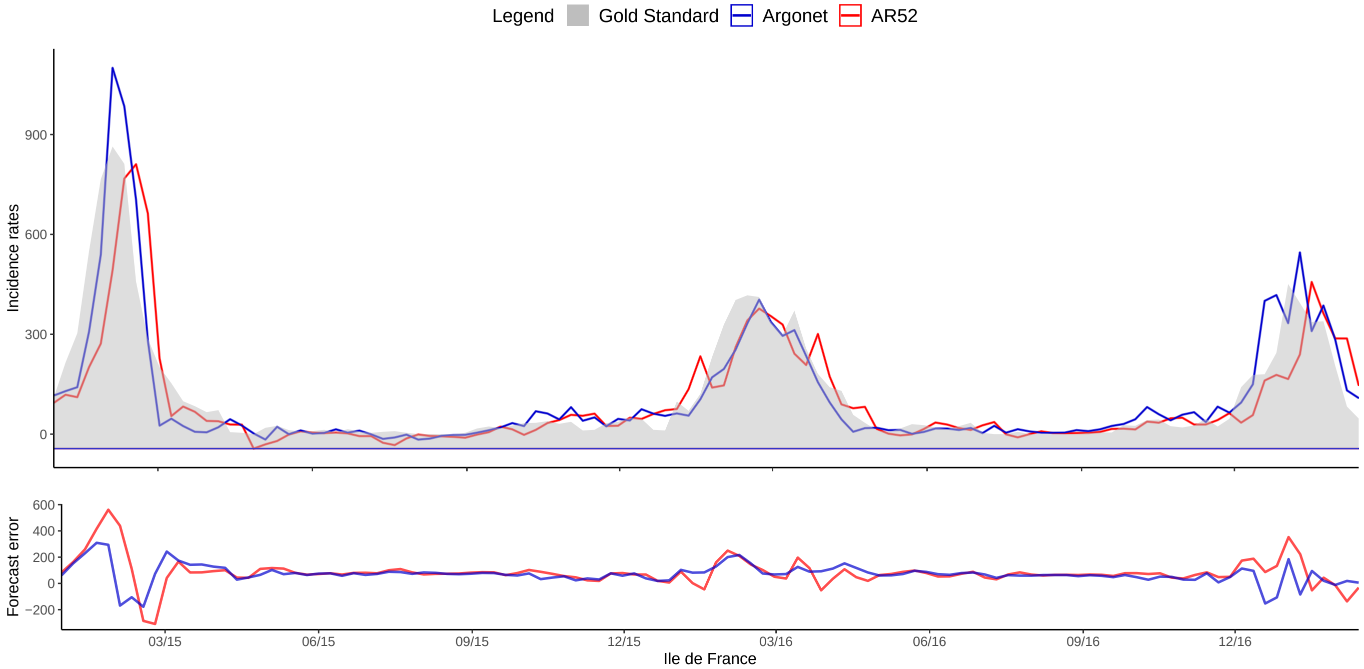

Ile de France One-week estimate

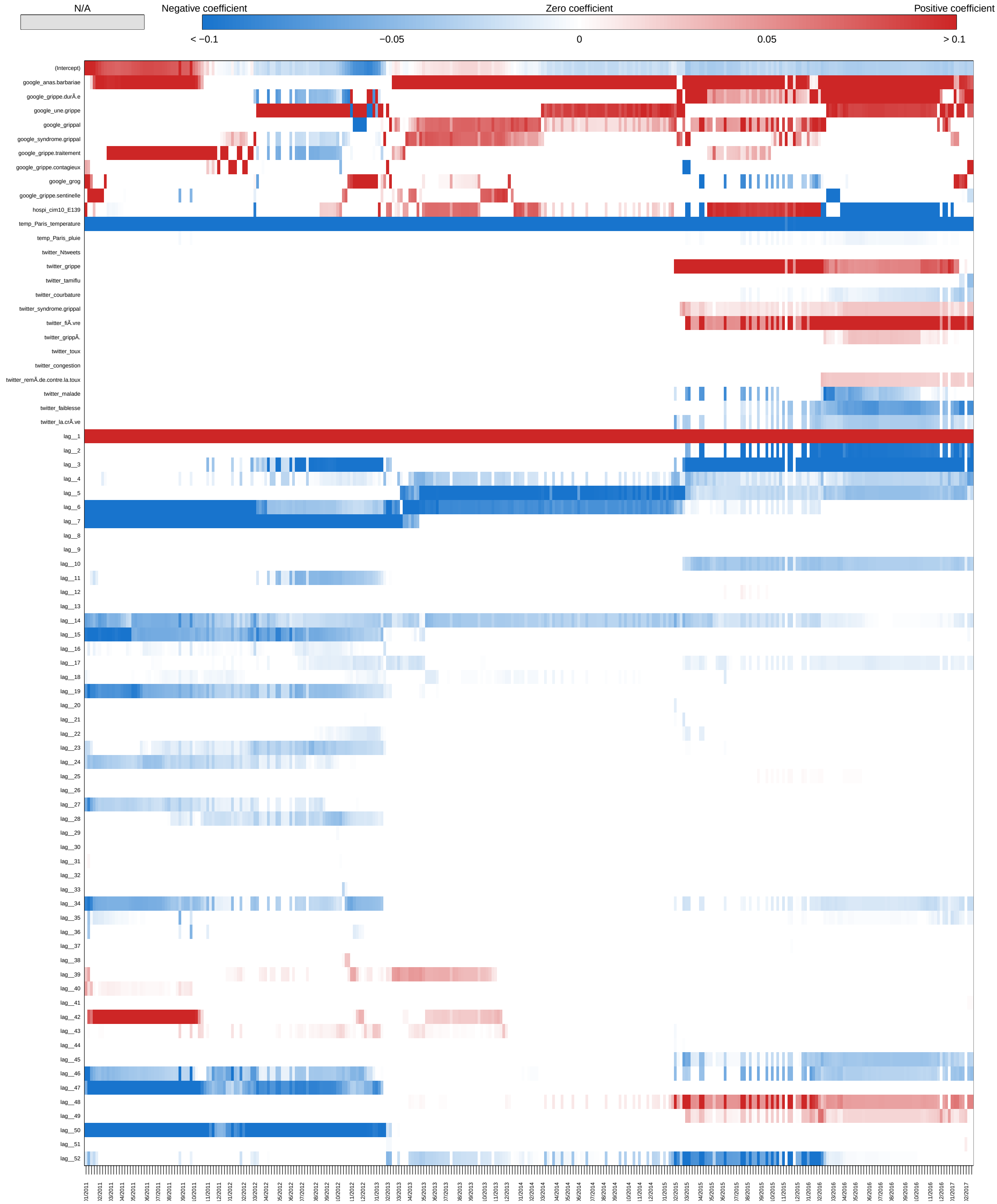

Coefficients Ile de France One-week estimate

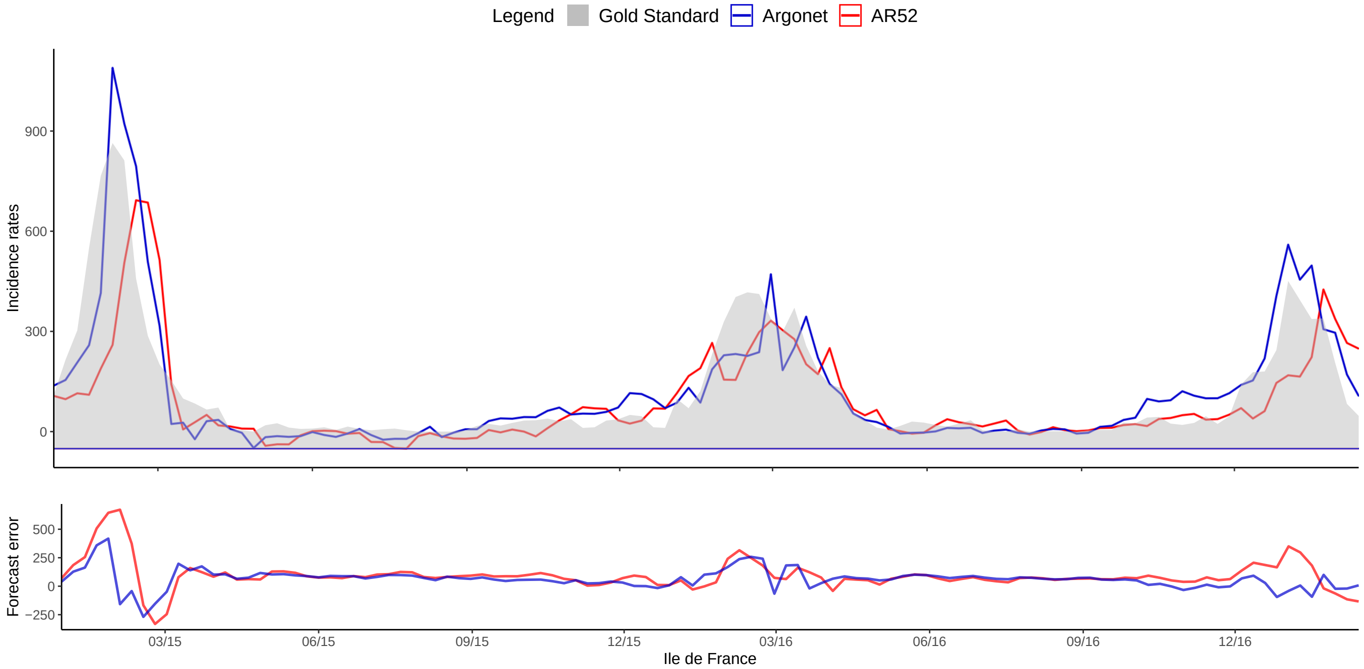

Ile de France Two-week estimate

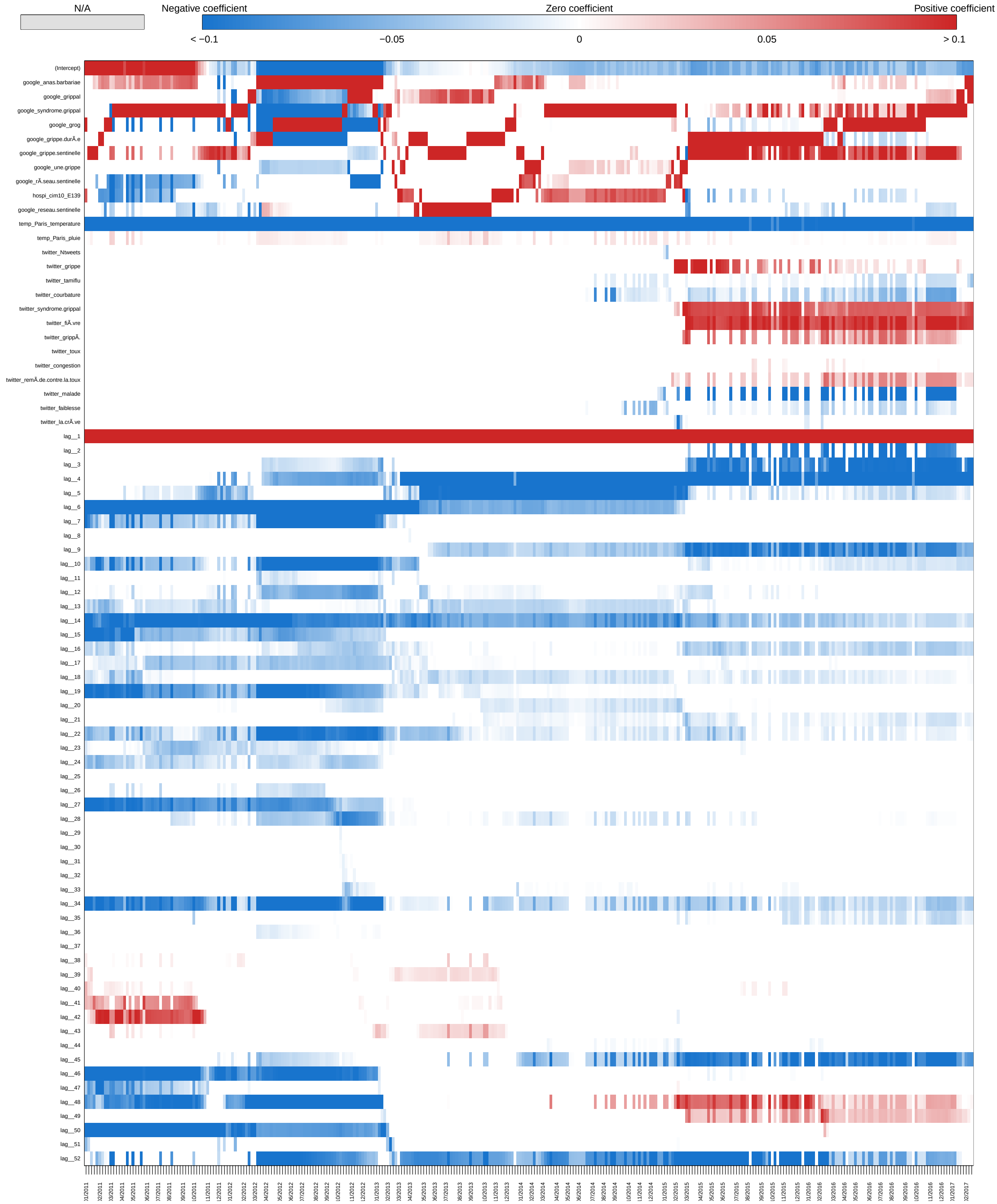

Coefficients Ile de France Two-week estimate

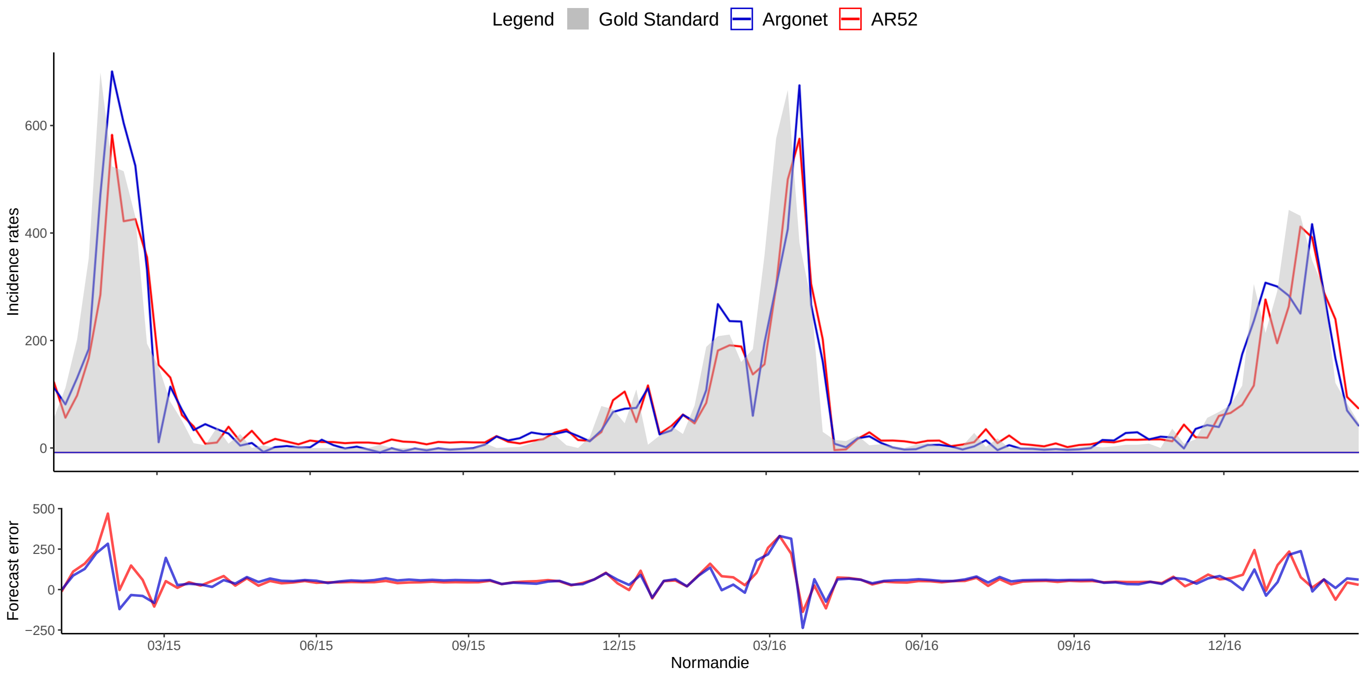

Normandie Real-time estimate

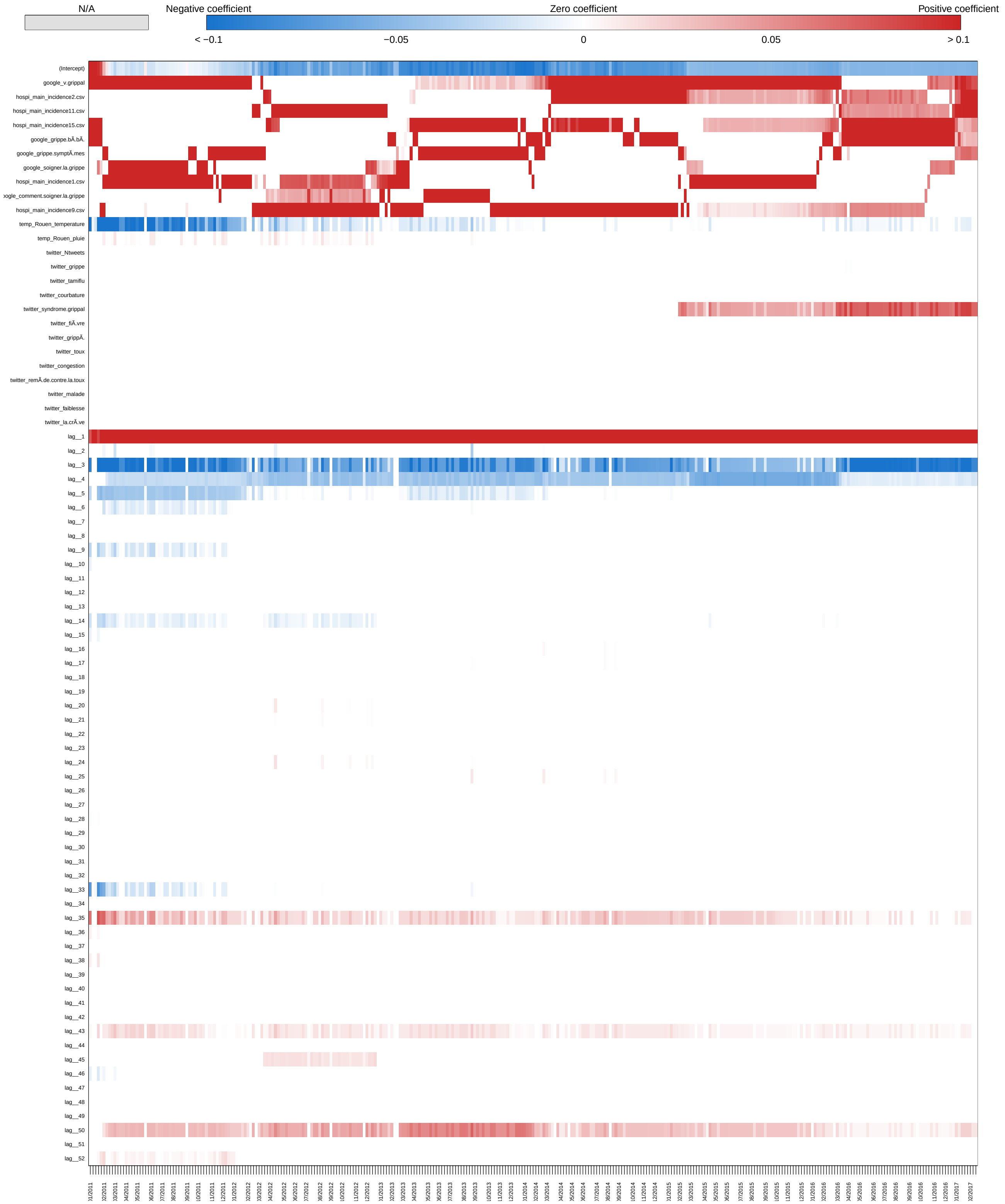

Coefficients Normandie Real-time estimate

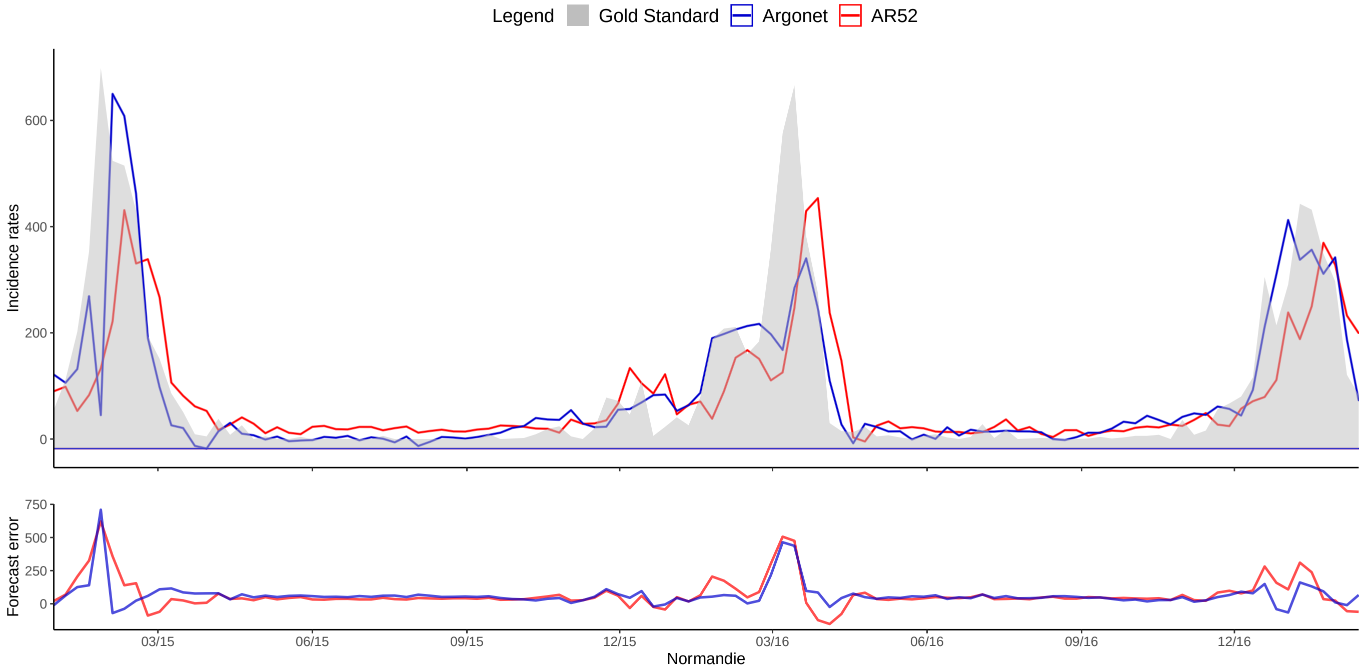

Normandie One-week estimate

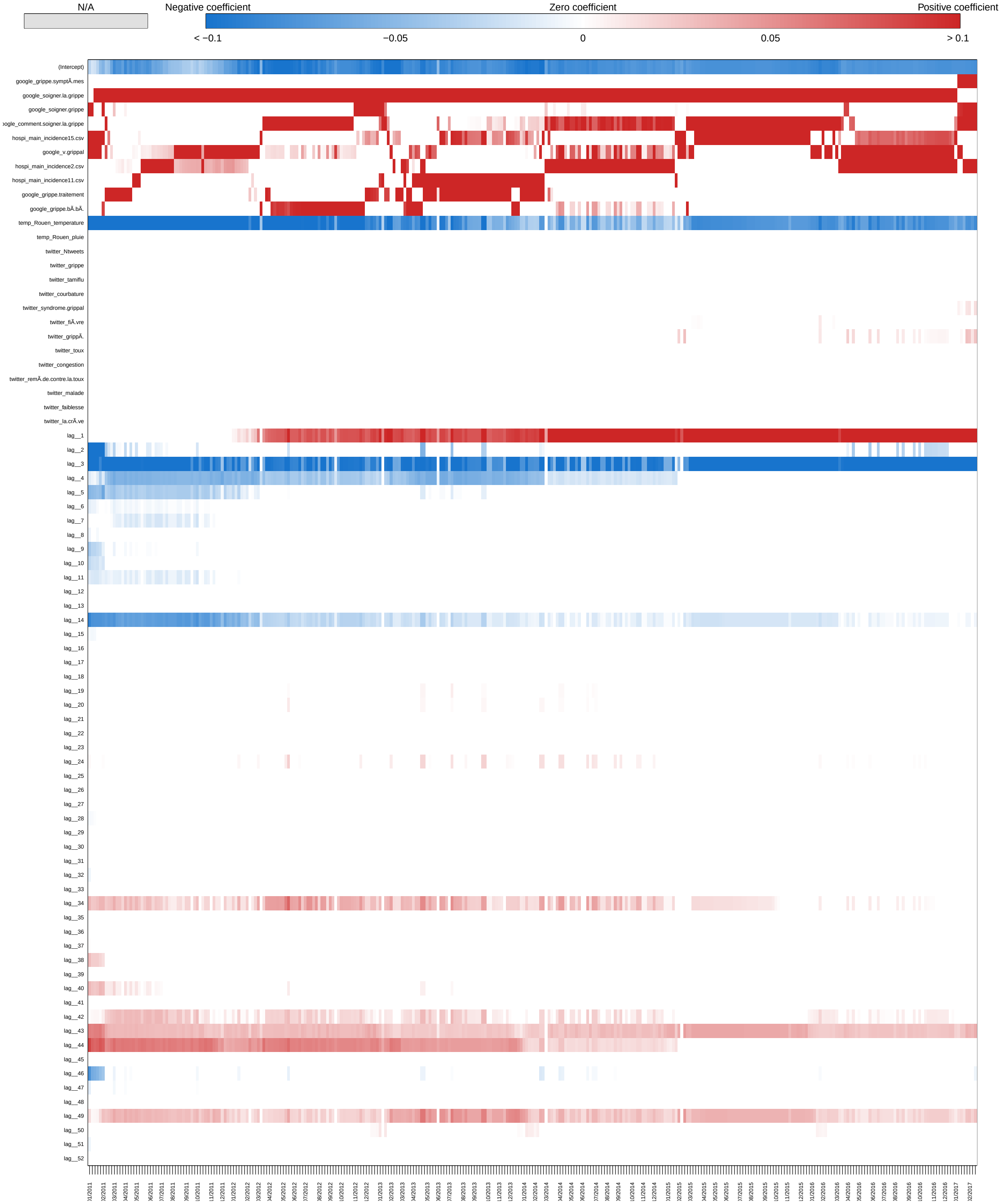

Coefficients Normandie One-week estimate

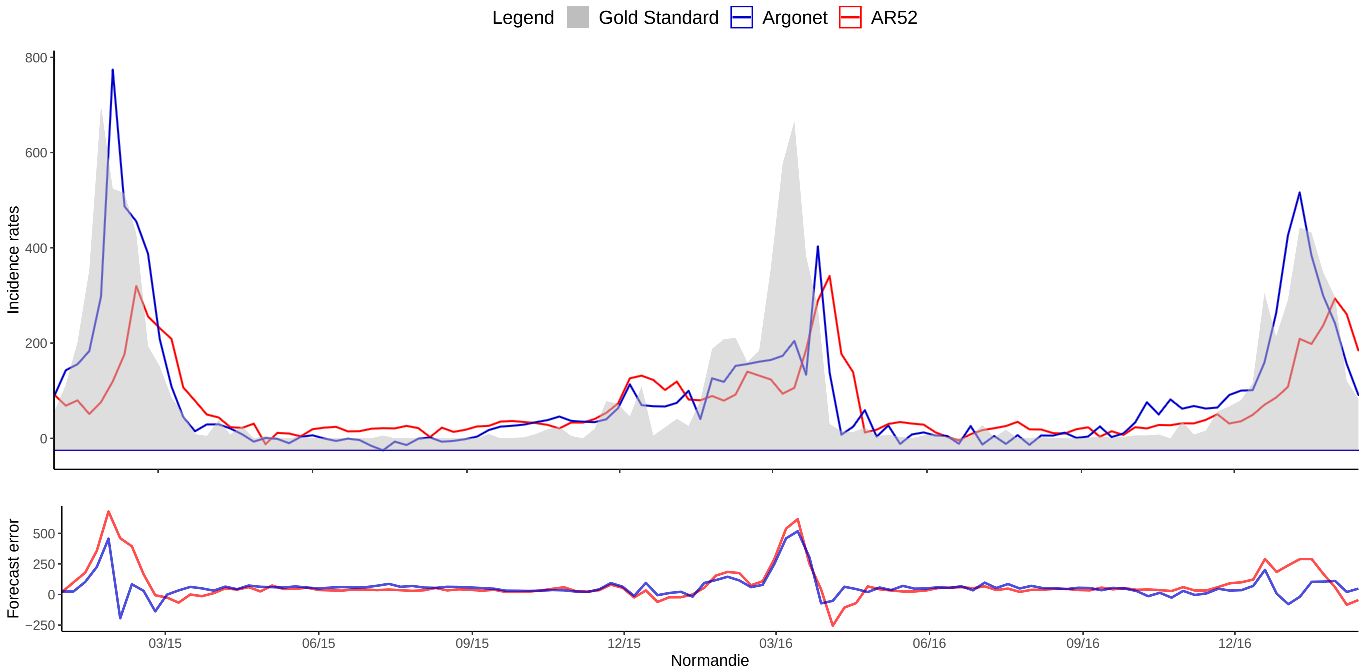

Normandie Two-week estimate

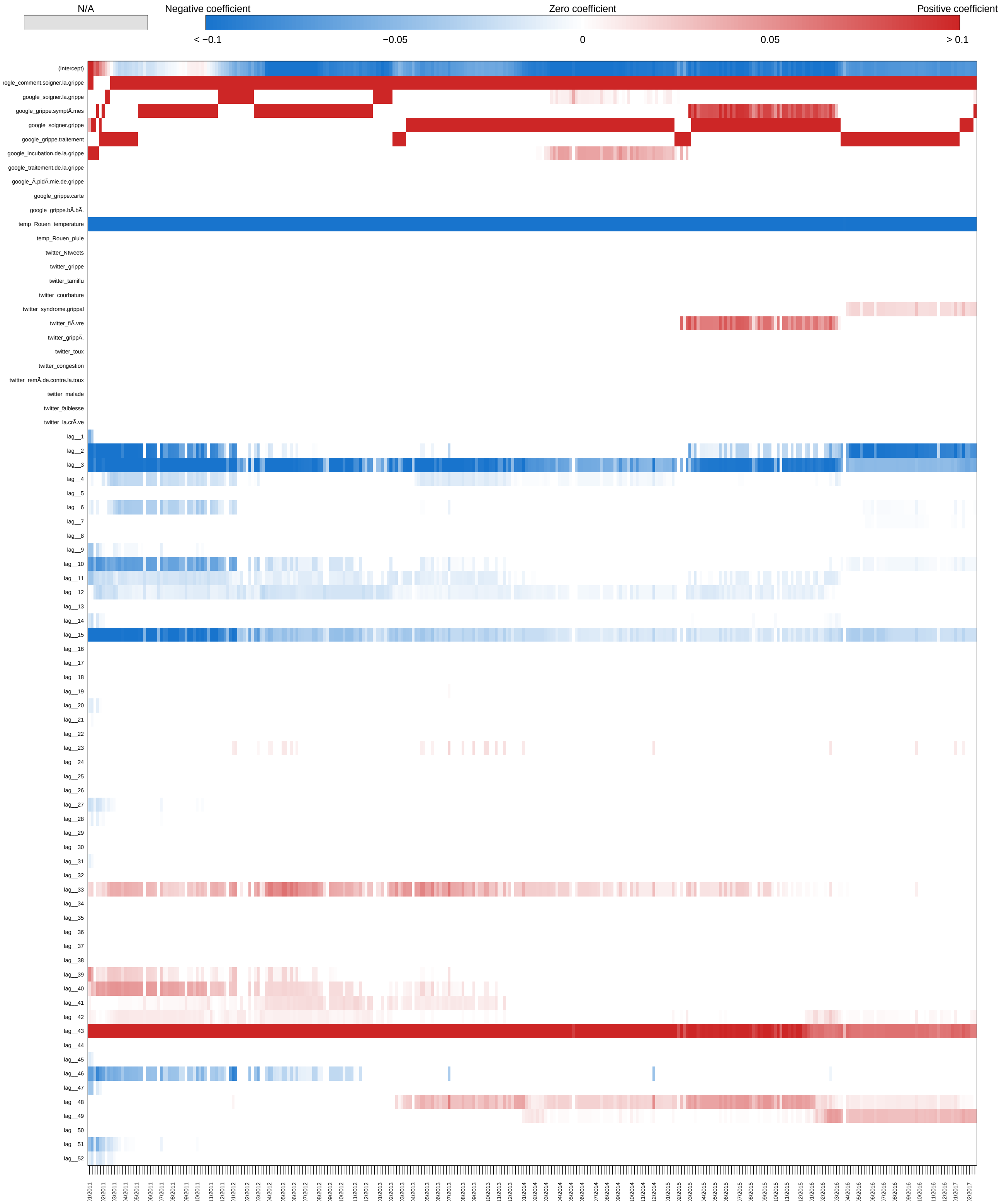

Coefficients Normandie Two-week estimate

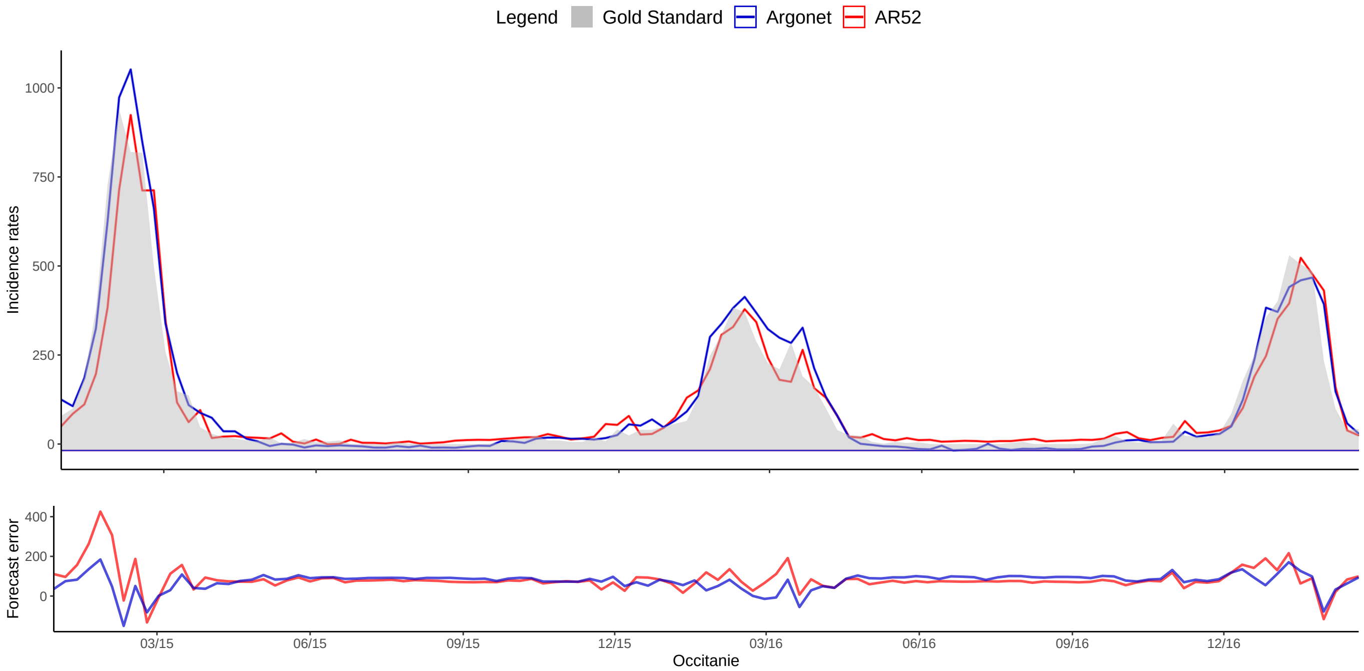

Occitanie Real-time estimate

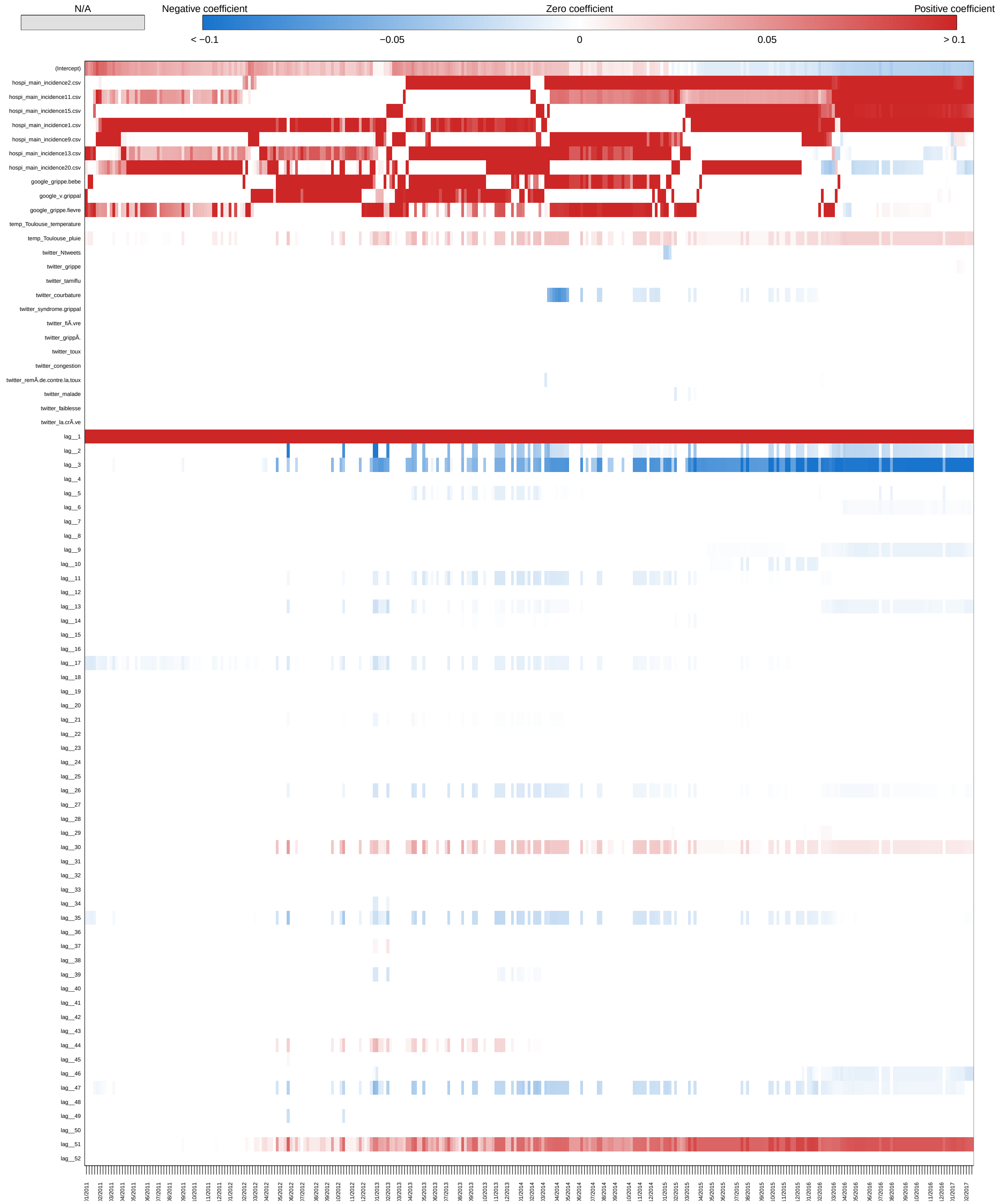

Coefficients Occitanie Real-time estimate

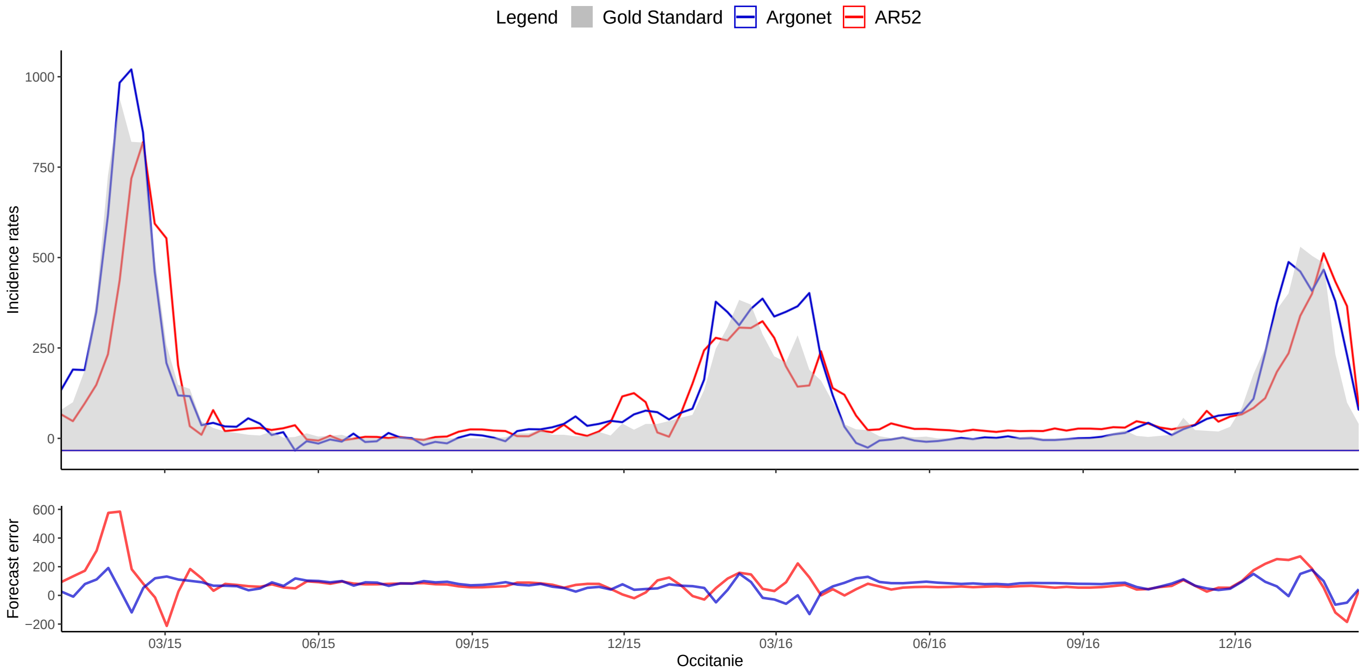

Occitanie One-week estimate

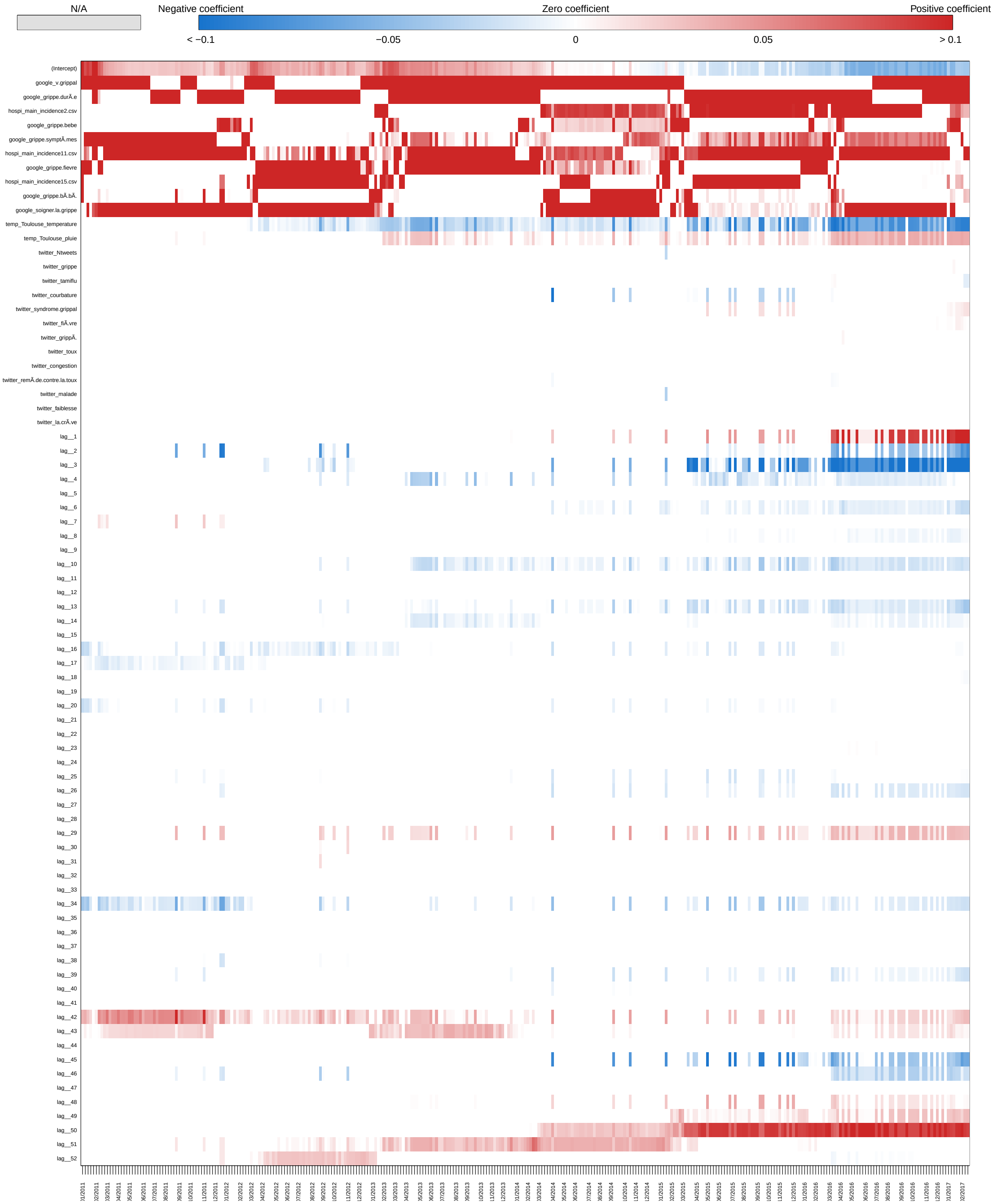

Coefficients Occitanie One-week estimate

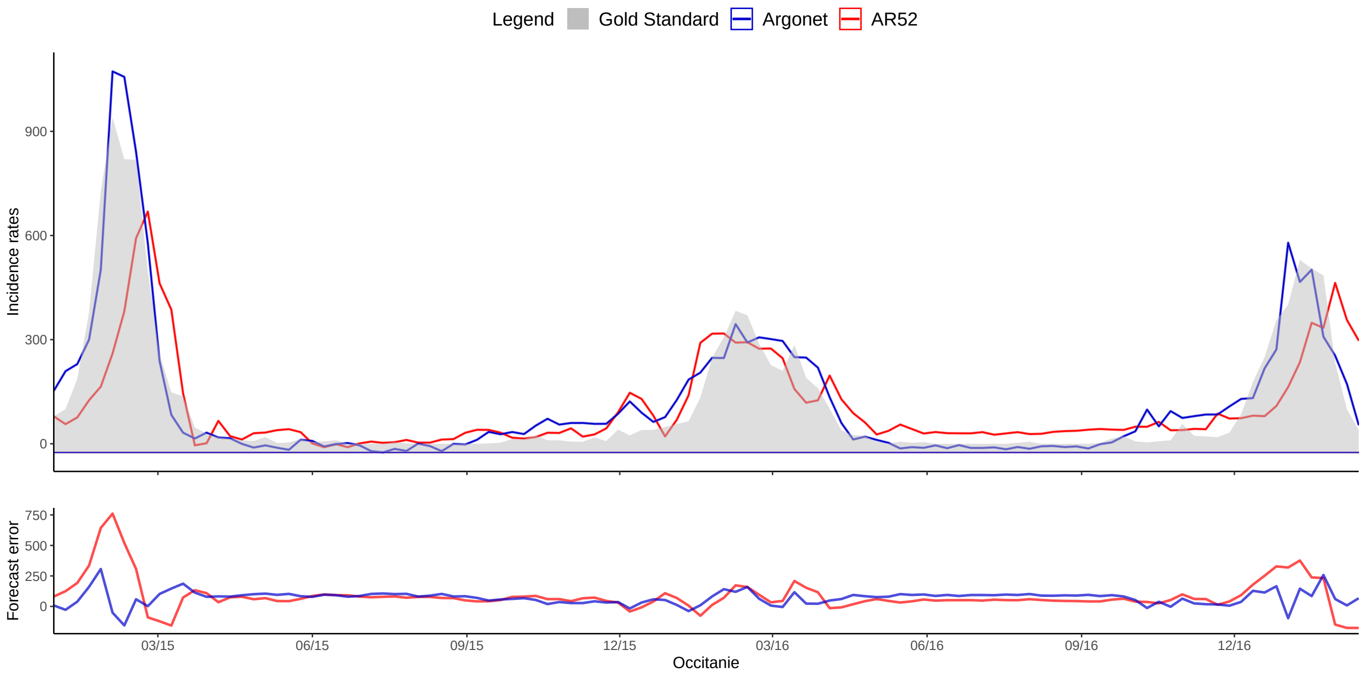

Occitanie Two-week estimate

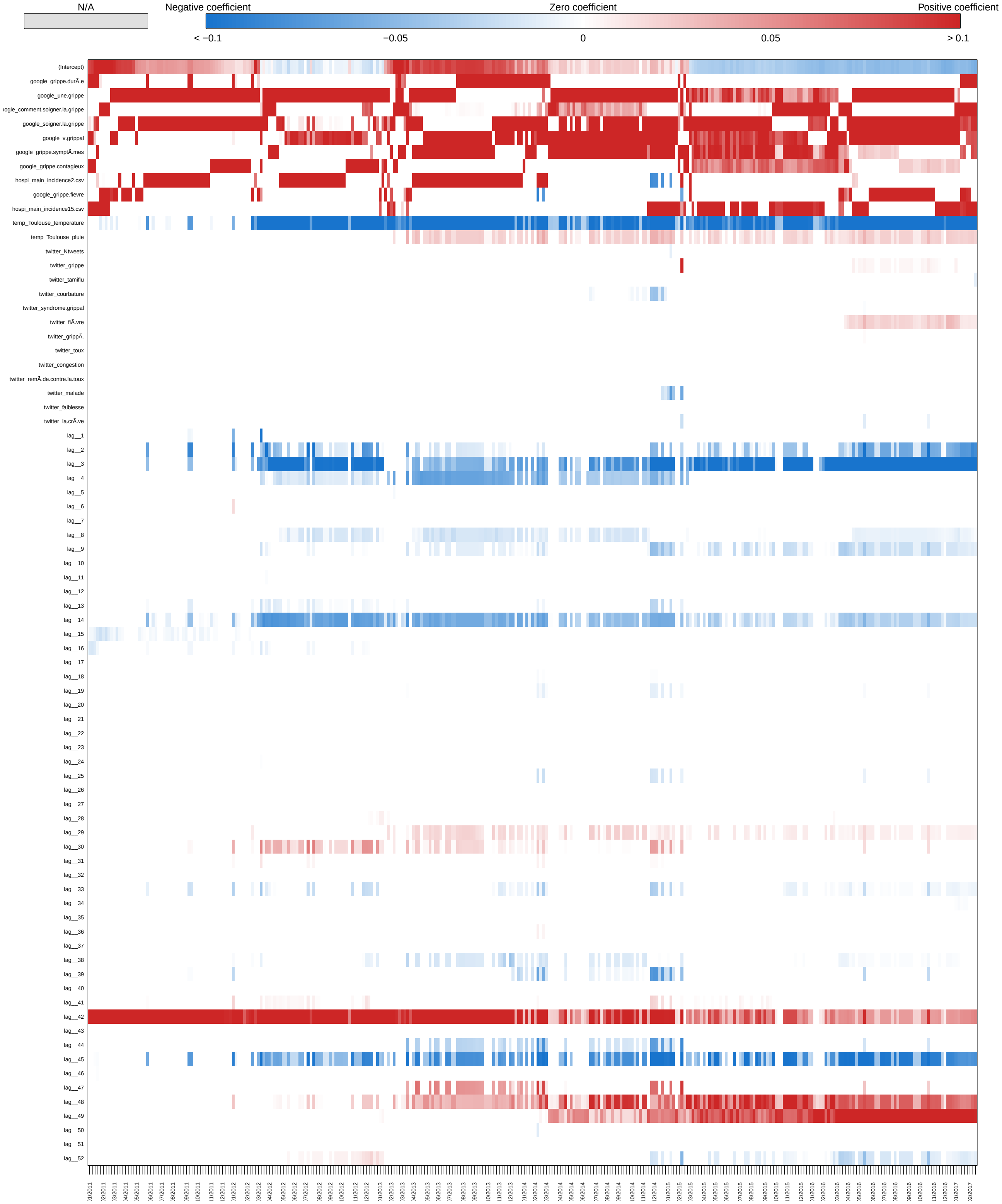

Coefficients Occitanie Two-week estimate

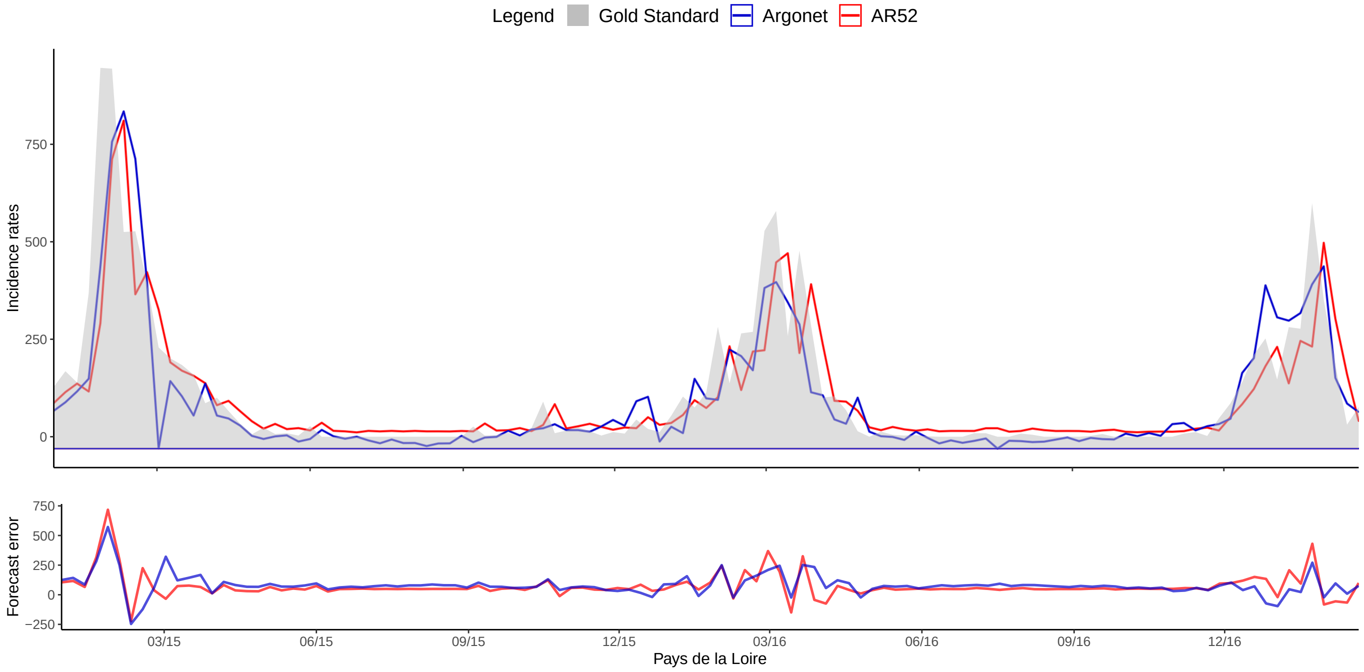

Pays de la Loire Real-time estimate

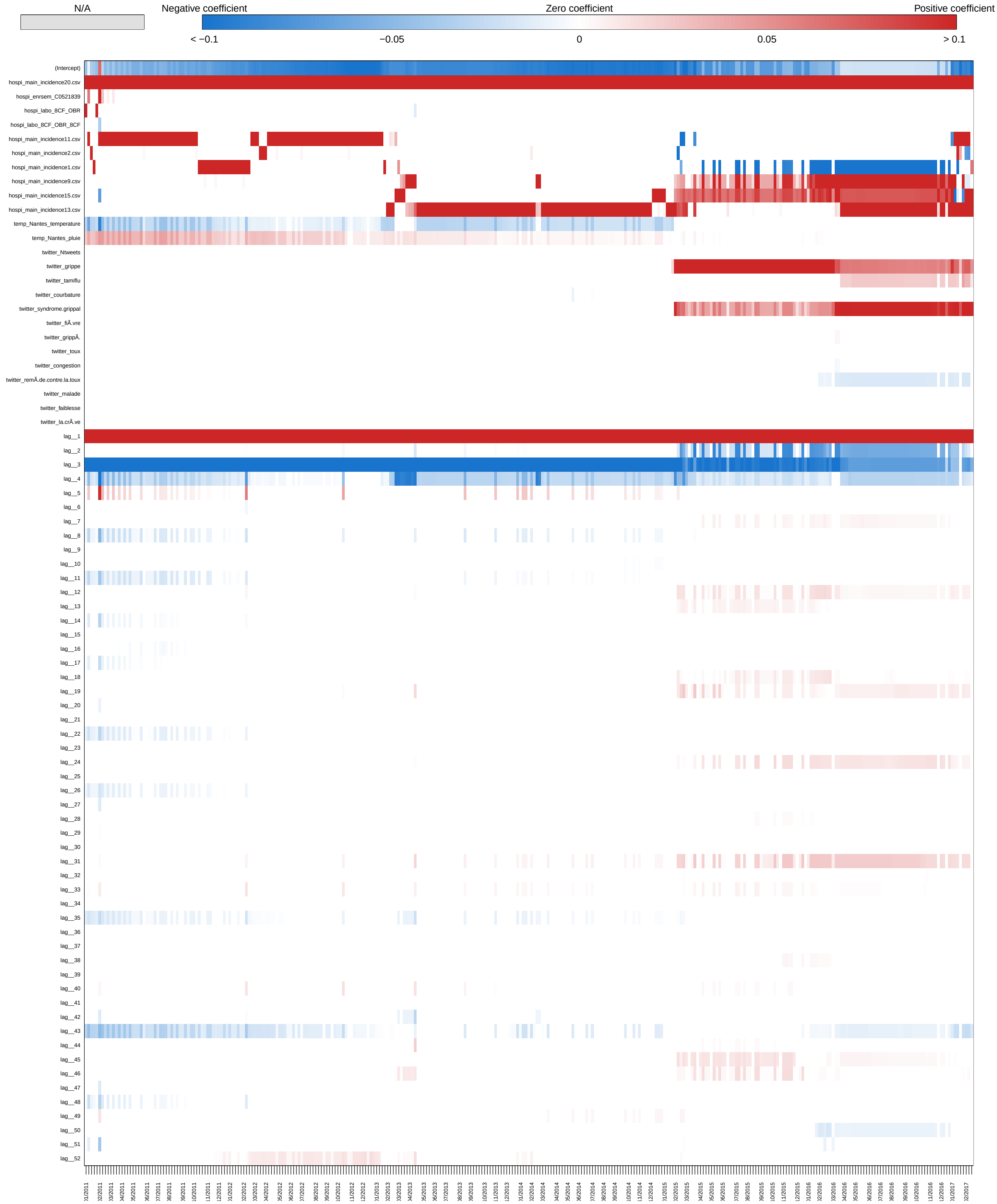

Coefficients Pays de la Loire Real-time estimate

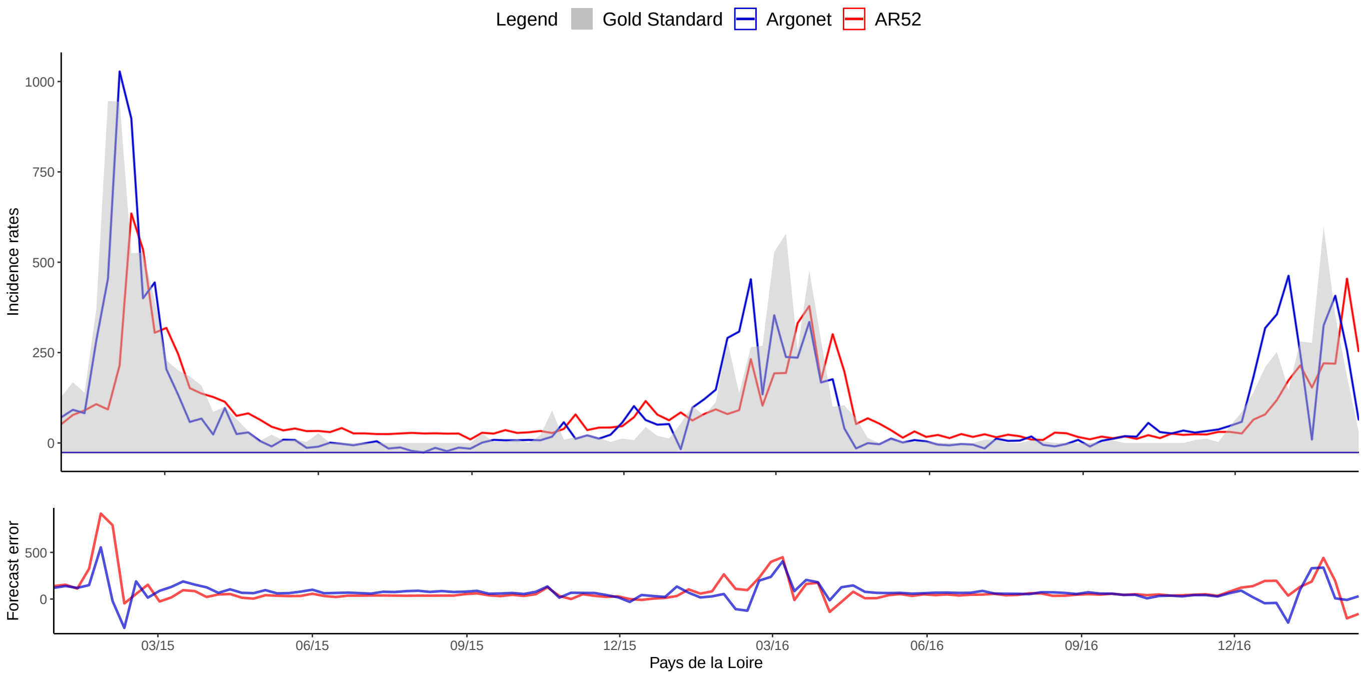

Pays de la Loire One-week estimate

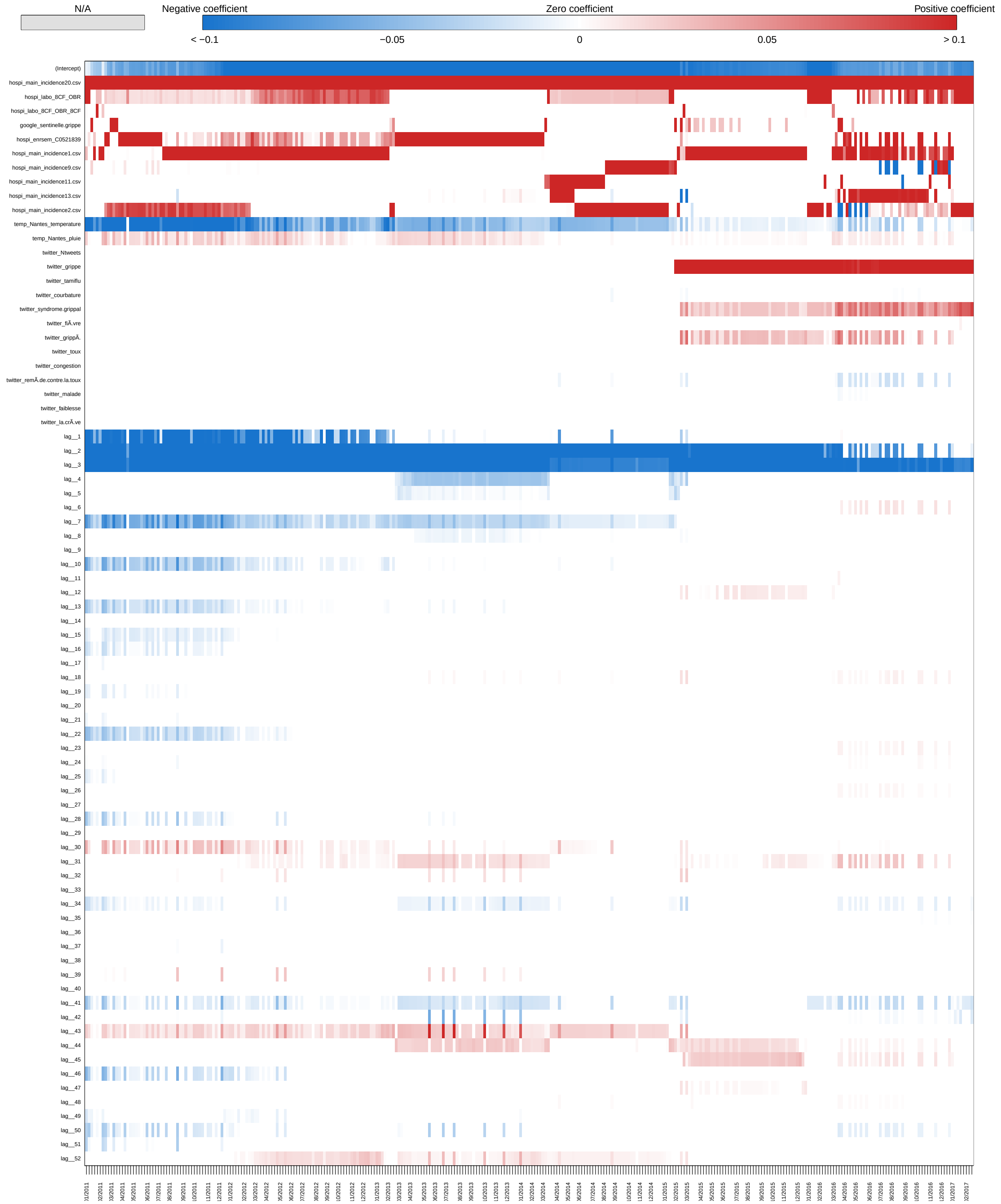

Coefficients Pays de la Loire One-week estimate

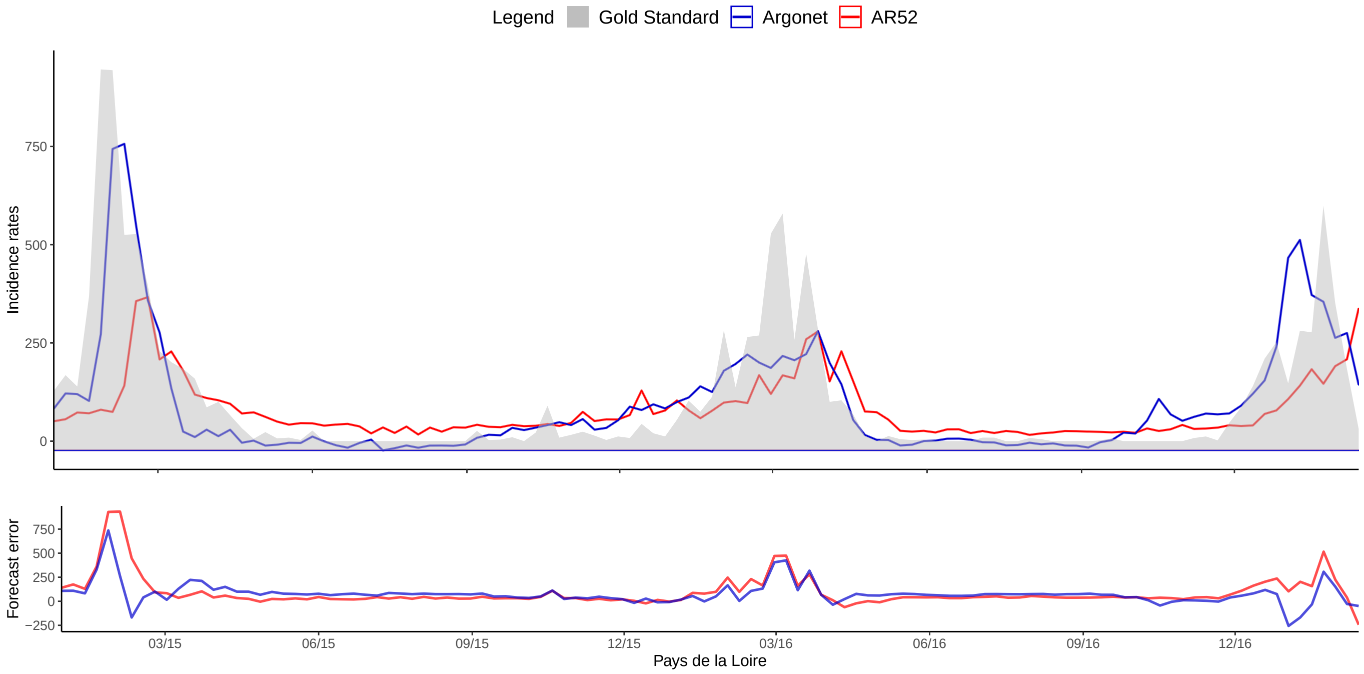

Pays de la Loire Two-week estimate

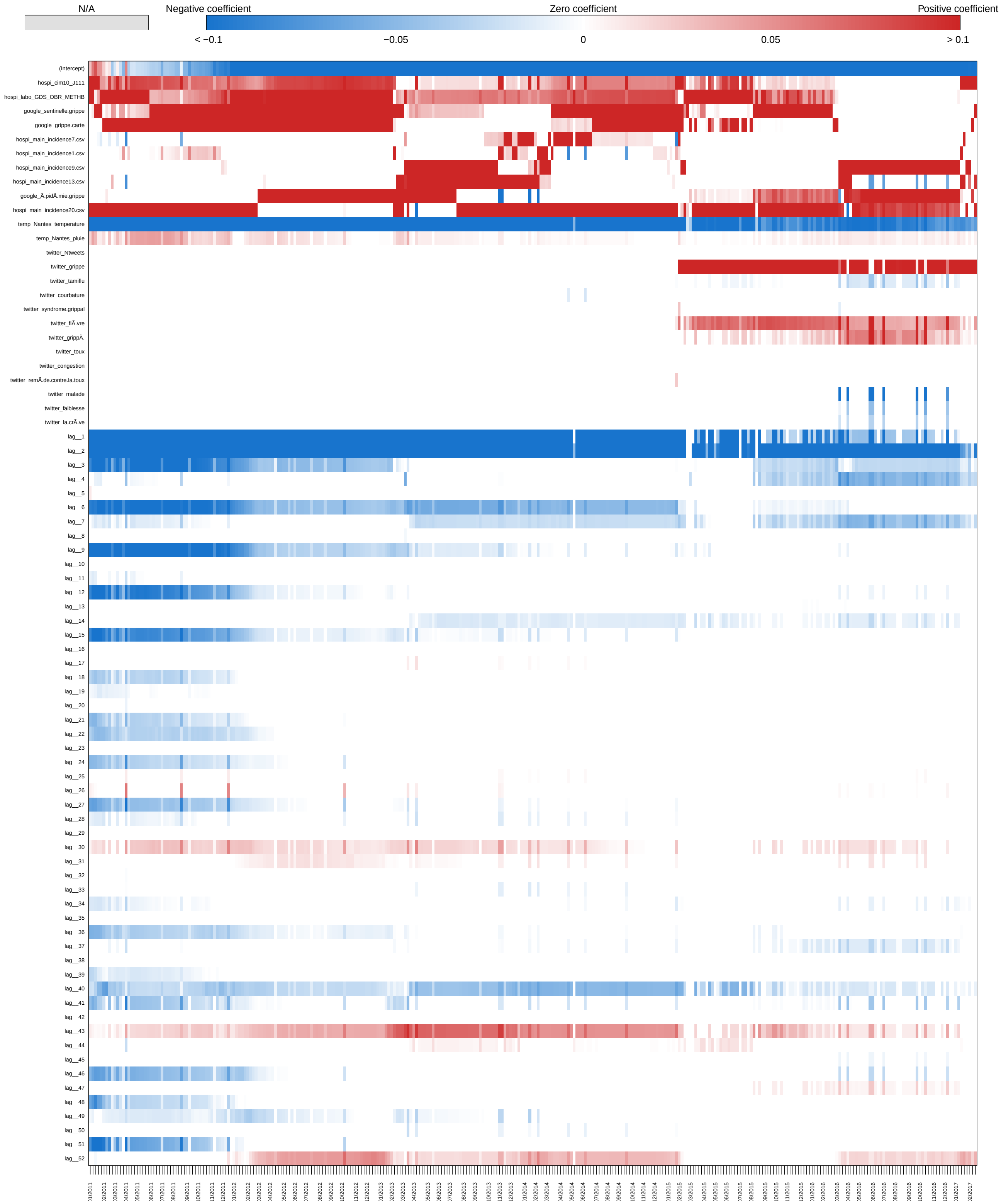

Coefficients Pays de la Loire Two-week estimate

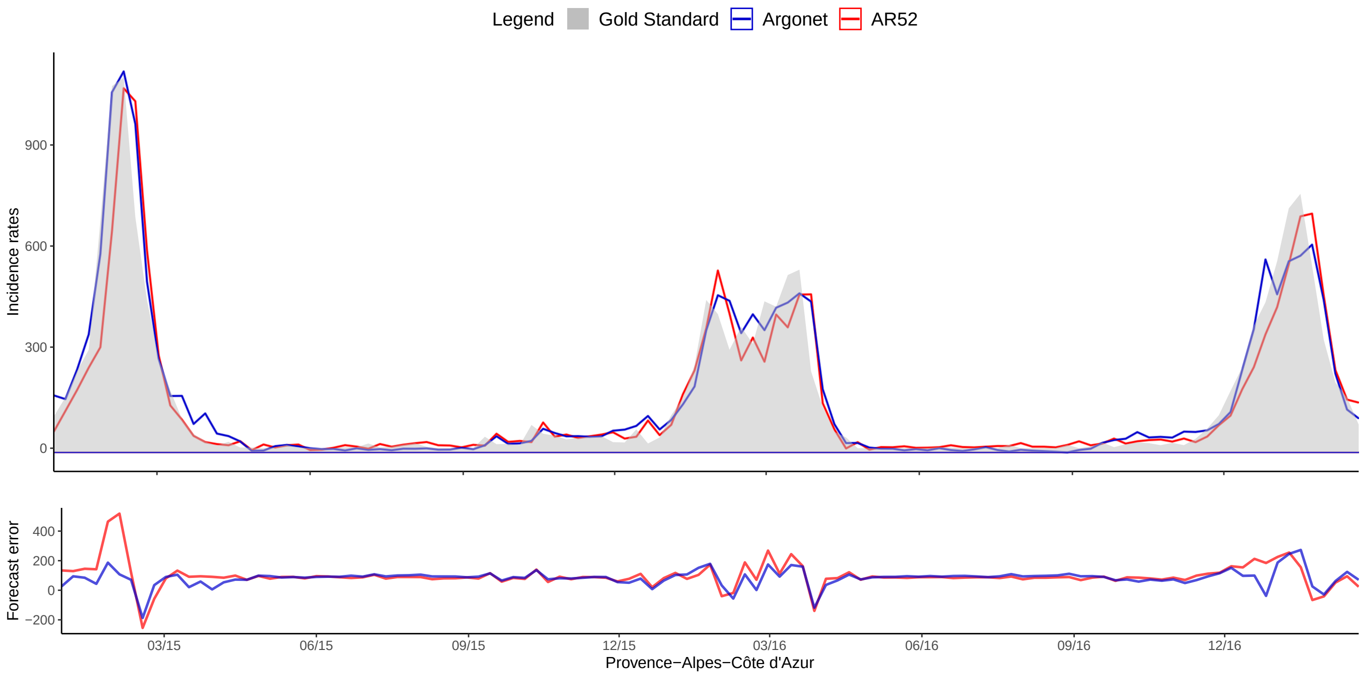

Provence Alpes Côte d'Azur Real-time estimate

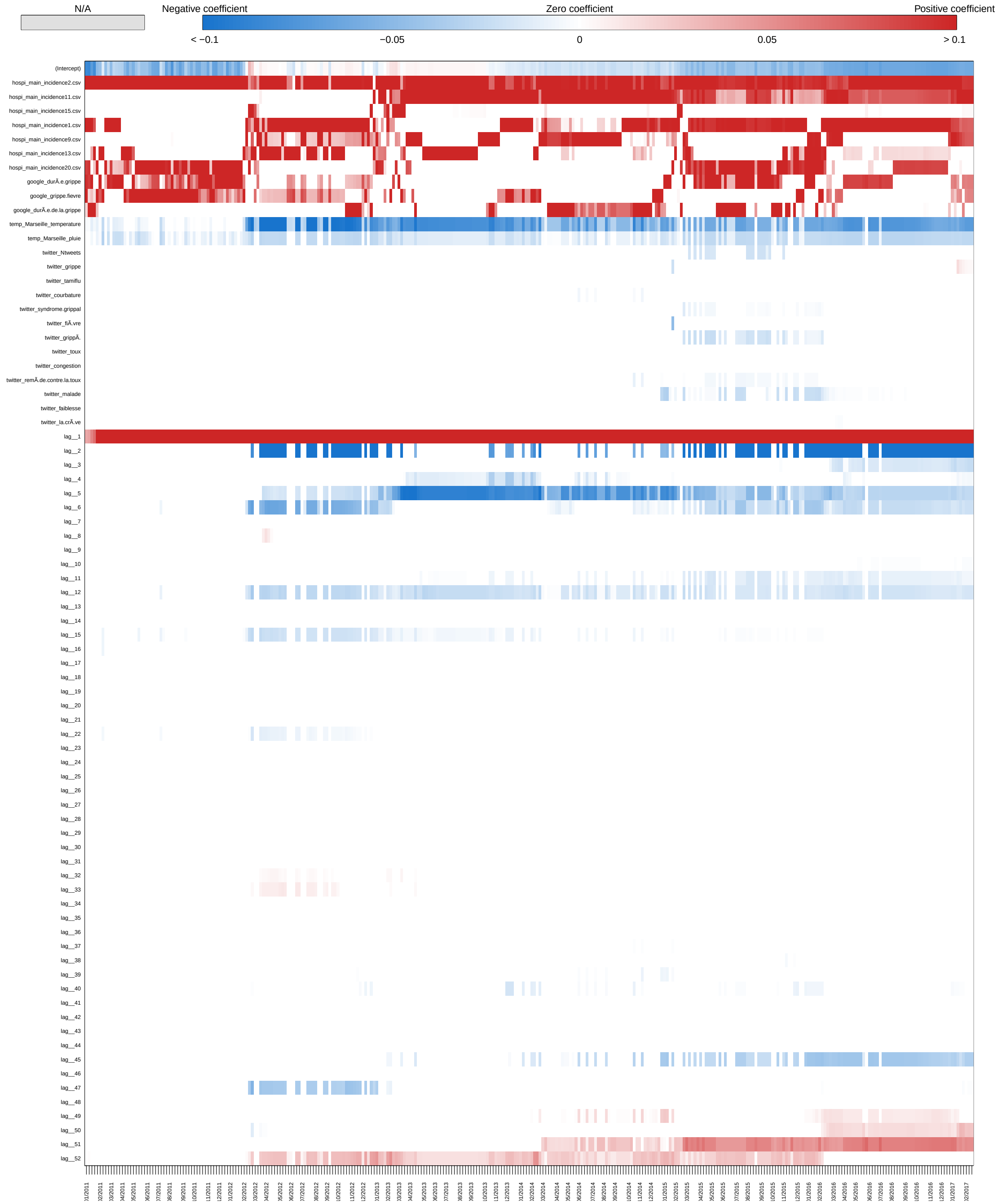

Coefficients Provence Alpes Côte d'Azur Real-time estimate

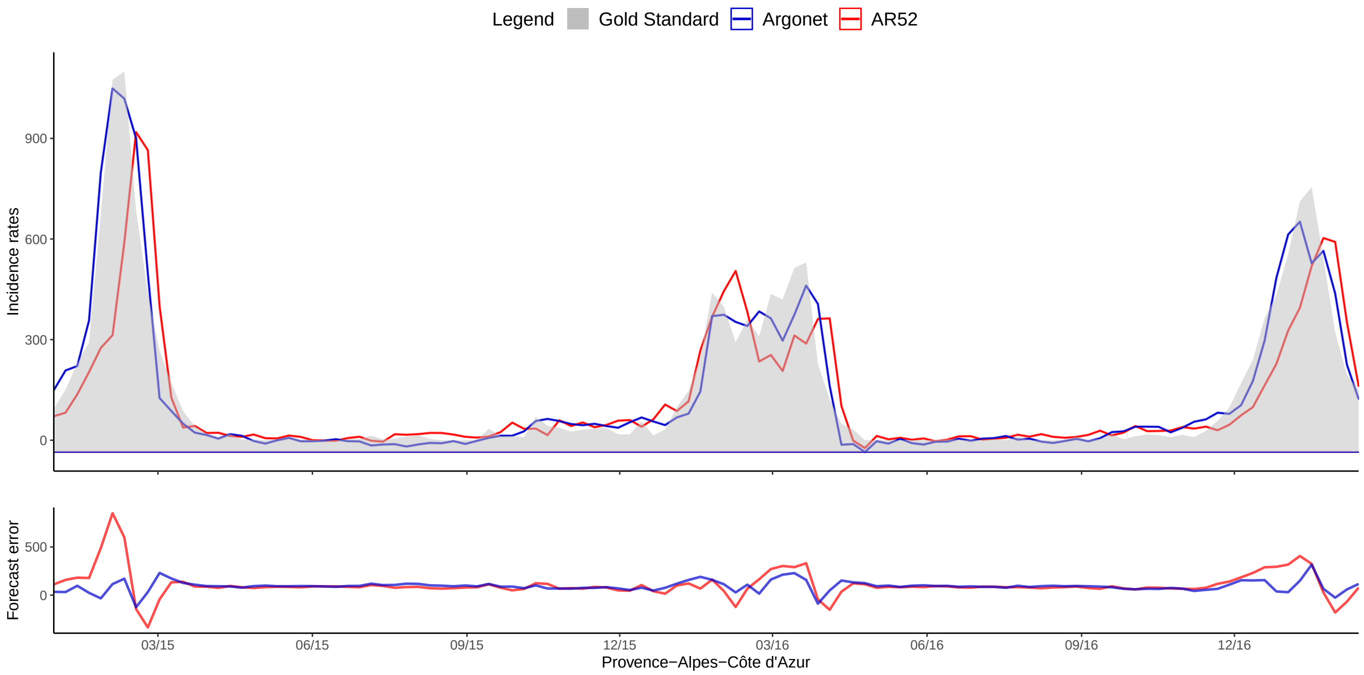

Provence Alpes Côte d'Azur One-week estimate

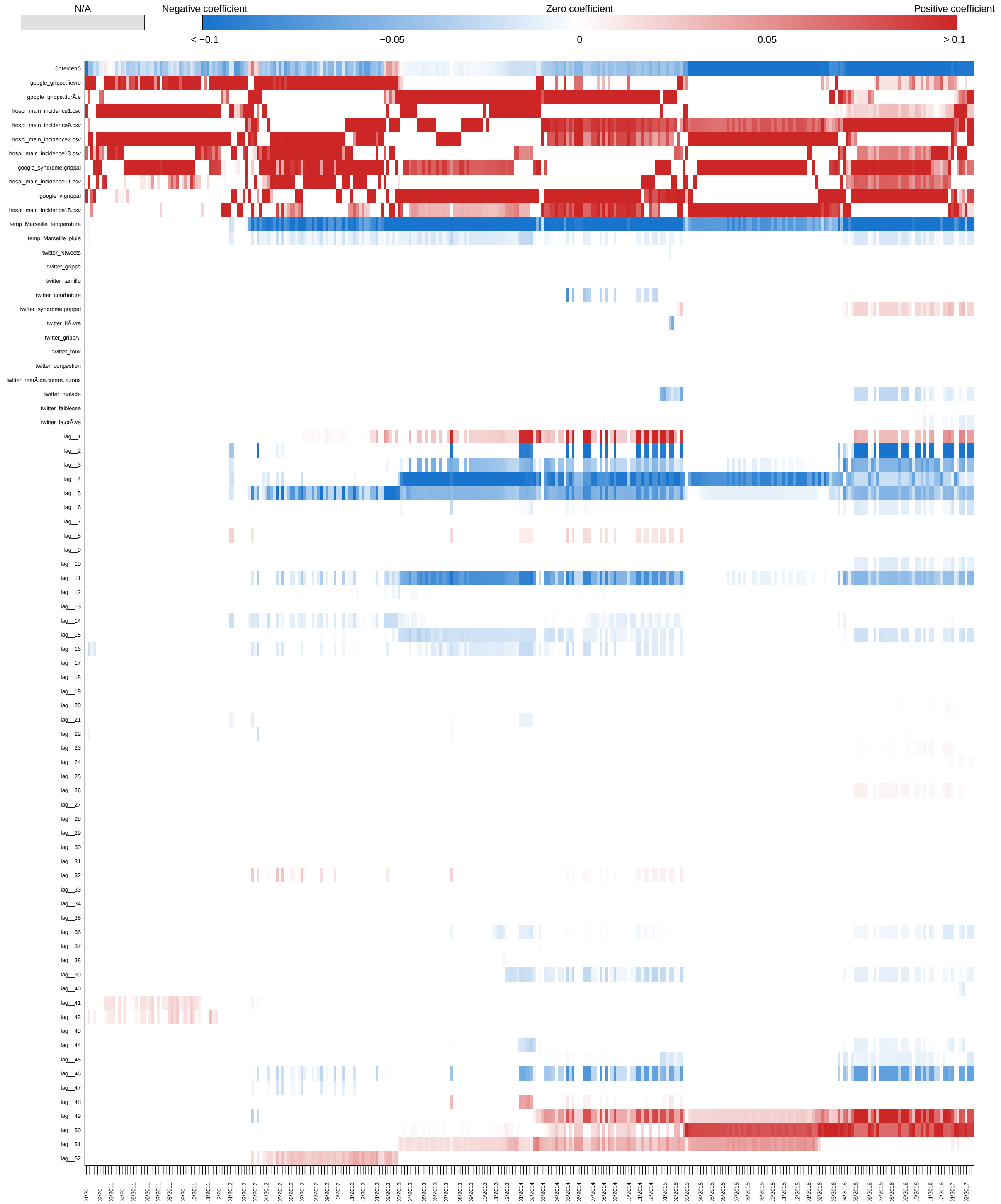

Coefficients Provence Alpes Côte d'Azur One-week estimate

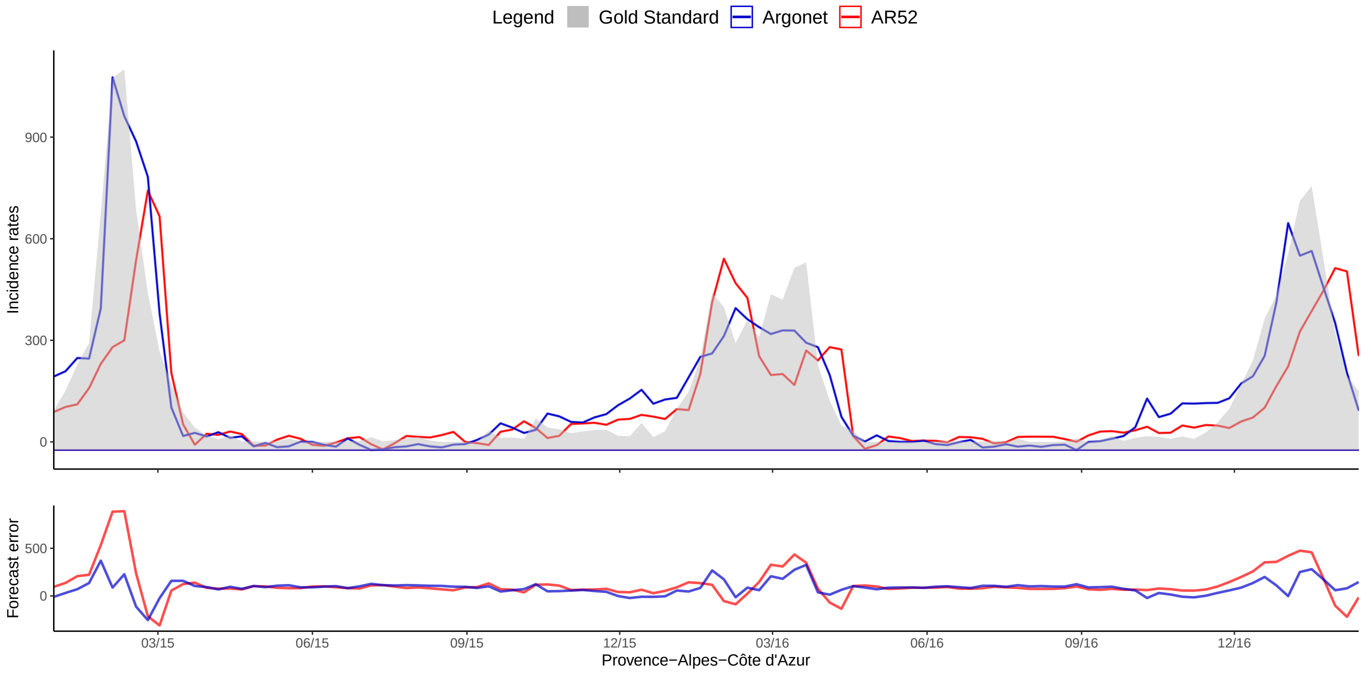

Provence Alpes Côte d'Azur Two-week estimate

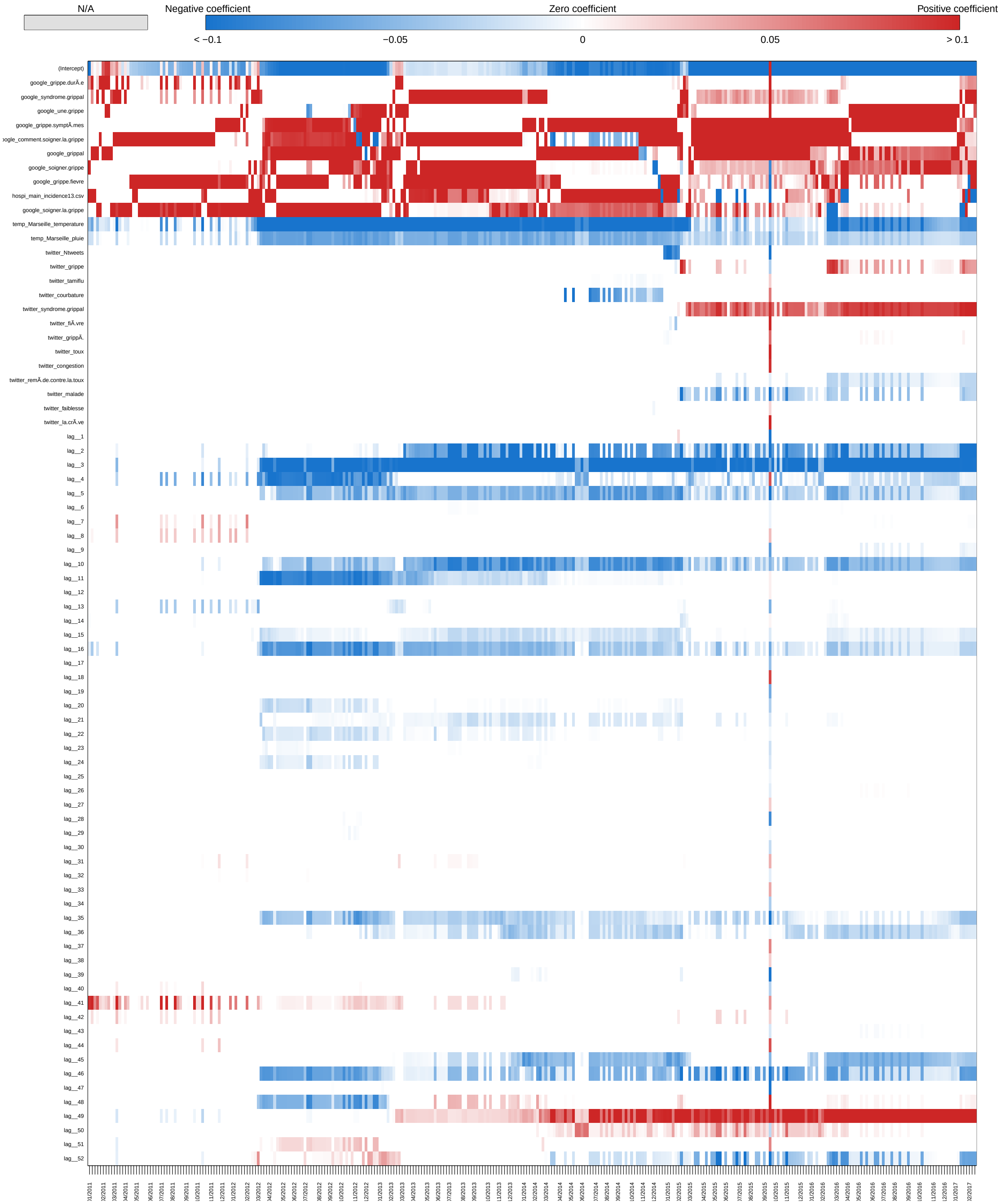

Coefficients Provence Alpes Côte d'Azur Two-week estimate

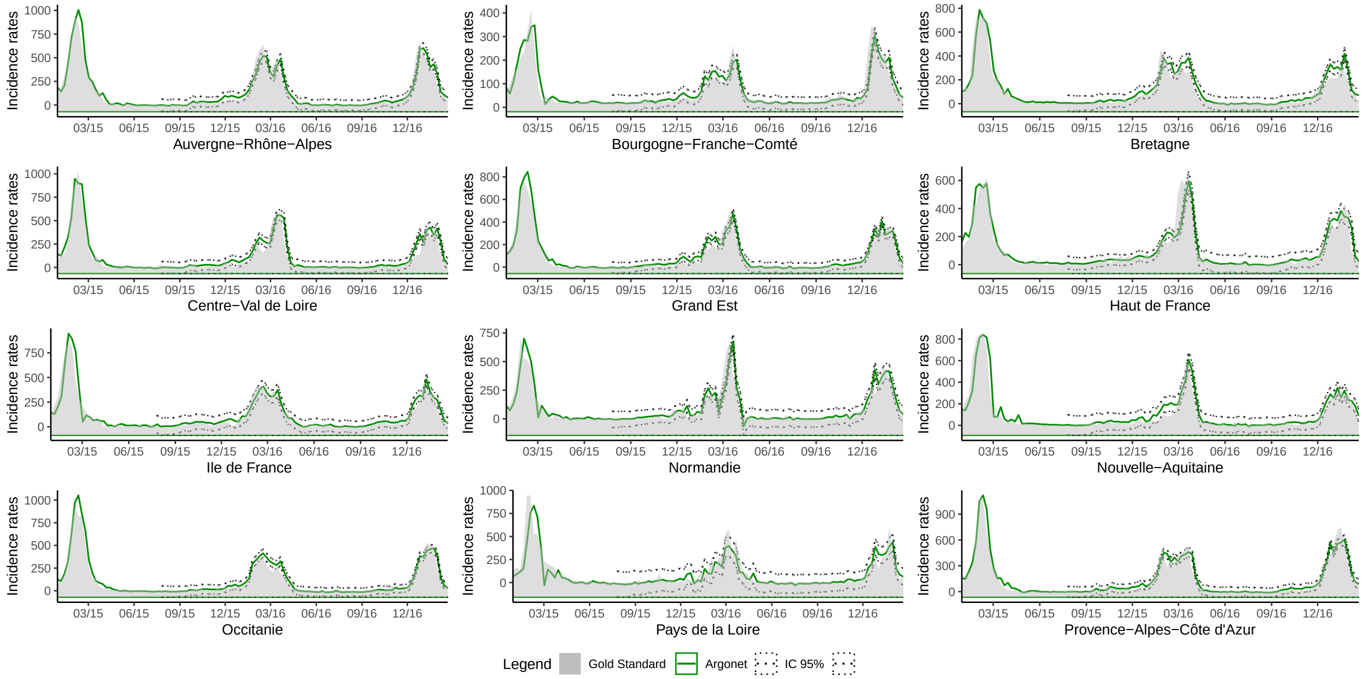

ARGONet real-time estimates and its 95% confidence interval $[\hat{y}_t - RMSE; \hat{y}_t + RMSE]$

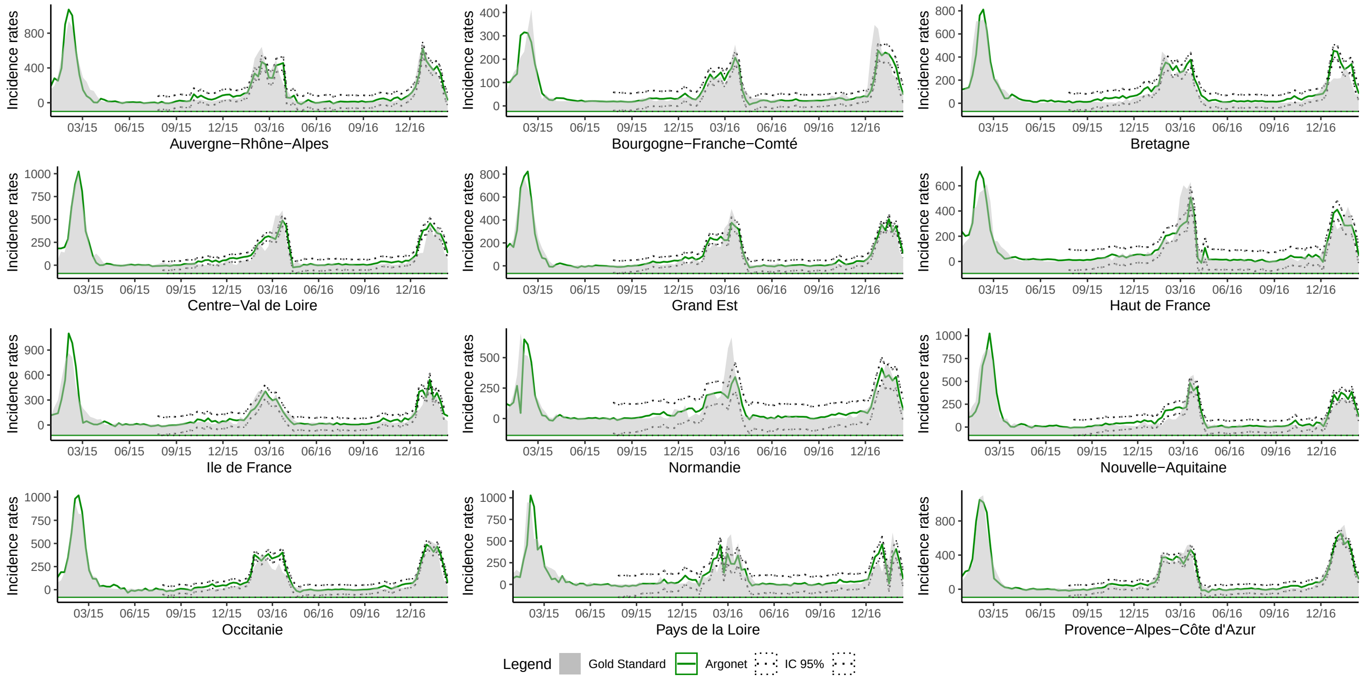

ARGONet one-week estimates and its 95% confidence interval $[\hat{y}_t - RMSE; \hat{y}_t + RMSE]$

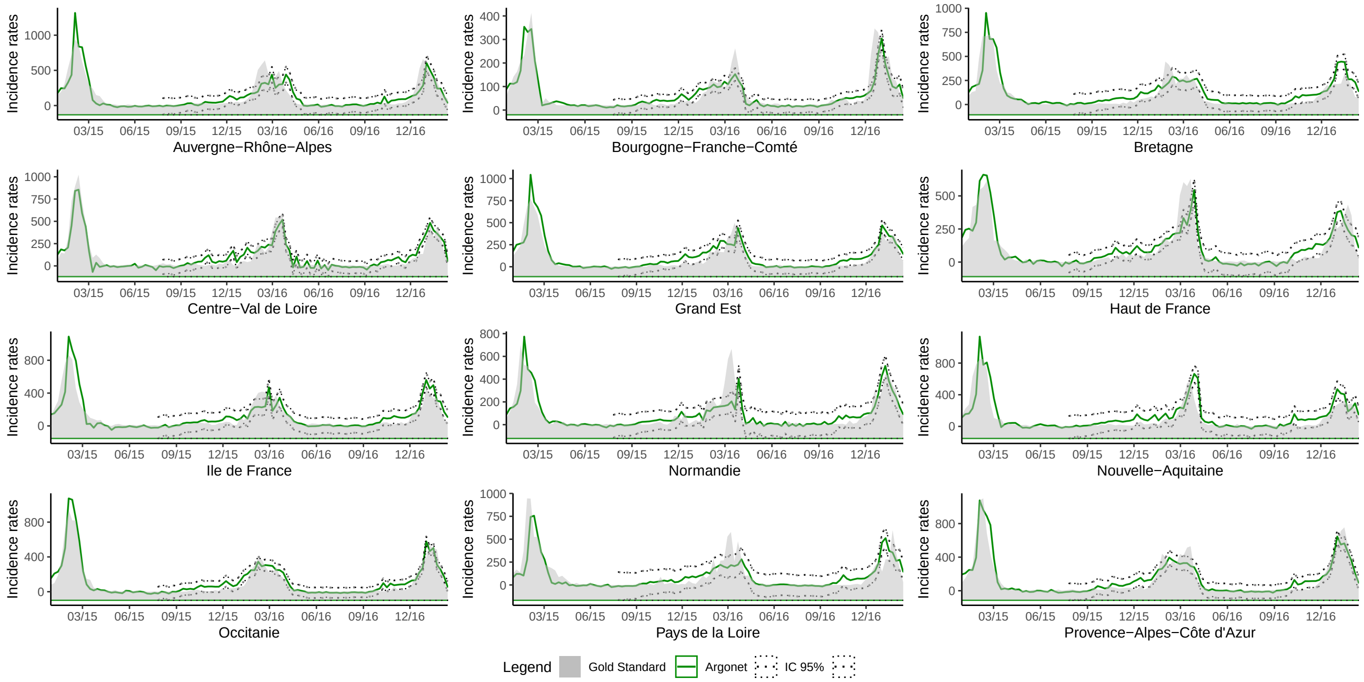

ARGONet two-week estimates and its 95% confidence interval $[\hat{y}_t - RMSE; \hat{y}_t + RMSE]$

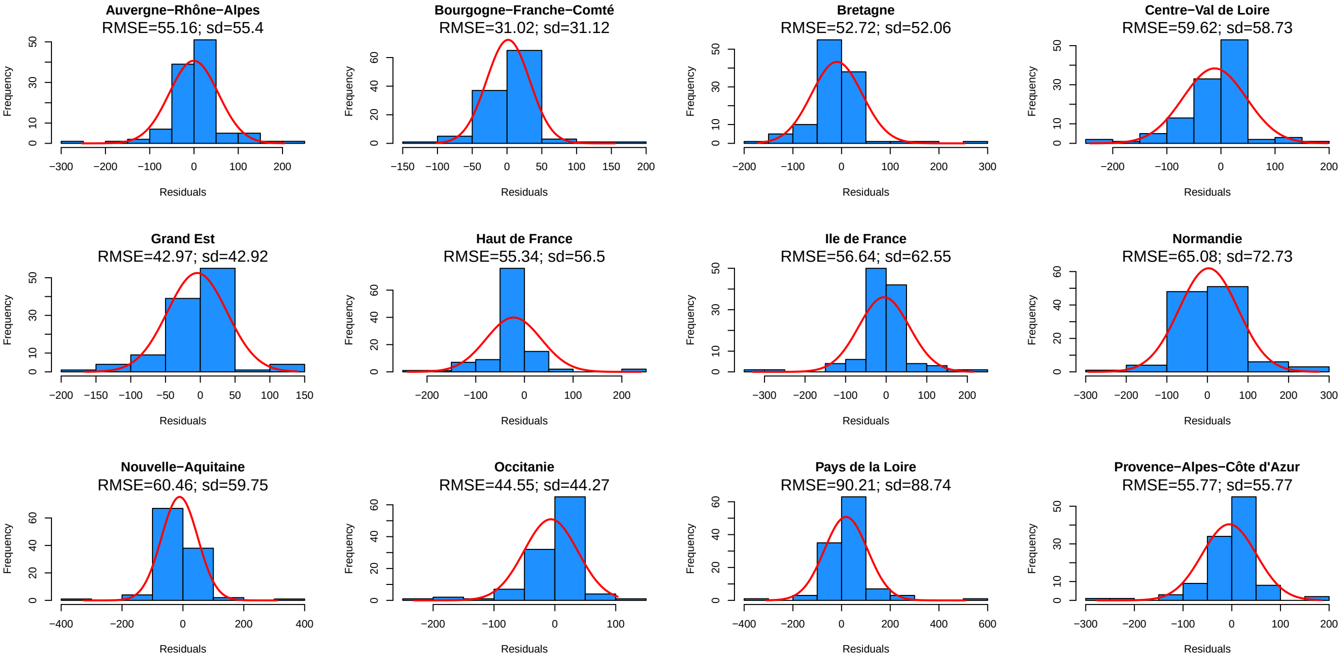

Distribution of the residuals $(y_t - \hat{y}_t)$ for the real-time estimates obtained with ARGONet, showing that the RMSE is comparable to the standard deviation of the gaussian distribution.

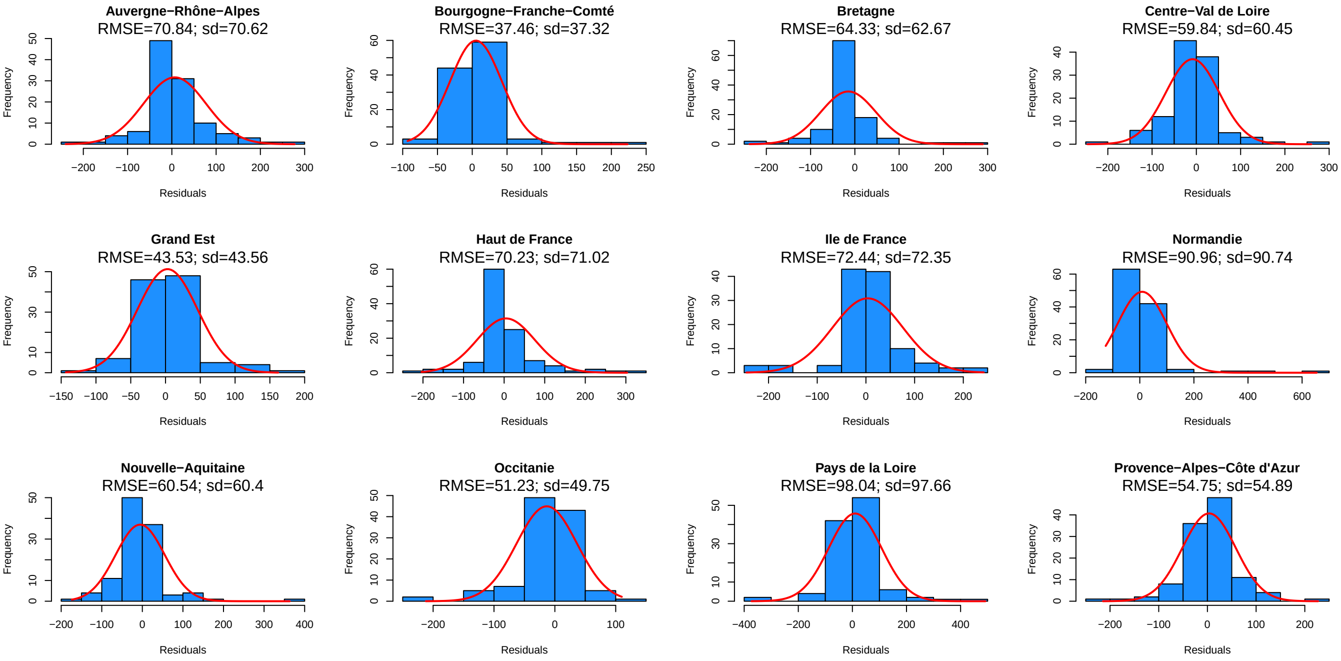

Distribution of the residuals $(y_t - \hat{y}_t)$ for the one-week estimates obtained with ARGONet, showing that the RMSE is comparable to the standard deviation of the gaussian distribution.

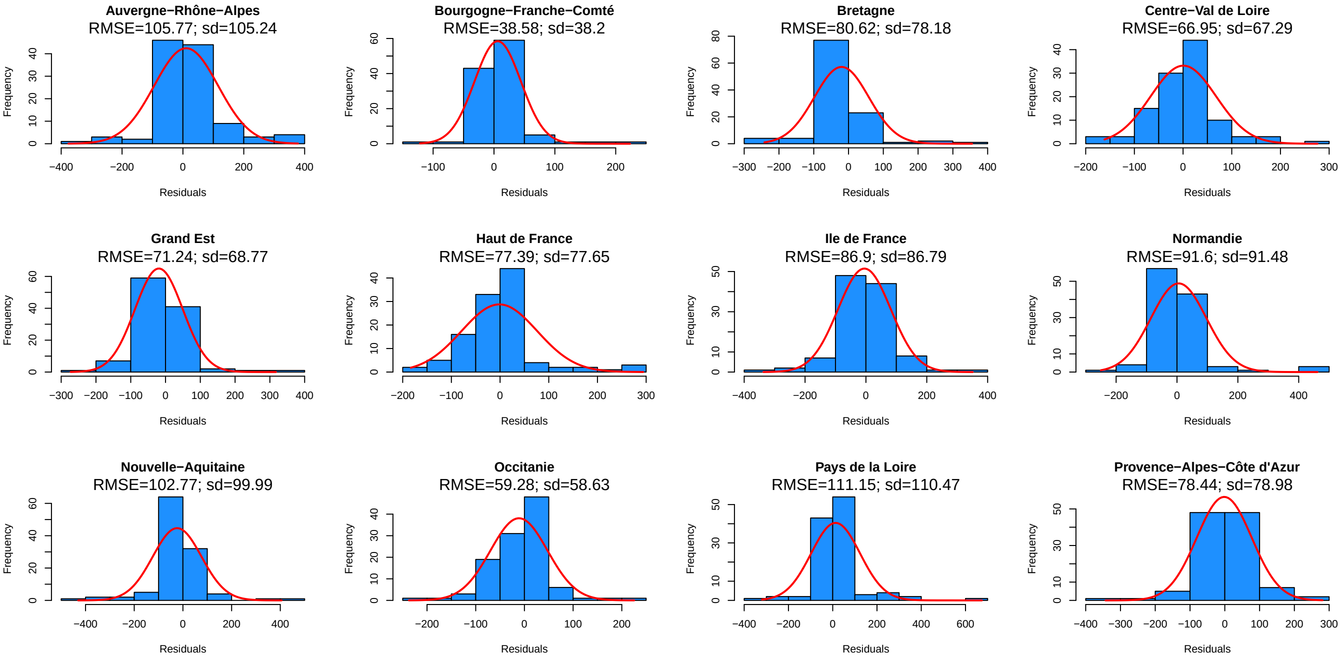

Distribution of the residuals $(y_t - \hat{y}_t)$ for the two-week estimates obtained with ARGONet, showing that the RMSE is comparable to the standard deviation of the gaussian distribution.
